# The long-term use of foot orthoses affects walking kinematics and kinetics of children with flexible flat feet: A randomized controlled trial

**DOI:** 10.1371/journal.pone.0205187

**Published:** 2018-10-09

**Authors:** AmirAli Jafarnezhadgero, Morteza Madadi-Shad, Seyed Majid Alavi-Mehr, Urs Granacher

**Affiliations:** 1 Department of Physical Education and Sport Sciences, University of Mohaghegh Ardabili, Ardabil, Iran; 2 Sport Biomechanics Department, Bu-Ali Sina University, Hamedan, Iran; 3 Division of Training and Movement Sciences, Research Focus Cognition Sciences, University of Potsdam, Potsdam, Germany; National Center of Medicine and Science in Sport, TUNISIA

## Abstract

**Background:**

Due to inconclusive evidence on the effects of foot orthoses treatment on lower limb kinematics and kinetics in children, studies are needed that particularly evaluate the long-term use of foot orthoses on lower limb alignment during walking. Thus, the main objective of this study was to evaluate the effects of long-term treatment with arch support foot orthoses versus a sham condition on lower extremity kinematics and kinetics during walking in children with flexible flat feet.

**Methods:**

Thirty boys aged 8–12 years with flexible flat feet participated in this study. While the experimental group (n = 15) used medial arch support foot orthoses during everyday activities over a period of four months, the control group (n = 15) received flat 2-mm-thick insoles (i.e., sham condition) for the same time period. Before and after the intervention period, walking kinematics and ground reaction forces were collected.

**Results:**

Significant group by time interactions were observed during walking at preferred gait speed for maximum ankle eversion, maximum ankle internal rotation angle, minimum knee abduction angle, maximum knee abduction angle, maximum knee external rotation angle, maximum knee internal rotation angle, maximum hip extension angle, and maximum hip external rotation angle in favor of the foot orthoses group. In addition, statistically significant group by time interactions were detected for maximum posterior, and vertical ground reaction forces in favor of the foot orthoses group.

**Conclusions:**

The long-term use of arch support foot orthoses proved to be feasible and effective in boys with flexible flat feet to improve lower limb alignment during walking.

## Introduction

Epidemiological studies indicate that 4% of children aged 10 years suffer from flat feet (FF) [1]. Of those children with FF, 10% receive treatment for the prevention of secondary deformity during adulthood [2]. It has previously been shown that children with flexible FF compared with aged-matched healthy peers are more likely to suffer from pain or discomfort at the knee, hip, or trunk [3]. A variety of intervention programs are used to treat such problems, with foot orthoses (FOs) being a popular means [4–6].

In a recent website audit, it was found that 47% of websites from Canadian Chiropractors advertise orthotics for sale [7]. There is evidence from Australian Podiatrists indicating that during the last decade, 91% prescribed either functional (individualized) or prefabricated orthotics to individuals (adults and children) with FF [8]. Moreover, it has been reported that FOs are effective means to treat a variety of painful feet conditions including the high or low arched foot, rheumatoid arthritis, and plantar fasciitis [9–11]. However, the biomechanical mechanisms underlying the therapeutic effect of FOs in children with flexible FF is still unclear.

We used arch support FOs in this study that were manufactured with a negative cast technique in subtalar joint neutral position [12]. The rationale behind the application of FOs is that they are supposed to realign the lower limb through changes in lower extremity kinematics. However, this potential effect was not confirmed in the literature in both, healthy adults and adults with FF [13–16]. Previous research examined the effects of FO treatment versus a sham condition (i.e., regular insoles) on walking and running kinematics in healthy adults and adults with FF [13,15–17]. These studies revealed small reductions in peak rear foot eversion (up to 1–3 degrees) in the FO condition [13,15–17]. More research was conducted in healthy adults and children with flexible FF [17–19]. These authors were able to show altered frontal plane moments, particularly at the ankle and knee joints, when walking or running with medially posted FOs [17–19]. In healthy adults, these changes are most likely due to a shift of the center of pressure to the direction of FOs posting [20]. It seems that the lever arm of the ground reaction force (GRF) is directed to the joint center. This has been shown in healthy adults, healthy elderly individuals, and patients with knee osteoarthritis [19,21]. Previous studies reported controversial findings on the effects of FO treatment versus a sham condition (i.e., regular insoles) on lower limb kinematics and GRF characteristics during walking in healthy and individuals with flexible FF. While prior studies observed lower peak fore foot pronation in adults with bilateral fore foot varus abnormality [22], other studies reported alterations primarily at the rear foot rather than in fore foot motion in healthy adults and adults with flexible FF [23,24]. Most studies found small and inconsistent differences in kinematic variables when comparing effects of FO treatment versus a sham condition in healthy adults [13,15–17]. Moreover, in a cross-sectional approach, lower (5–20%) peak vertical GRFs were reported when wearing FOs compared to sham in healthy adult men [13]. However, the observed effect was considered insufficient to prevent injuries in healthy adult men [25]. Finally, Sloss (2001) reported higher vertical GRFs (3–5%) at initial peak when wearing FOs versus a sham condition in adults with flexible FF [26]. Due to the inconclusive evidence on the effects of FO treatment versus a sham condition on lower limb kinematics and kinetics [4–6,13,25,26], studies are needed that particularly evaluate the long-term use of FOs on lower limb kinematics and kinetics.

If patients use FOs over longer time periods, they have to get familiarized with FOs during therapy [13]. In this regard, Moison and Cantin [13] examined the effects of a 1 month FO treatment on lower limb muscle activation (e.g., gluteus medius, vastus lateralis, medial and lateral gastrocnemius) in healthy adults aged 19–25 years. These authors reported a decrease in peak amplitude and mean activity of the m. peroneus longus during the combined midstance/terminal stance phase of walking. During the ground contact phase, FOs resulted in a decrease in peak amplitude and mean activity of the m. tibialis anterior. However, this study is limited in as much as the authors did not assess the effects of FOs on kinematics and kinetics during walking. In addition, this study was conducted with adults which is why these findings cannot directly be translated to youth due to growth and maturational processes.

Therefore, it is timely and imperative to study the long-term effects of wearing FOs on walking kinematics and kinetics in children with flexible FF [14]. Thus, the objective of this study was to evaluate the effects of a four months arch support FO treatment versus a sham condition on lower extremity kinematics and kinetics during walking in children with flexible FF. With reference to the relevant literature on the effects of FO treatment on lower limb kinematics and kinetics in children and adults [15, 16], we hypothesized that the long-term treatment of arch support FOs results in improved three-dimensional kinematics (e.g., peak ankle eversion, peak knee internal rotation) and kinetics (e.g., first vertical GRF peak and lateral GRF peak) in boys with flexible FF.

## Materials and methods

### Study design

The study was designed as a double-blind randomized controlled trial (i.e., participants, examiners). The block randomization method was used to allocate study participants into experimental groups. Of note, participants were blinded to group allocation. One examiner determined whether a participant was eligible for inclusion in the trial, while the other carried out gait analyses of the eligible participants. Both examiners were unaware of group allocation. Another naïve examiner controlled the allocation of each participant and was responsible for delivering the treatment to both groups. Study participants with FF and their parents were unaware of the allocation process.

### Participants

We used the freeware tool G*Power (http://www.gpower.hhu.de/) to calculate a one-sided *a* priori power analysis with the F test family (ANOVA repeated measures within-between interaction) and the respective statistical test based on a related study that examined walking kinetics in adults with FF [15]. The power analysis was computed with an assumed Type I error of 0.05, a Type II error rate of 0.20 (80% statistical power), 2 tests (pre, post), a correlation coefficient of 0.5 between observations, and an effect size of 0.80 (i.e., interaction effects) for walking kinetics (i.e., peak vertical GRF). The analysis revealed that 30 subjects would be sufficient to observe large Group by Time interactions. According to Cohen, a large effect size (>0.8) implies that the means of the two experimental groups differ by 0.8 standard deviations [27]. In clinical terms, 30 participants corresponds to a rather small sample size, particularly in regards of epidemiological studies. However, this study did not examine injury rates over time as a primary endpoint. We were interested in biomechanical gait parameters which is why the sample size is sufficiently high. Therefore, 30 boys with flexible FF volunteered to participate in this study which lasted from September 2017 to January 2018. The participants were recruited from orthopaedic specialists in the local community. Previous studies identified differences in biomechanical walking characteristics between females and males. More specifically, it has been shown that women compared with men walked with greater transverse plane pelvis and torso rotation, greater hip ab/adduction, hip rotation, knee abduction as well as greater ankle flexion/extension [17–19]. With reference to previous studies and due to the reported sex differences in biomechanical walking characteristics, boys were recruited in this study. Written informed consent was obtained from the parents or legal representatives of all participants. Ethics approval was obtained from the Research Ethics Board of the Medical Sciences, University of Ardabil (IR-ARUMS-REC-1396-90), and registered with the Iranian Registry of Clinical Trials (IRCT2017082235517N1; URL: http://www.irct.ir/user/trial/26811/view).

**Fig 1:**
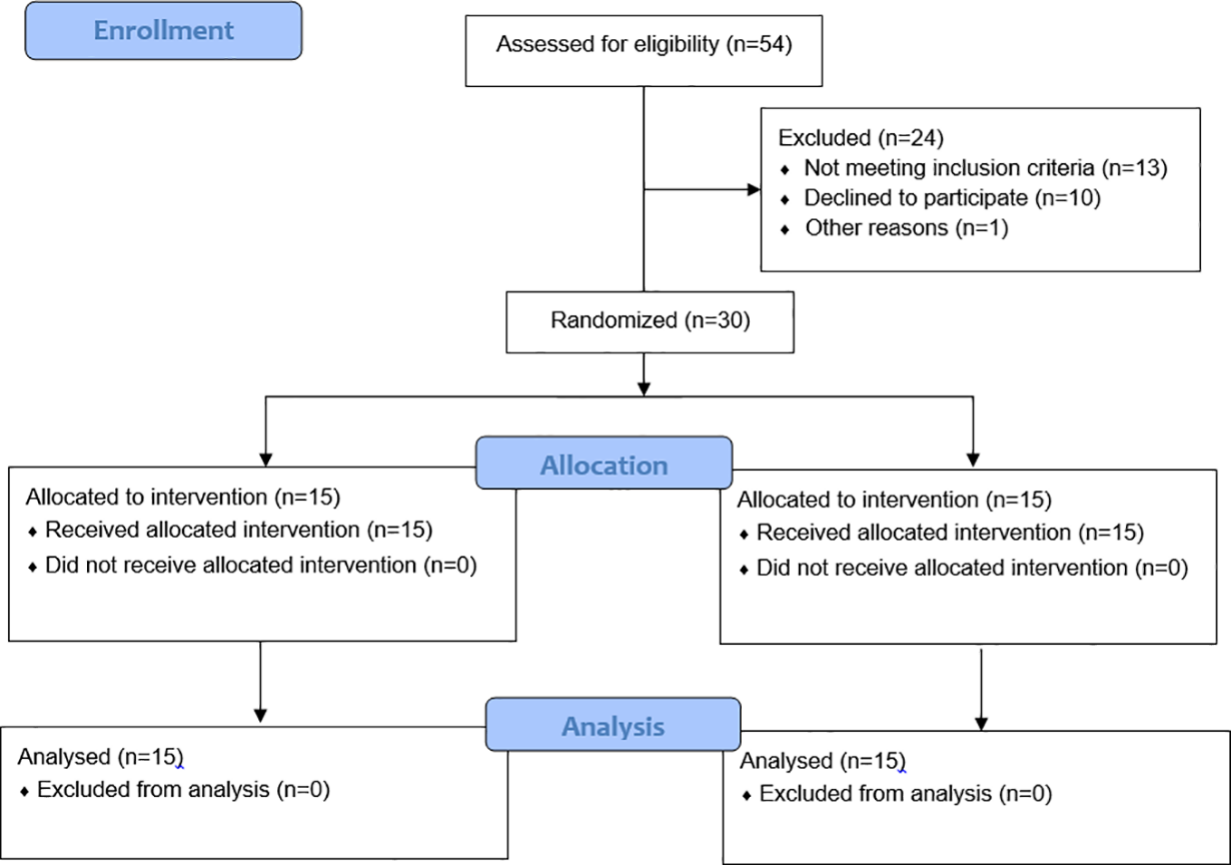
Flow diagram of the randomized controlled trial.

The application of FOs represents an established therapeutic means that has also been recommended as part of the ‘Best Practice Guide’ for management of lower limb injuries [20, 21]. Clinical trials demonstrated that FOs are safe to use and superior to wait-and-see approaches [22] and flat insoles [23]. Due to the established efficacy of FOs, we used FO treatment in this study. To determine whether participants suffered from flexible FF, navicular drop, arch height index (AHI), and resting calcaneal stance position were tested [24–26]. Previous studies demonstrated acceptable reliability for our methodological approach to determine flat feet (ICC>0.97 for all) [28, 29]. More specifically, all clinical measures (i.e., navicular drop and AHI) demonstrated significant (p<0.01) and high associations (r = 0.71–0.91) with the gold standard (X-rays) [28, 29]. The measure of AHI is unitless and was defined as the ratio of dorsal height at 50% of total foot length, divided by the foot length from the back of the heel to the head of the first metatarsal, defined as the truncated foot length [16]. Standing AHI was obtained with the participant standing with equally distributed weight on both feet. The flexible FF samples included participants whose feet had a >10 mm navicular drop [24], >4° eversion in calcaneal stance position [25], and arch height index less than 0.31 [30]. Participants were eligible for inclusion if they were boys aged 8–12 years. Individuals were excluded from study participation if they had a history of bone fractures, signs of functional lower limb instability, ligament injury, reconstruction of ligaments, neuromuscular dysfunction, dysfunction of lower limb muscles, leg length differences larger than 1 cm, and a history of lower extremity trauma or surgery. All participants were right foot dominant which was determined by means of a kicking ball test [31]. Thereafter, participants were randomly allocated to an experimental (EG) or a control group (CG) (Fig 1). The EG (n = 15) received an insole with corrective elements (i.e., arch support FOs) and used it for four months during everyday activities. The CG (n = 15) was equipped with an insole without corrective elements (i.e., sham condition) and applied it for the same period of time.

During the randomization process, a set of sealed, opaque envelopes was used to ensure the concealment of allocation. Each envelop contained a card indicating to which group the subject was allocated. Neither the participating child nor the parents were aware of the respective group they were allocated to. A naïve person that did not have a role in this study was responsible for the allocation process.

### Apparatus

Kinematic data were collected at a sampling rate of 100 Hz using a six-camera VICON motion capture system (Vicon system, Oxford Metrics, Oxford, UK) and 16 spherical reflective markers with a diameter of 15 mm. The size of the cubic calibration volume was 4.0 m (length) × 2.0 m (width) × 2.0 m (height) that was located at the middle of a 15 m walkway. The Plug–in-Gait marker set was used to identify the bilateral pelvis, thighs, legs, and feet provided in Nexus software. The Plug-in-Gait marker set is a commonly used variant of the conventional gait model which is part of the Vicon/Nexus software package. Previous studies reported acceptable reliability [32, 33]. Two force platforms (Kistler, type 9281, Kistler Instrument AG, Winterthur, Switzerland) were used to record GRF data from each leg at a sampling rate of 1000 Hz. Kinematic and kinetic data were synchronized using the Vicon system.

### Gait analysis

Before the tests started, participants’ anthropometrics were assessed from selected anatomical landmarks and entered into the Nexus software. Thereafter, reflective markers were attached bilaterally to the following anatomical landmarks of our participants: anterior superior iliac spine, posterior superior iliac spine, lateral mid-thigh, lateral femoral epicondyle, mid shank, lateral malleoli, heel and toe between second and third metatarsal heads. The bilateral heels and toes markers could not be attached directly to the participants’ skin during shod walking. Therefore, these markers were attached to appropriate positions on the shoe. Afterwards, each subject performed several walking trials to be familiarized with the experimental test setup. One static trial during standing and one dynamic trial during walking at preferred gait speed were collected and processed to evaluate the knee ab-/adduction kinematic profile. If the knee abduction/adduction profile exceeded a range of motion of 10° and exhibited cross-talk with knee flexion/extension, the thigh marker was adjusted and a new static trial was collected. This final static trial was used for all analyzed walking trials [32]. During pre and post gait analyses, six successful trials were recorded at preferred gait speed. A trial was accepted if subjects showed a normal stride pattern (i.e., without any loss of balance and without falling during walking). Trials were excluded if subjects hit the edge of the force plate during heel strike or if they completely missed the force plate. Gait analyses were performed during both tests (pre, post) by the same experienced examiner who has been working for more than 10 years in our gait analysis laboratory.

GRF data were filtered using a fourth-order low-pass Butterworth filter with a 20 Hz cutoff frequency. Kinematic data were filtered using a zero lag fourth order Butterworth filter with a 6 Hz cutoff frequency. Kinematic data were calculated during the stance phase of walking which was defined as the interval from ground contact (vertical GRF>10 N) to toe off (vertical GRF<10N) [34]. Data were normalized to the stance phase (i.e., heel contact to toe-off = 100%) using spline interpolation with MATLAB. Plug-in-Gait lower body model (Vicon Motion Systems) [35] was utilized for data processing, and graphical reports were created in Polygon Authoring Tool (PAT). Data were exported from PAT to a spreadsheet for patterns, ranges of motion and other specific data point calculations. The dependent variables that were selected to be entered into the statistical analysis were based on previous studies on gait patterns of individuals with flexible FF [3, 36–42].

The kinematic variables were i) maximum ankle plantarflexion angle during the loading response, ii) maximum ankle dorsiflexion angle during mid-stance, iii) maximum ankle plantarflexion angle at toe-off, iv) maximum ankle inversion angle, v) maximum frontal angle of the ankle during push-off (maximum eversion in some participants and minimum inversion in others), vi) maximum ankle external rotation angle, vii) maximum ankle internal rotation angle, viii) maximum knee flexion angle during loading response, ix) maximum knee extension angle during mid-stance, x) maximum knee flexion angle at toe-off, xi) minimum knee abduction angle, xii) maximum knee abduction angle, xiii) maximum knee external rotation angle, xiv) maximum knee internal rotation angle, xv) maximum hip extension angle, xvi) maximum hip adduction angle, xvii) maximum hip external rotation angle. As a convention for the applied kinematic variables, positive signs are used for ankle dorsiflexion, ankle inversion, ankle internal rotation, hip and knee flexion, hip and knee adduction, as well as hip and knee internal rotation; negative signs are used for ankle plantarflexion, ankle eversion, ankle external rotation, hip and knee extension, hip and knee abduction, as well as hip and knee external rotation. The GRF values were recorded along the vertical (z), the anterior-posterior (y), and the medio-lateral (x) directions. The vertical GRF bimodal curve in normal walking contains two peaks including the first peak on heel contact (Fz_HC_) and the second peak on the push-off phase (Fz_PO_). Additionally, on the anterior-posterior curve, two peaks were resolved for analysis, as the braking reaction force (Fy_HC_) and propulsion (Fy_PO_) forces. Moreover, on the medio-lateral curve, three peaks were analyzed corresponding to the positive peak which occurred at the heel contact (Fx_HC_), followed the two consecutive negative peaks at the mid stance (Fx_MS_) and the push-off phase (Fx_PO_) [43].

### Application of orthoses

**Fig 2:**
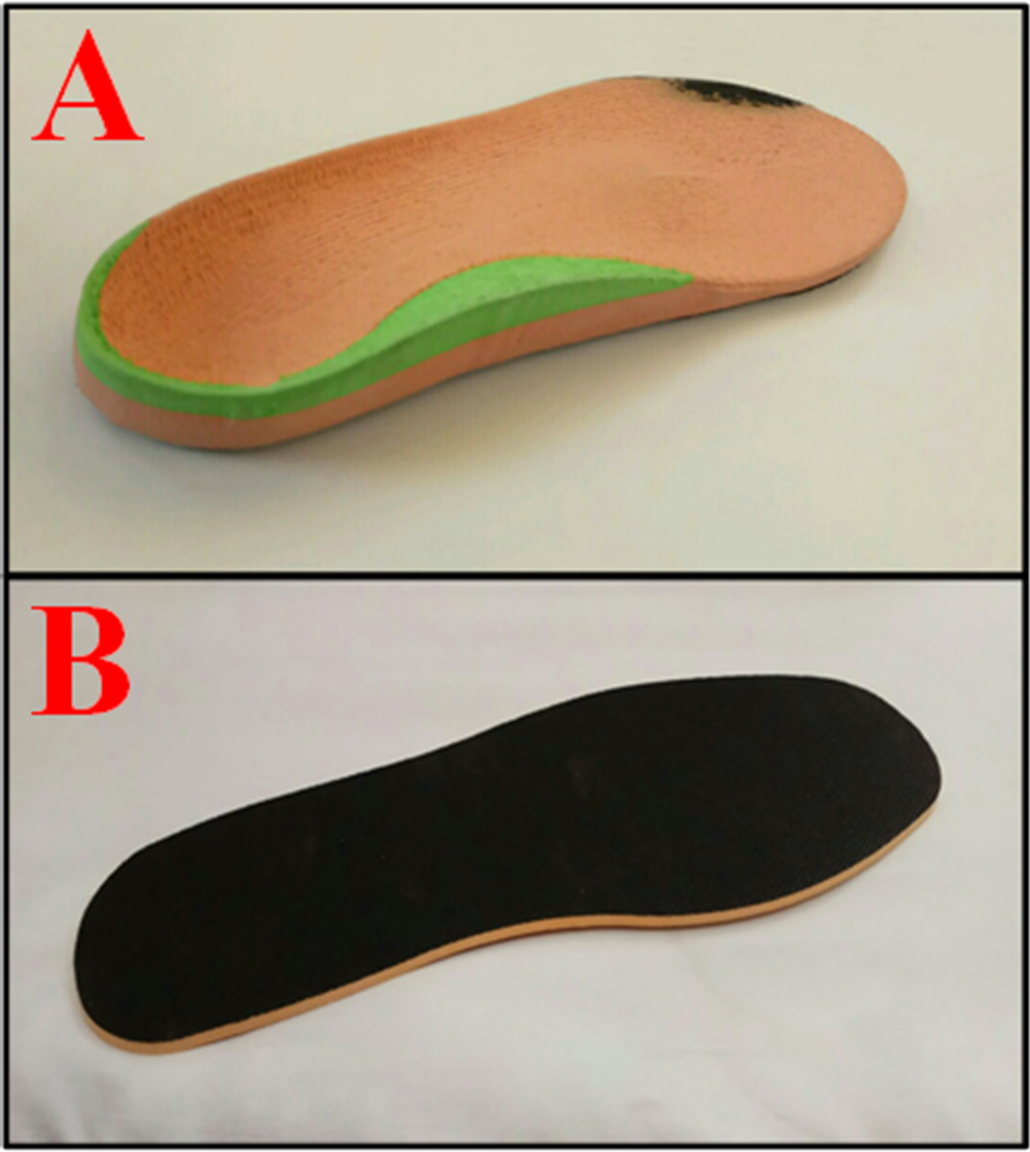
Fig (A) illustrates an arch support foot orthoses that was applied in the experimental group. Fig (B) shows a flat, 2-mm-thick insole that was used in the control group as sham condition.

It has previously been demonstrated that shoe type affects the walking pattern in children [44, 45]. Therefore, all study participants were equipped with the same neutral shoe model (New Balance 759, USA) with no intrinsic correction. Custom-made medial arch support FOs made out of ethylene vinyl acetate and microcellular rubber were used to produce the negative impression of the foot held in subtalar joint neutral position in EG (Fig 2A). In the EG, FOs peak longitudinal height of the mid-foot arch was 25 mm. For the CG, flat 2-mm-thick insoles were used as a sham condition. The insoles were made of polyester resin (Fig 2B).

### Test protocol

During baseline tests, data were collected while walking with standard shoes and without FOs. After the pre-tests, each study participant wore FOs (EG) and insoles (CG) for 4 months during everyday activities. During the intervention period, participants from both groups were instructed to progressively increase the application time of their FOs (EG) and insoles (CG). On the first day, application time was restricted to one hour to allow familiarization. On every following day, application time was increased by one hour until participants finally wore the FOs (EG) / insoles (CG) for the full day [13]. On every day during the 4 months intervention period, FO wearing time was registered in a log by the parents of the study participants [13]. Of note, post-tests were conducted at the same time of day and in the similar test sequence as the baseline tests.

Furthermore, no significant differences in leg length adjusted walking speed were detected from pre-to-post in both groups (CG pre: 2.44±0.38 m/m/s, post: 2.43±0.34 m/m/s; EG pre: 2.43±0.37 m/m/s, post: 2.42±0.35 m/m/s; all p>0.05).

### Statistical analyses

Data are presented as group mean values and standard deviations. After normal distribution was examined and confirmed using the Shapiro-Wilk-Test, an independent samples t-test was calculated to determine baseline between-group differences. The long-term effects of FOs and insoles on lower limb kinetics and kinematics during walking in children were analyzed in a randomized controlled trial. For this purpose, a separate 2 (Groups: EG, CG) × 2 (Time: pre, post) ANOVA with repeated measures on “Time” was computed. Group-specific and Bonferroni corrected pre-post changes were calculated with the help of paired sample t-tests. Additionally, effect sizes were determined by converting partial eta-squared (η^2^_p_) to Cohen’s d. According to Cohen [27], *d* < 0.50 indicate small effects, 0.50 ≤ *d<* 0.80 indicate medium effects, and *d* ≥ 0.80 indicate large effects. The significance level was set at *p* < 0.05. All analyses were performed using the Statistical Package for Social Sciences (SPSS) version 22.0. It has previously been shown [46] that some adult participants respond to the use of orthoses and others not. We expected that this would be the same in children. Therefore, our study sample was divided in responders and non-responders according to each participant's change in ankle eversion which is the most important injury risk factor in patients with FF.

## Results

Participant characteristics are illustrated in Table 1. No significant between-group differences were found at baseline for all examined variables (Table 1).

**Table 1:** Group-specific baseline values of all reported anthropometric, kinematic and kinetic outcome variables.

Parameter	EG	CG	p-value
Age(years)	10.5 ± 1.4	10.4 ± 1.5	0.952
Body height(cm)	142.4 ± 5.7	141.2 ± 6.1	0.910
Body mass(kg)	48.1 ± 9.1	48.2 ± 9.4	0.964
BMI(kg/m^2^)	20.0±4.0	20.1±4.2	0.924
Navicular drop(mm)	13.0±2.1	13.1±1.9	0.955
AHI	0.18±0.07	0.18±0.06	0.995
Calcaneal eversion(degree)	7.2±1.1	7.1±0.9	0.898
Kinematics			
A1	-0.8±2.4	-0.7±2.8	0.902
A2	22.0±3.5	22.7±3.5	0.567
A3	-3.4±2.9	-3.6±3.3	0.851
A4	4.9±0.8	4.9±1.0	0.820
A5	-0.3±1.1	-0.1±0.8	0.548
A6	-9.8±1.1	-9.8±7.7	0.781
A7	5.2±0.8	5.0±0.5	0.373
K1	14.2±3.2	13.6±2.7	0.574
K2	1.9±3.1	2.1±3.1	0.902
K3	60.2±4.6	61.3±6.7	0.615
K4	-7.4±0.6	-7.2±1.0	0.602
K5	-15.1±0.8	-14.9±1.6	0.664
K6	-12.7±0.9	-12.8±1.1	0.709
K7	8.1±2.5	8.1±2.9	0.991
H1	-10.0±3.5	-9.7±3.5	0.852
H2	3.6±1.0	3.5±1.2	0.914
H3	-18.6±1.0	-18.7±1.5	0.929
Kinetics			
Fz_HC_	115.4±10.4	113.9±10.1	0.707
Fz_PO_	108.5±9.3	113.1±6.1	0.124
Fy_HC_	-30.9±9.9	-28.5±6.8	0.449
Fy_PO_	33.6±8.8	35.6±5.6	0.474
Fx_HC_	7.6±4.3	10.2±6.1	0.190
Fx_MS_	-11.0±2.7	-11.2±2.5	0.872
Fx_PO_	-11.2±3.1	-11.6±2.8	0.688

Note: BMI, Body mass index; AHI, Arch height index; A1, maximum ankle plantarflexion angle during loading response; A2, maximum ankle dorsiflexion angle during mid-stance; A3, maximum ankle plantarflexion angle at toe-off; A4, maximum ankle inversion angle; A5, maximum frontal angle of the ankle during push-off; A6, maximum ankle external rotation angle; A7, maximum ankle internal rotation angle; K1, maximum knee flexion angle during loading response; K2, maximum knee extension angle during mid-stance; K3, maximum knee flexion angle at toe-off; K4, minimum knee abduction angle; K5, maximum knee abduction angle; K6, maximum knee external rotation angle; K7, maximum knee internal rotation angle; H1, maximum hip extension angle; H2, maximum hip adduction angle; H3, maximum hip external rotation angle; Fz_HC_; First peak vertical ground reaction force at heel contact; Fz_PO_, Second peak vertical ground reaction force at push-off; Fy_HC_, Braking reaction force; Fy_PO_, Propulsion force; Fx_HC_, Peak medial ground reaction force at heel contact; Fx_MS_ and Fx_PO_ are two consecutive negative peaks at the mid stance and the push-off phase, respectively. p value from independent samples t-test, SD = standard deviation.

Over the four months intervention period, the average daily wearing time for orthoses and insoles amounted to 6.8±3.8 hours for the EG and 7.0±3.7 hours for the CG. The between-group difference for average daily wearing time was not statistically significant (p > 0.05). Participants from both groups did not report any adverse events from wearing FOs or insoles over the course of the study.

### Kinematic analyses

Table 2 describes pre and post data for all examined kinematic variables. The statistical analyses indicated significant main effects of “Time” for maximum ankle internal rotation angle, maximum knee external rotation angle, and maximum knee internal rotation angle (Table 2). The statistical analysis yielded significant group by time interactions for maximum frontal angle of the ankle during push-off, maximum ankle internal rotation angle, minimum knee abduction angle, maximum knee abduction angle, maximum knee external rotation angle, maximum knee internal rotation angle, maximum hip extension angle, and maximum hip external rotation angle (Table 2).

**Table 2:** The long-term effects of foot orthoses on lower limb kinematics during walking.

Variable	EG (n = 15)						CG (n = 15)						*p*-value (effect size d)		
	Pre-Test		Post-Test		Δ (degree)	95%CI	Pre-Test		Post-Test		Δ (degree)	95% CI	Main effect: Time	Main effect: Group	Interaction: Time × Group
	M	SD	M	SD			M	SD	M	SD					
A1	-0.8	2.4	-2.1	2.1	1.3	0.3,2.4	-0.7	2.8	-1.3	2.7	0.6	-1.0,2.1	0.118 (0.87)	0.089 (0.97)	0.201 (0.70)
A2	22.0	3.5	22.3	2.8	0.3	-2.5,1.9	22.7	3.5	22.2	4	-0.5	-1.6,2.6	0.882 (0.00)	0.600 (0.29)	0.591 (0.29)
A3	-3.4	2.9	-2.2	4.3	-1.2	-3.4,1.0	-3.6	3.3	-4.7	3.1	1.1	-1.4,3.6	0.933 (0.00)	0.039 (1.22)	0.107 (0.91)
A4	4.9	0.8	4.7	0.8	0.7	-0.2,0.7	4.9	1.0	5	0.8	0.1	-0.8,0.6	0.848 (0.00)	0.712 (0.20)	0.336 (0.55)
A5	-0.3	1.1	0.4	1.2	0.7	-1.4,-0.1	-0.1	0.8	-0.2	0.8	0.1	-0.5,0.7	0.160 (0.81)	0.494 (0.35)	**0.048** (1.16)
A6	-9.8	1.1	-10	0.3	0.2	-0.4–0.9	-9.8	0.7	-9.6	1.7	-0.2	-1.3,0.7	0.997 (0.00)	0.612 (0.29)	0.278 (0.59)
A7	5.2	0.8	3.3	1.2	-1.9	1.0,2.7	5.0	0.5	5.3	1.2	0.3	-0.9,0.2	**0.006** (1.70)	**0.002** (2.00)	**< .001** (2.45)
K1	14.2	3.2	16.2	1.1	2.0	-3.5,-0.5	13.6	2.7	14	1.9	0.4	-2.5,1.6	0.119 (0.87)	**0.005** (1.81)	0.078 (1.03)
K2	1.9	3.1	1.5	0.9	-0.4	-0.9,1.8	2.1	3.1	1.4	2.5	-0.7	-1.3,2.7	0.471 (0.41)	0.972 (0.00)	0.740 (0.20)
K3	60.2	4.6	60.3	2.3	0.1	-2.3,2.1	61.3	6.7	60.6	7.8	-0.7	-5.9,7.3	0.867 (0.00)	0.437 (0.41)	0.780 (0.20)
K4	-7.4	0.6	-6.5	0.3	-0.9	-1.1,0.5	-7.2	1.0	-7.5	1.1	0.3	-0.3.0.8	0.093 (0.97)	**0.031** (1.28)	**0.001** (2.26)
K5	-15.1	0.8	-13.8	0.8	-1.3	-1.9,0.7	-14.9	1.6	-15.3	1.8	0.4	-0.5,1.3	0.104 (0.94)	0.062 (1.09)	**0.003** (1.96)
K6	-12.7	0.9	-10.7	0.7	-2.0	-2.5,-1.4	-12.8	1.1	-12.2	1.3	-0.6	-0.5,1.3	**< .001**(2.85)	**0.020** (1.40)	**0.008** (1.67)
K7	8.1	2.5	4.8	0.6	-3.3	1.8,4.8	8.1	2.9	7.8	2.1	-0.3	-1.4,2.1	**0.005** (1.81)	0.017 (1.47)	**0.016** (1.47)
H1	-10.0	3.5	-13.2	5.2	3.2	1.1,5.3	-9.7	3.5	-9.5	3.3	-0.2	-2.8,2.4	0.103 (1.81)	0.106 (0.94)	**0.028** (1.31)
H2	3.6	1.0	3.1	2	-0.5	-0.7,1.6	3.5	1.2	3.9	1.1	0.4	-1.0,0.3	0.884 (0.00)	0.183 (0.74)	0.215 (0.70)
H3	-18.6	1.0	-16.7	0.6	-1.9	-2.7,1.0	-18.7	1.5	-19	3.4	0.3	-1.4,2.0	0.059 (1.09)	0.054 (1.12)	**0.042** (1.18)

Note. M = mean; SD = standard deviation; Δ (degree) = the value of progress calculated as: (post-test–pre-test).; A1, maximum ankle plantarflexion angle during loading response; A2, maximum ankle dorsiflexion angle during mid-stance; A3, maximum ankle plantarflexion angle at toe-off; A4, maximum ankle inversion angle; A5, maximum frontal angle of the ankle during push-off; A6, maximum ankle external rotation angle; A7, maximum ankle internal rotation angle; K1, maximum knee flexion angle during loading response; K2, maximum knee extension angle during mid-stance; K3, maximum knee flexion angle at toe-off; K4, minimum knee abduction angle; K5, maximum knee abduction angle; K6, maximum knee external rotation angle; K7, maximum knee internal rotation angle; H1, maximum hip extension angle; H2, maximum hip adduction angle; H3, maximum hip external rotation angle. Significant p values were highlighted in bold.

**Fig 3:**
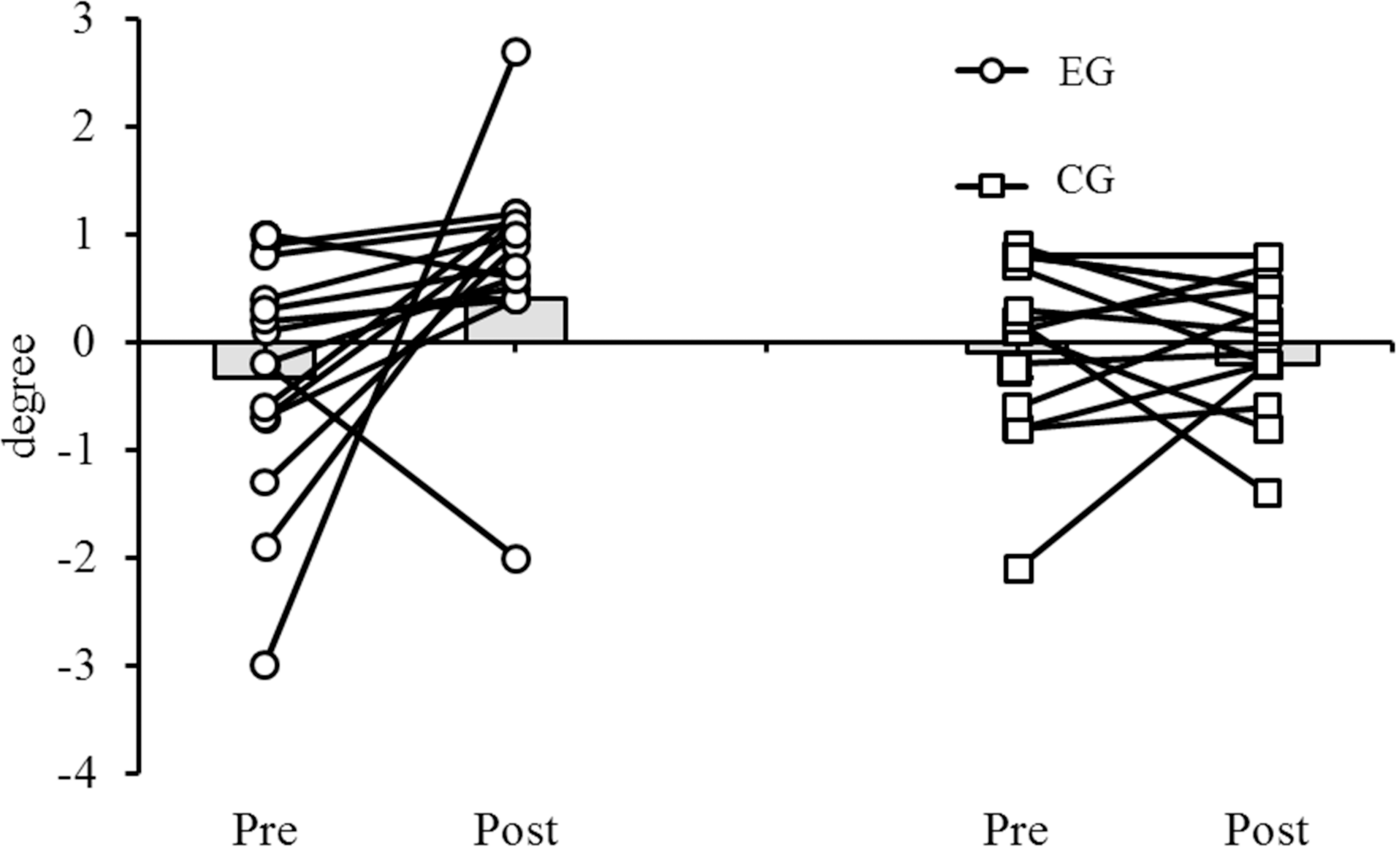
Maximum frontal angle (degree) of the ankle for each subject during the push-off phase of walking (A5). Positive values are related to inversion and negative values to eversion movements. Group-specific mean values are indicated by grey bars.

**Fig 4:**
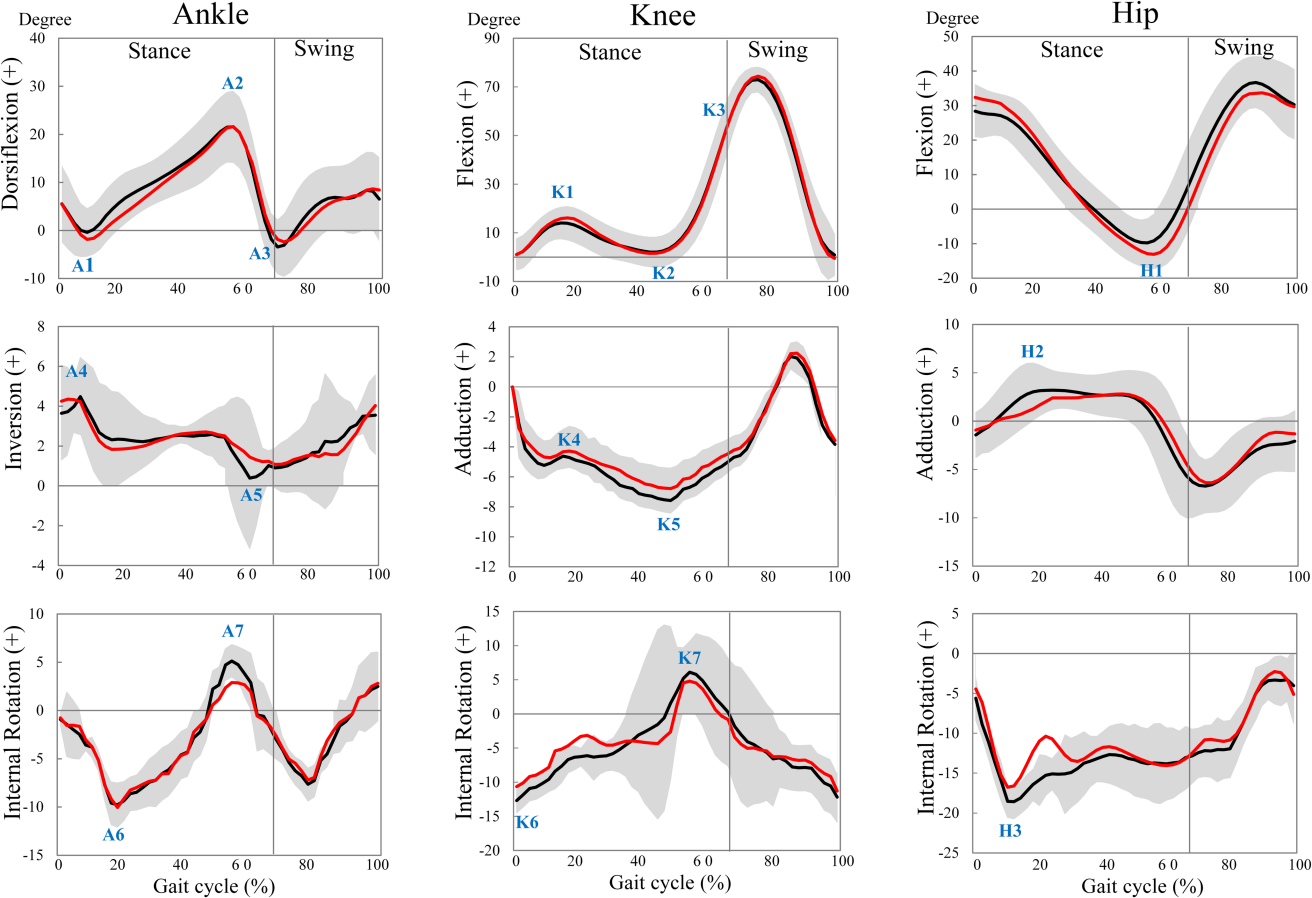
Ensemble average kinematics for all 15 subjects of the experimental group. The black and red curves represent the values for the pre- post-test condition, respectively. Grey shading illustrates the 95% confidence interval for the pre-test condition. For clarity, errors are not shown for the post-test condition.

In the EG but not in the CG, significant decreases were found from pre to post for maximum ankle internal rotation angle (p<0.001; d = 1.9; 95% CI: 1, 2.8), minimum knee abduction angle (p<0.001; d = 2.0; 95% CI: -1.1, -0.5), maximum knee abduction angle (p<0.001; d = 1.6; 95% CI: -2.0, -0.7), maximum knee external rotation angle (p<0.001; d = 2.5; 95% CI: -2.6, -1.5), maximum knee internal rotation angle (p<0.001; d = 2.1; 95% CI: 1.8, 4.8), and maximum hip external rotation angle (p<0.001; d = 2.4; 95% CI: -2.7, -1.0). In addition, our post-hoc analyses revealed significant pre-post increases for the EG in maximum ankle plantarflexion angle during the loading response (p = 0.015; d = 0.6; 95% CI: 0.3, 2.4), maximum frontal angle of the ankle during push-off (p = 0.026; d = 0.6; 95% CI: -1.4, -0.1), maximum knee flexion angle during loading response (p = 0.012; d = 0.9; 95% CI: -3.5, -0.5), and maximum hip extension angle (p = 0.006; d = 0.7; 95% CI: 1.1, 5.3). These changes are presented in Figs 3 and 4 which illustrates the patterns of lower limb joint angles (i.e., hip, knee, and ankle) during a walking cycle.

Our sub-analyses on responders / non-responders following FOs treatment revealed that at baseline 53% of EG boys with flat-arched feet showed an ankle eversion during the push-off phase of walking. After the 4 months intervention period, 85% of them showed an improvement towards inversion (Figs 3 and 4).

### GRF analyses

Table 3 illustrates pre and post data for all examined GRF measures. Our statistical analyses indicated a significant main effect of “Time” for maximum vertical GRF (Table 3). Moreover, statistically significant group by time interactions were detected for maximum posterior, and vertical GRF (Table 3).

**Table 3:** The long-term effects of foot orthoses on ground reaction forces during walking.

Variable	EG (n = 15)							CG (n = 15)								*p*-value (effect size d)		
	Pre-Test		Post-Test			Δ (%)	95%CI	Pre-Test		Post-Test			Δ (%)		95%CI	Main effect: Time	Main effect: Group	Interaction: Time × Group
	M	SD		M	SD			M	SD	M	SD							
Fz_HC_	115.4	10.4	106.2		6.6	-7	1.9,16.3	113.9	10.1	113.7		11.4		0	-2.9,3.3	**0.036** (1.25)	0.380 (0.51)	**0.017** (1.43)
Fz_PO_	108.5	9.3	109.6		6.2	1	-7.7,5.5	113.1	6.1	112.3		7.3		0	-1.8,3.4	0.909 (0.00)	**0.045** (1.18)	0.623 (0.29)
Fy_HC_	-30.9	9.9	-22.9		5.2	-26	14.0,-2.0	-28.5	6.8	-29.8		7.7		4	-3.1,5.6	0.085 (1.00)	0.267 (0.63)	**0.012** (1.53)
Fy_PO_	33.6	8.8	34.3		9.6	2	-9.3,7.8	35.6	5.6	36		8.7		1	-3.8,2.9	0.786 (0.20)	0.284 (0.59)	0.940 (0.00)
Fx_HC_	7.6	4.3	5.7		4.2	-25	-1.3,5.2	10.2	6.1	9.7		5.8		-4	-3.6,4.7	0.235 (0.63)	**0.039** (1.22)	0.631 (0.29)
Fx_MS_	-11.0	2.7	-11.8		2.7	6	-0.5,2.0	-11.2	2.5	-10.7		3.1		-3	-3.0,2.1	0.822 (0.00)	0.583 (0.29)	0.436 (0.41)
Fx_PO_	-11.2	3.1	-11.1		1.9	-2	-1.8,1.5	-11.6	2.8	-11.1		3.1		-4	-3.2,2.2	0.626 (0.29)	0.652 (0.29)	0.861 (0.00)

Note. M = mean; SD = standard deviation; Δ (%) = the percent of progress calculated as: ((post-test–pre-test)/ pre-test) × 100; Fz_HC_; First peak vertical ground reaction force at heel contact; Fz_PO_, Second peak vertical ground reaction force at push-off; Fy_HC_, Braking reaction force; Fy_PO_, Propulsion force; Fx_HC_, Peak medial ground reaction force at heel contact; Fx_MS_ and Fx_PO_ are two consecutive negative peaks at the mid stance and the push-off phase, respectively. Significant p values were highlighted in bold.

**Fig 5:**
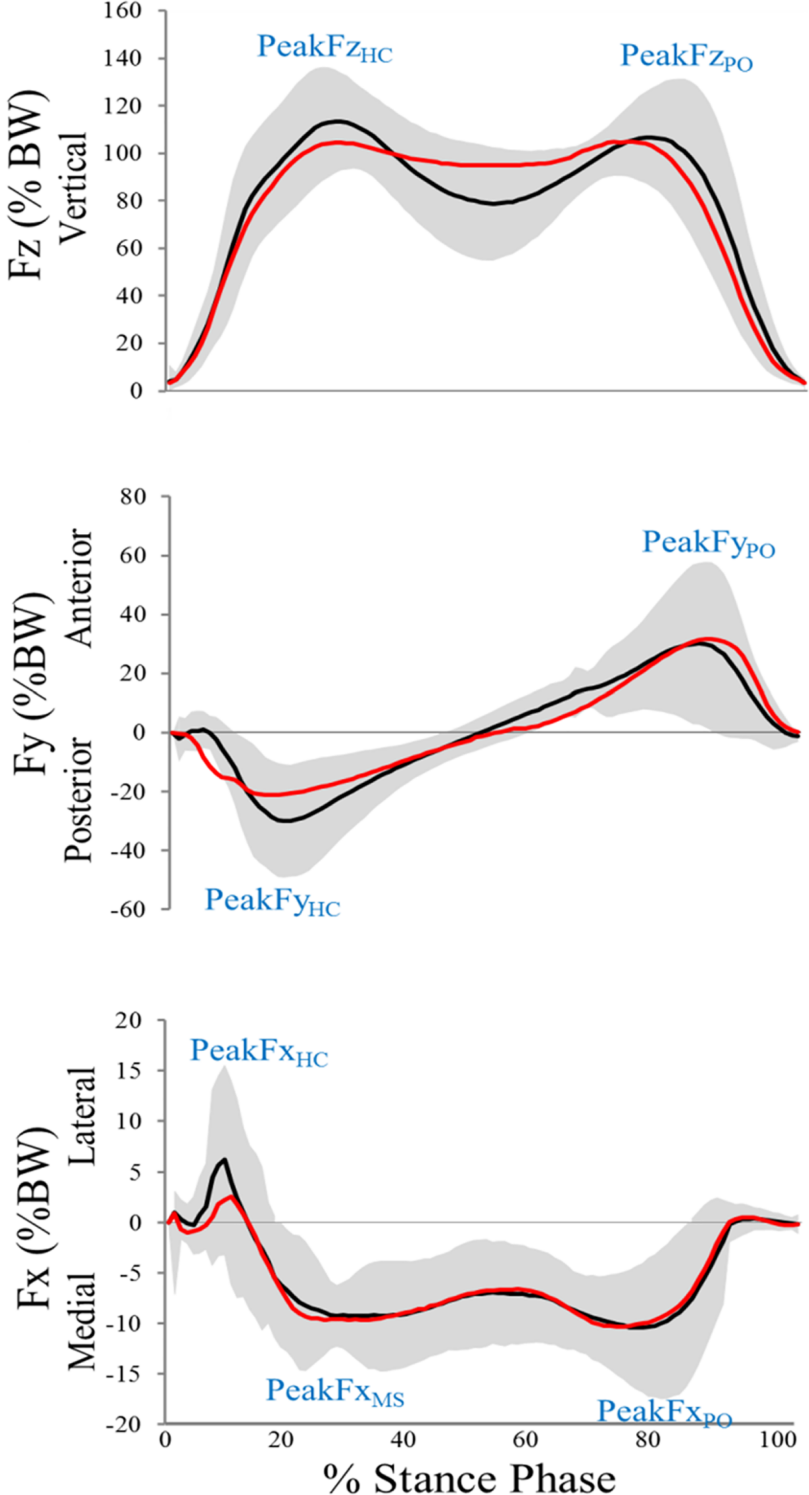
Ensemble average of three-dimensional GRF values for all 15 subjects of the experimental group. The black and red curves represent the values for the pre- post-test condition, respectively. Grey shading illustrates 95% confidence interval for the pre-test condition. For clarity, errors are not shown for the post-test condition.

In terms of the EG, significant decreases were found from pre to post for maximum vertical (p = 0.017; d = 1.08; 95% CI: 1.9, 16.3) and posterior (p = 0.012; d = 0.3; 95% CI: 2, 14) GRF values at heel strike. These changes are presented in Fig 5 which illustrates GRF patterns (i.e., vertical, anterior-posterior, medio-lateral) during the stance phase of walking.

## Discussion

Clinical trials demonstrate that FOs are safe to use and superior to wait-and-see-approaches [22] and flat insoles [23]. This randomized controlled trial examined for the first time the long-term effects of wearing FOs versus regular insoles (i.e., sham condition) on walking kinematics and kinetics in boys with flexible FF. As a main finding, we detected that the long-term application of FOs resulted in significant improvements in walking kinematics (e.g., maximum ankle internal rotation angle, maximum knee external rotation angle, and maximum knee internal rotation angle) and kinetics (e.g., maximum posterior and vertical GRF) in boys with flexible FF compared to a sham condition. We conclude that FO treatment is feasible and effective to improve lower limb alignment in boys aged 8 to 12 years.

Given that hardly any research has been conducted on the effects of FOs application on lower limb kinematics and kinetics during walking in children, we interpreted our findings by taking adult studies into consideration. The observed results are in accordance with findings from cross-sectional studies that examined the effects of FO treatment versus a sham condition (i.e., regular insoles) and with longitudinal studies that looked at the long-term effects of FO treatment on walking mechanics. In terms of the acute effects of FO treatment versus a sham condition, Tang et al. (2015) reported that the application of orthoses produced lower frontal plane rearfoot motion and plantar pressure in adult patients with flexible FF [47]. Furthermore, Jafarnezhadgero et al. (2017) observed significantly lower peak ankle evertor moment due to FOs treatment compared to a sham condition in boys with flexible FF [48]. A previously conducted study that examined the acute effects of FO treatment versus a sham condition in young men with pronated feet did not show any significant effects on hind-foot eversion during walking and running [49]. Of note, excessive foot eversion has previously been identified as a risk factor for lower limb injuries or low back pain in both, adults and children [3, 50–54]. With reference to the above mentioned findings from the literature, we interpret our results regarding the observed reductions in maximum ankle eversion angle as beneficial for boys with FF.

Moreover, our results are in line with the findings from Lucas and Cornwall who reported that the application of an average medial wedge of 5.1 mm induced a functioning windlass mechanism in those adults that did not exhibit a windlass mechanism before treatment [55]. The absence of a functioning windlass mechanism may delay rear-foot supination during the late stance phase of gait and may put excessive stress on the subtalar and mid-tarsal joint which can result in ankle injuries. Furthermore, our results demonstrated a reduction in maximum ankle internal rotation angle after the long-term use of arch support FOs in boys with FF. We additionally found that peak eversion angle did not fully decrease in the EG after the four months intervention period. However, mean excessive ankle eversion significantly improved following FO treatment. In accordance with Nigg (2001) [56], our results demonstrated that there are responders and non-responders to long-term FOs application. Nigg postulated that further research is needed to elucidate why individuals display different foot motion patterns during the gait cycle and why the effects of orthotic devices largely vary between individuals [57].

In a cross-sectional approach, it was shown that FOs compared to a sham condition produced a larger peak knee external rotation angle during the mid-stance phase of walking in healthy women and in women with FF aged 18–50 years [58]. In another cross-sectional study so significant differences were found between FO application and a sham condition on knee kinetics during walking in men aged 18–30 years with pronated feet [49]. However, Jafarnezhadgero et al. (2017) reported lower knee abductor moments in the dominant limb in the FOs condition compared to the sham condition in children [48].

Our results revealed that maximum angle values of the knee abduction, knee external rotation, and knee internal rotation decreased after four months of FO therapy. Chen et al. (2010) did not find any significant differences in peak knee flexion angle in adults (males and females) with FF aged 30–60 years in the FO condition compared to the sham condition [59]. The reported differences in findings between our study and the study of Chen et al. [59] could be due to differences in testing methods and/or investigated study populations. While we applied kinematic analysis during walking using the VICON system, Chen et al. used Eagle digital motion analysis system (Motion Analysis Corporation, Santa Rasa, CA, USA) for kinematic analysis [59]. In addition, we observed boys with FF aged 9–12 years. Chen et al. examined adults with flatfoot (six males and five females) aged 30–60 years [59]. Differences in our results compared to Chen et al. [59] may be due to differences in the applied testing methods or different population groups (e.g., children vs adults). Our study further revealed that the long-term use of FOs resulted in significant increases in maximum knee flexion angle. Previously, it has been reported that children with FF compared to their healthy age-matched peers showed a greater knee abduction angle during the stance phase of walking [38]. The present study revealed how long-term FOs treatment improves (reduced) peak knee abduction angle. Greater knee abduction is consistently reported to form part of the mechanism of non-contact anterior-cruciate ligament injury [60]. Similarly, with respect to patellofemoral pain syndrome, greater knee abduction has been demonstrated to increase contact forces within the lateral patellofemoral joint [61]. From this it follows, that FOs therapy may prevent adverse health-related events in different body regions [59, 62–64]. Also, our results demonstrated higher maximum knee flexion angle during loading response phase after long term use of FOs. Proper function of the neuromuscular system, especially the activation of quadriceps femoris during loading response phase of gait, may protect the musculoskeletal system from potentially adverse impulsive loading [65]. It seems that muscle activity of the quadriceps femoris during knee flexion of the loading response phase contribute to energy absorption (with eccentric contraction) at the initial heel contact to reduce the GRF [66]. Our GRF data (reduction of the first vertical peak) support this finding. Based on our results, a higher maximum knee flexion angle during the loading response phase after long-term use of arch support FOs could be beneficial in boys with FF. Even though there are currently no other studies available that examined the long-term effects of FOs treatment in children, this paper presents information regarding relative reciprocal movement between different joints before and after long term wearing of FOs in boys with flexible FF during gait. This novel data can be used as important information for the design of future studies in this area.

A previous study did not show any significant differences in hip kinetics in the FOs treatment compared to the sham condition in male adults (18–30 years) with pronated feet during walking [49]. Moreover, Chen et al. (2010) could not find any significant differences in hip kinematics and kinetics in the FOs compared to the sham condition in adults (both male and female) with FF aged 30–60 years [59]. However, Jafarnezhadgero et al. (2017) reported lower hip abductor moments and hip flexor moment in male children in the FOs compared to the sham condition [48]. These inconsistencies could be due to methodological differences between the aforementioned studies. Such differences include preferred versus walking speed, and children versus adults or male versus female or mixed participants. Our results revealed that maximum angle value of the hip external rotation decreased after four months of FOs therapy. In contrast, the long term use of FOs resulted in significant increases in maximum angle value of the hip extension. Increased external hip rotation angle (by 6–7°) in children with FF compared to healthy peers was reported in a previous study [38]. In the present study, in the EG, maximum hip external rotation angle were lower during the post compared to the pre-test. Given that our participants were treated with FOs for four months, central adaptation effects (such as motor unit recruitments) may occur that gradually improve muscular function over time. Central adaptation of motor representation after peripheral changes such as amputation, spinal cord injury, or immobilization has been shown in the literature [67, 68]. However, this issue needs future studies to be cleared.

This study exhibits a number of methodological limitations that warrant discussion. Boys aged 8–12 years were enrolled in this study. Therefore, our findings cannot be generalized to younger and older boys due to growth and maturation and to girls due to differences in biomechanical gait characteristics. Also, treatment time was assessed through a log book which is why the recorded mean wearing time per day of 6.8 hours could be biased. Furthermore, markers were placed on the surface of the shoe to detect foot kinematics and ankle joint motions [69–71]. Another potential limitation of this study refers to the use of the Plug-in-Gait model. It has previously been speculated that this model might not be precise and accurate enough to quantify ankle joint kinematics. Therefore, it is recommended that future studies apply 6 degrees of freedom marker set models instead of simple Plug-in-Gait marker sets.

## Conclusions

Our finding demonstrated that the long-term application of FOs resulted in significant improvements in walking kinematics (e.g., maximum ankle internal rotation angle, maximum knee external rotation angle, and maximum knee internal rotation angle) and kinetics (e.g., maximum posterior and vertical GRF) in boys aged 8–12 years with flexible FF compared to a sham condition. The long-term treatment of arch support FOs is effective to improve lower limb kinematics and kinetics during walking in boys with FF. Further studies are warranted to explore the long-term effects of FOs therapy on other walking characteristics such as joint kinetics and muscular activity in children with flexible FF and to examine the effects of sex and maturation. Finally, the effects of a passive stabilizer (orthoses) should be contrasted to an active treatment (e.g., balance training) to elucidate whether passive or active treatment forms are better suited for children with flexible FF.

## Supporting information

S1 File CONSORT 2010 checklist. (DOC)

S2 File Log files of the statistical analyses. (DOCX)

S3 File Our trial study protocol as a supporting information file. (DOCX)
