# Supplementary material for: The long-term use of foot orthoses affects walking kinematics and kinetics of children with flexible flat feet: A randomized controlled trial
Source: PLoS One. 2018 Oct 9;13(10):e0205187. doi: 10.1371/journal.pone.0205187 (PMC6177172; doi:10.1371/journal.pone.0205187)
Supplement: S2 File — (DOCX) [file pone.0205187.s002.docx]

|  |  | Levene's Test for Equality of Variances | | t-test for Equality of Means | | | | | | |
| --- | --- | --- | --- | --- | --- | --- | --- | --- | --- | --- |
|  |  | F | Sig. | t | df | Sig. (2-tailed) | Mean Difference | Std. Error Difference | 95% Confidence Interval of the Difference | |
|  |  |  |  |  |  |  |  |  | Lower | Upper |
| pre-test | Equal variances assumed | .012 | .913 | .379 | 28 | .707 | 1.41817 | 3.73813 | -6.23904 | 9.07537 |
|  | Equal variances not assumed |  |  | .379 | 27.990 | .707 | 1.41817 | 3.73813 | -6.23916 | 9.07550 |
| FZpoPr | Equal variances assumed | 3.972 | .056 | -1.584 | 28 | .124 | -4.55181 | 2.87404 | -10.43900 | 1.33539 |
|  | Equal variances not assumed |  |  | -1.584 | 24.291 | .126 | -4.55181 | 2.87404 | -10.47977 | 1.37616 |
| FYhcPr | Equal variances assumed | 1.376 | .251 | -.768 | 28 | .449 | -2.39388 | 3.11532 | -8.77532 | 3.98757 |
|  | Equal variances not assumed |  |  | -.768 | 24.811 | .449 | -2.39388 | 3.11532 | -8.81247 | 4.02472 |
| FYpoPr | Equal variances assumed | .732 | .400 | -.725 | 28 | .474 | -1.95834 | 2.70091 | -7.49089 | 3.57422 |
|  | Equal variances not assumed |  |  | -.725 | 23.695 | .476 | -1.95834 | 2.70091 | -7.53653 | 3.61986 |
| FXhcPr | Equal variances assumed | 3.726 | .064 | -1.342 | 28 | .190 | -2.56948 | 1.91469 | -6.49154 | 1.35258 |
|  | Equal variances not assumed |  |  | -1.342 | 25.143 | .192 | -2.56948 | 1.91469 | -6.51172 | 1.37275 |
| FXmsPr | Equal variances assumed | .670 | .420 | .163 | 28 | .872 | .15529 | .95474 | -1.80041 | 2.11098 |
|  | Equal variances not assumed |  |  | .163 | 27.862 | .872 | .15529 | .95474 | -1.80084 | 2.11142 |
| FXpoPr | Equal variances assumed | .262 | .613 | .405 | 28 | .688 | .43390 | 1.07110 | -1.76014 | 2.62795 |
|  | Equal variances not assumed |  |  | .405 | 27.709 | .689 | .43390 | 1.07110 | -1.76118 | 2.62899 |

**Baseline comparison for GRF**

| **Group Statistics** | | | | | |
| --- | --- | --- | --- | --- | --- |
|  | group | N | Mean | Std. Deviation | Std. Error Mean |
| pre-test | Experimental | 15 | 1.1538E2 | 10.33528 | 2.66856 |
|  | Control | 15 | 1.1396E2 | 10.13834 | 2.61771 |
| FZpoPr | Experimental | 15 | 1.0855E2 | 9.28218 | 2.39665 |
|  | Control | 15 | 1.1310E2 | 6.14350 | 1.58624 |
| FYhcPr | Experimental | 15 | -30.9418 | 9.94399 | 2.56753 |
|  | Control | 15 | -28.5480 | 6.83340 | 1.76438 |
| FYpoPr | Experimental | 15 | 33.6148 | 8.83350 | 2.28080 |
|  | Control | 15 | 35.5731 | 5.60292 | 1.44667 |
| FXhcPr | Experimental | 15 | 7.6420 | 4.26927 | 1.10232 |
|  | Control | 15 | 10.2115 | 6.06331 | 1.56554 |
| FXmsPr | Experimental | 15 | -11.0149 | 2.70499 | .69843 |
|  | Control | 15 | -11.1702 | 2.52110 | .65094 |
| FXpoPr | Experimental | 15 | -11.2217 | 3.07993 | .79523 |
|  | Control | 15 | -11.6556 | 2.77900 | .71754 |

**Baseline comparison for kinematic data**

| **Group Statistics** | | | | | |
| --- | --- | --- | --- | --- | --- |
|  | group | N | Mean | Std. Deviation | Std. Error Mean |
| pre-test | experience | 15 | -.7851 | 2.47058 | .63790 |
|  | control | 15 | -.6651 | 2.82262 | .72880 |
| A2pr | experience | 15 | 22.0245 | 3.53478 | .91268 |
|  | control | 15 | 22.7707 | 3.52696 | .91066 |
| A3pr | experience | 15 | -3.4074 | 2.86614 | .74003 |
|  | control | 15 | -3.6209 | 3.30597 | .85360 |
| A4pr | experience | 15 | 4.9540 | .85426 | .22057 |
|  | control | 15 | 4.8746 | 1.02730 | .26525 |
| A5pr | experience | 15 | -.3403 | 1.11864 | .28883 |
|  | control | 15 | -.1214 | .82896 | .21404 |
| A6pr | experience | 15 | -9.7750 | 1.12707 | .29101 |
|  | control | 15 | -9.8724 | .73103 | .18875 |
| A7pr | experience | 15 | 5.2724 | .81059 | .20929 |
|  | control | 15 | 5.0399 | .57844 | .14935 |
| K1pr | experience | 15 | 14.2645 | 3.18389 | .82208 |
|  | control | 15 | 13.6502 | 2.70905 | .69947 |
| K2pr | experience | 15 | 1.9249 | 3.12652 | .80726 |
|  | control | 15 | 2.0668 | 3.10702 | .80223 |
| K3pr | experience | 15 | 60.2852 | 4.66108 | 1.20348 |
|  | control | 15 | 61.3560 | 6.69674 | 1.72909 |
| K4pr | experience | 15 | -7.4101 | .65128 | .16816 |
|  | control | 15 | -7.2454 | 1.01948 | .26323 |
| K5pr | experience | 15 | -15.1557 | .84199 | .21740 |
|  | control | 15 | -14.9473 | 1.63658 | .42256 |
| K6pr | experience | 15 | -12.6826 | .89612 | .23138 |
|  | control | 15 | -12.8224 | 1.12196 | .28969 |
| K7pr | experience | 15 | 8.1103 | 2.57001 | .66357 |
|  | control | 15 | 8.1215 | 2.90340 | .74965 |
| H1pr | experience | 15 | -10.0013 | 3.58882 | .92663 |
|  | control | 15 | -9.7568 | 3.52969 | .91136 |
| H2pr | experience | 15 | 3.5936 | 1.03553 | .26737 |
|  | control | 15 | 3.5492 | 1.20396 | .31086 |
| H3pr | experience | 15 | -18.6681 | .99064 | .25578 |
|  | control | 15 | -18.7107 | 1.54908 | .39997 |

| **Independent Samples Test** | | | | | | | | | | |
| --- | --- | --- | --- | --- | --- | --- | --- | --- | --- | --- |
|  |  | Levene's Test for Equality of Variances | | t-test for Equality of Means | | | | | | |
|  |  | F | Sig. | t | df | Sig. (2-tailed) | Mean Difference | Std. Error Difference | 95% Confidence Interval of the Difference | |
|  |  |  |  |  |  |  |  |  | Lower | Upper |
| pre-test | Equal variances assumed | .398 | .533 | -.124 | 28 | .902 | -.12002 | .96854 | -2.10398 | 1.86393 |
|  | Equal variances not assumed |  |  | -.124 | 27.517 | .902 | -.12002 | .96854 | -2.10555 | 1.86550 |
| A2pr | Equal variances assumed | .212 | .648 | -.579 | 28 | .567 | -.74627 | 1.28929 | -3.38727 | 1.89473 |
|  | Equal variances not assumed |  |  | -.579 | 28.000 | .567 | -.74627 | 1.28929 | -3.38727 | 1.89473 |
| A3pr | Equal variances assumed | .519 | .477 | .189 | 28 | .851 | .21349 | 1.12973 | -2.10065 | 2.52763 |
|  | Equal variances not assumed |  |  | .189 | 27.448 | .852 | .21349 | 1.12973 | -2.10275 | 2.52973 |
| A4pr | Equal variances assumed | .611 | .441 | .230 | 28 | .820 | .07939 | .34497 | -.62725 | .78604 |
|  | Equal variances not assumed |  |  | .230 | 27.099 | .820 | .07939 | .34497 | -.62831 | .78710 |
| A5pr | Equal variances assumed | .929 | .343 | -.609 | 28 | .548 | -.21889 | .35949 | -.95528 | .51749 |
|  | Equal variances not assumed |  |  | -.609 | 25.814 | .548 | -.21889 | .35949 | -.95810 | .52031 |
| A6pr | Equal variances assumed | .030 | .863 | .281 | 28 | .781 | .09731 | .34686 | -.61321 | .80782 |
|  | Equal variances not assumed |  |  | .281 | 24.008 | .781 | .09731 | .34686 | -.61857 | .81318 |
| A7pr | Equal variances assumed | .016 | .899 | .905 | 28 | .373 | .23258 | .25712 | -.29411 | .75926 |
|  | Equal variances not assumed |  |  | .905 | 25.322 | .374 | .23258 | .25712 | -.29663 | .76178 |
| K1pr | Equal variances assumed | .506 | .483 | .569 | 28 | .574 | .61432 | 1.07939 | -1.59670 | 2.82534 |
|  | Equal variances not assumed |  |  | .569 | 27.300 | .574 | .61432 | 1.07939 | -1.59926 | 2.82790 |
| K2pr | Equal variances assumed | .044 | .836 | -.125 | 28 | .902 | -.14194 | 1.13809 | -2.47321 | 2.18933 |
|  | Equal variances not assumed |  |  | -.125 | 27.999 | .902 | -.14194 | 1.13809 | -2.47321 | 2.18934 |
| K3pr | Equal variances assumed | .296 | .591 | -.508 | 28 | .615 | -1.07083 | 2.10669 | -5.38618 | 3.24452 |
|  | Equal variances not assumed |  |  | -.508 | 24.986 | .616 | -1.07083 | 2.10669 | -5.40975 | 3.26809 |
| K4pr | Equal variances assumed | 1.970 | .171 | -.527 | 28 | .602 | -.16471 | .31236 | -.80454 | .47513 |
|  | Equal variances not assumed |  |  | -.527 | 23.796 | .603 | -.16471 | .31236 | -.80967 | .48026 |
| K5pr | Equal variances assumed | 4.629 | .040 | -.438 | 28 | .664 | -.20835 | .47521 | -1.18178 | .76507 |
|  | Equal variances not assumed |  |  | -.438 | 20.926 | .666 | -.20835 | .47521 | -1.19682 | .78011 |
| K6pr | Equal variances assumed | 1.021 | .321 | .377 | 28 | .709 | .13973 | .37075 | -.61971 | .89918 |
|  | Equal variances not assumed |  |  | .377 | 26.696 | .709 | .13973 | .37075 | -.62139 | .90086 |
| K7pr | Equal variances assumed | .023 | .880 | -.011 | 28 | .991 | -.01127 | 1.00116 | -2.06204 | 2.03951 |
|  | Equal variances not assumed |  |  | -.011 | 27.594 | .991 | -.01127 | 1.00116 | -2.06340 | 2.04087 |
| H1pr | Equal variances assumed | .404 | .530 | -.188 | 28 | .852 | -.24447 | 1.29970 | -2.90678 | 2.41785 |
|  | Equal variances not assumed |  |  | -.188 | 27.992 | .852 | -.24447 | 1.29970 | -2.90682 | 2.41788 |
| H2pr | Equal variances assumed | .176 | .678 | .108 | 28 | .914 | .04448 | .41003 | -.79542 | .88438 |
|  | Equal variances not assumed |  |  | .108 | 27.387 | .914 | .04448 | .41003 | -.79627 | .88523 |
| H3pr | Equal variances assumed | 2.308 | .140 | .090 | 28 | .929 | .04257 | .47476 | -.92994 | 1.01507 |
|  | Equal variances not assumed |  |  | .090 | 23.810 | .929 | .04257 | .47476 | -.93771 | 1.02284 |

Results of Two Way ANOVA for A1

| **Descriptive Statistics** | | | |
| --- | --- | --- | --- |
|  | Mean | Std. Deviation | N |
| pre-test | -.7851 | 2.47058 | 15 |
| post-test | -2.1422 | 2.10580 | 15 |
| A1prCG | -.6651 | 2.82262 | 15 |
| A1poCG | -1.3007 | 2.72315 | 15 |

| **Multivariate Tests^c^** | | | | | | | | | |
| --- | --- | --- | --- | --- | --- | --- | --- | --- | --- |
| Effect | | Value | F | Hypothesis df | Error df | Sig. | Partial Eta Squared | Noncent. Parameter | Observed Power^b^ |
| groups | Pillai's Trace | .192 | 3.329^a^ | 1.000 | 14.000 | .089 | .192 | 3.329 | .397 |
|  | Wilks' Lambda | .808 | 3.329^a^ | 1.000 | 14.000 | .089 | .192 | 3.329 | .397 |
|  | Hotelling's Trace | .238 | 3.329^a^ | 1.000 | 14.000 | .089 | .192 | 3.329 | .397 |
|  | Roy's Largest Root | .238 | 3.329^a^ | 1.000 | 14.000 | .089 | .192 | 3.329 | .397 |
| time | Pillai's Trace | .165 | 2.774^a^ | 1.000 | 14.000 | .118 | .165 | 2.774 | .342 |
|  | Wilks' Lambda | .835 | 2.774^a^ | 1.000 | 14.000 | .118 | .165 | 2.774 | .342 |
|  | Hotelling's Trace | .198 | 2.774^a^ | 1.000 | 14.000 | .118 | .165 | 2.774 | .342 |
|  | Roy's Largest Root | .198 | 2.774^a^ | 1.000 | 14.000 | .118 | .165 | 2.774 | .342 |
| groups * time | Pillai's Trace | .114 | 1.797^a^ | 1.000 | 14.000 | .201 | .114 | 1.797 | .239 |
|  | Wilks' Lambda | .886 | 1.797^a^ | 1.000 | 14.000 | .201 | .114 | 1.797 | .239 |
|  | Hotelling's Trace | .128 | 1.797^a^ | 1.000 | 14.000 | .201 | .114 | 1.797 | .239 |
|  | Roy's Largest Root | .128 | 1.797^a^ | 1.000 | 14.000 | .201 | .114 | 1.797 | .239 |
| a. Exact statistic | |  |  |  |  |  |  |  |  |
| b. Computed using alpha = .05 | | |  |  |  |  |  |  |  |
| c. Design: Intercept  Within Subjects Design: groups + time + groups * time | | | |  |  |  |  |  |  |

| **Tests of Within-Subjects Effects** | | | | | | | | | |
| --- | --- | --- | --- | --- | --- | --- | --- | --- | --- |
| Measure:MEASURE_1 | |  |  |  |  |  |  |  |  |
| Source | | Type III Sum of Squares | df | Mean Square | F | Sig. | Partial Eta Squared | Noncent. Parameter | Observed Power^a^ |
| groups | Sphericity Assumed | 3.467 | 1 | 3.467 | 3.329 | .089 | .192 | 3.329 | .397 |
|  | Greenhouse-Geisser | 3.467 | 1.000 | 3.467 | 3.329 | .089 | .192 | 3.329 | .397 |
|  | Huynh-Feldt | 3.467 | 1.000 | 3.467 | 3.329 | .089 | .192 | 3.329 | .397 |
|  | Lower-bound | 3.467 | 1.000 | 3.467 | 3.329 | .089 | .192 | 3.329 | .397 |
| Error(groups) | Sphericity Assumed | 14.579 | 14 | 1.041 |  |  |  |  |  |
|  | Greenhouse-Geisser | 14.579 | 14.000 | 1.041 |  |  |  |  |  |
|  | Huynh-Feldt | 14.579 | 14.000 | 1.041 |  |  |  |  |  |
|  | Lower-bound | 14.579 | 14.000 | 1.041 |  |  |  |  |  |
| time | Sphericity Assumed | 14.890 | 1 | 14.890 | 2.774 | .118 | .165 | 2.774 | .342 |
|  | Greenhouse-Geisser | 14.890 | 1.000 | 14.890 | 2.774 | .118 | .165 | 2.774 | .342 |
|  | Huynh-Feldt | 14.890 | 1.000 | 14.890 | 2.774 | .118 | .165 | 2.774 | .342 |
|  | Lower-bound | 14.890 | 1.000 | 14.890 | 2.774 | .118 | .165 | 2.774 | .342 |
| Error(time) | Sphericity Assumed | 75.140 | 14 | 5.367 |  |  |  |  |  |
|  | Greenhouse-Geisser | 75.140 | 14.000 | 5.367 |  |  |  |  |  |
|  | Huynh-Feldt | 75.140 | 14.000 | 5.367 |  |  |  |  |  |
|  | Lower-bound | 75.140 | 14.000 | 5.367 |  |  |  |  |  |
| groups * time | Sphericity Assumed | 1.952 | 1 | 1.952 | 1.797 | .201 | .114 | 1.797 | .239 |
|  | Greenhouse-Geisser | 1.952 | 1.000 | 1.952 | 1.797 | .201 | .114 | 1.797 | .239 |
|  | Huynh-Feldt | 1.952 | 1.000 | 1.952 | 1.797 | .201 | .114 | 1.797 | .239 |
|  | Lower-bound | 1.952 | 1.000 | 1.952 | 1.797 | .201 | .114 | 1.797 | .239 |
| Error(groups*time) | Sphericity Assumed | 15.202 | 14 | 1.086 |  |  |  |  |  |
|  | Greenhouse-Geisser | 15.202 | 14.000 | 1.086 |  |  |  |  |  |
|  | Huynh-Feldt | 15.202 | 14.000 | 1.086 |  |  |  |  |  |
|  | Lower-bound | 15.202 | 14.000 | 1.086 |  |  |  |  |  |
| a. Computed using alpha = .05 | |  |  |  |  |  |  |  |  |

Results of Two Way ANOVA for A2

| **Descriptive Statistics** | | | |
| --- | --- | --- | --- |
|  | Mean | Std. Deviation | N |
| A2pr | 22.0245 | 3.53478 | 15 |
| A2po | 22.3347 | 2.81259 | 15 |
| A2prCG | 22.7707 | 3.52696 | 15 |
| A2poCG | 22.2526 | 3.99059 | 15 |

| **Multivariate Tests^c^** | | | | | | | | | |
| --- | --- | --- | --- | --- | --- | --- | --- | --- | --- |
| Effect | | Value | F | Hypothesis df | Error df | Sig. | Partial Eta Squared | Noncent. Parameter | Observed Power^b^ |
| groups | Pillai's Trace | .020 | .288^a^ | 1.000 | 14.000 | .600 | .020 | .288 | .079 |
|  | Wilks' Lambda | .980 | .288^a^ | 1.000 | 14.000 | .600 | .020 | .288 | .079 |
|  | Hotelling's Trace | .021 | .288^a^ | 1.000 | 14.000 | .600 | .020 | .288 | .079 |
|  | Roy's Largest Root | .021 | .288^a^ | 1.000 | 14.000 | .600 | .020 | .288 | .079 |
| time | Pillai's Trace | .002 | .023^a^ | 1.000 | 14.000 | .882 | .002 | .023 | .052 |
|  | Wilks' Lambda | .998 | .023^a^ | 1.000 | 14.000 | .882 | .002 | .023 | .052 |
|  | Hotelling's Trace | .002 | .023^a^ | 1.000 | 14.000 | .882 | .002 | .023 | .052 |
|  | Roy's Largest Root | .002 | .023^a^ | 1.000 | 14.000 | .882 | .002 | .023 | .052 |
| groups * time | Pillai's Trace | .021 | .302^a^ | 1.000 | 14.000 | .591 | .021 | .302 | .081 |
|  | Wilks' Lambda | .979 | .302^a^ | 1.000 | 14.000 | .591 | .021 | .302 | .081 |
|  | Hotelling's Trace | .022 | .302^a^ | 1.000 | 14.000 | .591 | .021 | .302 | .081 |
|  | Roy's Largest Root | .022 | .302^a^ | 1.000 | 14.000 | .591 | .021 | .302 | .081 |
| a. Exact statistic | |  |  |  |  |  |  |  |  |
| b. Computed using alpha = .05 | | |  |  |  |  |  |  |  |
| c. Design: Intercept  Within Subjects Design: groups + time + groups * time | | | |  |  |  |  |  |  |

| **Tests of Within-Subjects Effects** | | | | | | | | | |
| --- | --- | --- | --- | --- | --- | --- | --- | --- | --- |
| Measure:MEASURE_1 | |  |  |  |  |  |  |  |  |
| Source | | Type III Sum of Squares | df | Mean Square | F | Sig. | Partial Eta Squared | Noncent. Parameter | Observed Power^a^ |
| groups | Sphericity Assumed | 1.654 | 1 | 1.654 | .288 | .600 | .020 | .288 | .079 |
|  | Greenhouse-Geisser | 1.654 | 1.000 | 1.654 | .288 | .600 | .020 | .288 | .079 |
|  | Huynh-Feldt | 1.654 | 1.000 | 1.654 | .288 | .600 | .020 | .288 | .079 |
|  | Lower-bound | 1.654 | 1.000 | 1.654 | .288 | .600 | .020 | .288 | .079 |
| Error(groups) | Sphericity Assumed | 80.317 | 14 | 5.737 |  |  |  |  |  |
|  | Greenhouse-Geisser | 80.317 | 14.000 | 5.737 |  |  |  |  |  |
|  | Huynh-Feldt | 80.317 | 14.000 | 5.737 |  |  |  |  |  |
|  | Lower-bound | 80.317 | 14.000 | 5.737 |  |  |  |  |  |
| time | Sphericity Assumed | .162 | 1 | .162 | .023 | .882 | .002 | .023 | .052 |
|  | Greenhouse-Geisser | .162 | 1.000 | .162 | .023 | .882 | .002 | .023 | .052 |
|  | Huynh-Feldt | .162 | 1.000 | .162 | .023 | .882 | .002 | .023 | .052 |
|  | Lower-bound | .162 | 1.000 | .162 | .023 | .882 | .002 | .023 | .052 |
| Error(time) | Sphericity Assumed | 99.841 | 14 | 7.132 |  |  |  |  |  |
|  | Greenhouse-Geisser | 99.841 | 14.000 | 7.132 |  |  |  |  |  |
|  | Huynh-Feldt | 99.841 | 14.000 | 7.132 |  |  |  |  |  |
|  | Lower-bound | 99.841 | 14.000 | 7.132 |  |  |  |  |  |
| groups * time | Sphericity Assumed | 2.573 | 1 | 2.573 | .302 | .591 | .021 | .302 | .081 |
|  | Greenhouse-Geisser | 2.573 | 1.000 | 2.573 | .302 | .591 | .021 | .302 | .081 |
|  | Huynh-Feldt | 2.573 | 1.000 | 2.573 | .302 | .591 | .021 | .302 | .081 |
|  | Lower-bound | 2.573 | 1.000 | 2.573 | .302 | .591 | .021 | .302 | .081 |
| Error(groups*time) | Sphericity Assumed | 119.194 | 14 | 8.514 |  |  |  |  |  |
|  | Greenhouse-Geisser | 119.194 | 14.000 | 8.514 |  |  |  |  |  |
|  | Huynh-Feldt | 119.194 | 14.000 | 8.514 |  |  |  |  |  |
|  | Lower-bound | 119.194 | 14.000 | 8.514 |  |  |  |  |  |
| a. Computed using alpha = .05 | |  |  |  |  |  |  |  |  |

Results of Two Way ANOVA for A3

| **Descriptive Statistics** | | | |
| --- | --- | --- | --- |
|  | Mean | Std. Deviation | N |
| A3pr | -3.4074 | 2.86614 | 15 |
| A3po | -2.1682 | 4.35033 | 15 |
| A3prCG | -3.6209 | 3.30597 | 15 |
| A3poCG | -4.7038 | 3.18231 | 15 |

| **Multivariate Tests^c^** | | | | | | | | | |
| --- | --- | --- | --- | --- | --- | --- | --- | --- | --- |
| Effect | | Value | F | Hypothesis df | Error df | Sig. | Partial Eta Squared | Noncent. Parameter | Observed Power^b^ |
| groups | Pillai's Trace | .270 | 5.180^a^ | 1.000 | 14.000 | .039 | .270 | 5.180 | .563 |
|  | Wilks' Lambda | .730 | 5.180^a^ | 1.000 | 14.000 | .039 | .270 | 5.180 | .563 |
|  | Hotelling's Trace | .370 | 5.180^a^ | 1.000 | 14.000 | .039 | .270 | 5.180 | .563 |
|  | Roy's Largest Root | .370 | 5.180^a^ | 1.000 | 14.000 | .039 | .270 | 5.180 | .563 |
| time | Pillai's Trace | .001 | .007^a^ | 1.000 | 14.000 | .933 | .001 | .007 | .051 |
|  | Wilks' Lambda | .999 | .007^a^ | 1.000 | 14.000 | .933 | .001 | .007 | .051 |
|  | Hotelling's Trace | .001 | .007^a^ | 1.000 | 14.000 | .933 | .001 | .007 | .051 |
|  | Roy's Largest Root | .001 | .007^a^ | 1.000 | 14.000 | .933 | .001 | .007 | .051 |
| groups * time | Pillai's Trace | .174 | 2.959^a^ | 1.000 | 14.000 | .107 | .174 | 2.959 | .361 |
|  | Wilks' Lambda | .826 | 2.959^a^ | 1.000 | 14.000 | .107 | .174 | 2.959 | .361 |
|  | Hotelling's Trace | .211 | 2.959^a^ | 1.000 | 14.000 | .107 | .174 | 2.959 | .361 |
|  | Roy's Largest Root | .211 | 2.959^a^ | 1.000 | 14.000 | .107 | .174 | 2.959 | .361 |
| a. Exact statistic | |  |  |  |  |  |  |  |  |
| b. Computed using alpha = .05 | | |  |  |  |  |  |  |  |
| c. Design: Intercept  Within Subjects Design: groups + time + groups * time | | | |  |  |  |  |  |  |

| **Tests of Within-Subjects Effects** | | | | | | | | | |
| --- | --- | --- | --- | --- | --- | --- | --- | --- | --- |
| Measure:MEASURE_1 | |  |  |  |  |  |  |  |  |
| Source | | Type III Sum of Squares | df | Mean Square | F | Sig. | Partial Eta Squared | Noncent. Parameter | Observed Power^a^ |
| groups | Sphericity Assumed | 28.341 | 1 | 28.341 | 5.180 | .039 | .270 | 5.180 | .563 |
|  | Greenhouse-Geisser | 28.341 | 1.000 | 28.341 | 5.180 | .039 | .270 | 5.180 | .563 |
|  | Huynh-Feldt | 28.341 | 1.000 | 28.341 | 5.180 | .039 | .270 | 5.180 | .563 |
|  | Lower-bound | 28.341 | 1.000 | 28.341 | 5.180 | .039 | .270 | 5.180 | .563 |
| Error(groups) | Sphericity Assumed | 76.597 | 14 | 5.471 |  |  |  |  |  |
|  | Greenhouse-Geisser | 76.597 | 14.000 | 5.471 |  |  |  |  |  |
|  | Huynh-Feldt | 76.597 | 14.000 | 5.471 |  |  |  |  |  |
|  | Lower-bound | 76.597 | 14.000 | 5.471 |  |  |  |  |  |
| time | Sphericity Assumed | .092 | 1 | .092 | .007 | .933 | .001 | .007 | .051 |
|  | Greenhouse-Geisser | .092 | 1.000 | .092 | .007 | .933 | .001 | .007 | .051 |
|  | Huynh-Feldt | .092 | 1.000 | .092 | .007 | .933 | .001 | .007 | .051 |
|  | Lower-bound | .092 | 1.000 | .092 | .007 | .933 | .001 | .007 | .051 |
| Error(time) | Sphericity Assumed | 172.511 | 14 | 12.322 |  |  |  |  |  |
|  | Greenhouse-Geisser | 172.511 | 14.000 | 12.322 |  |  |  |  |  |
|  | Huynh-Feldt | 172.511 | 14.000 | 12.322 |  |  |  |  |  |
|  | Lower-bound | 172.511 | 14.000 | 12.322 |  |  |  |  |  |
| groups * time | Sphericity Assumed | 20.221 | 1 | 20.221 | 2.959 | .107 | .174 | 2.959 | .361 |
|  | Greenhouse-Geisser | 20.221 | 1.000 | 20.221 | 2.959 | .107 | .174 | 2.959 | .361 |
|  | Huynh-Feldt | 20.221 | 1.000 | 20.221 | 2.959 | .107 | .174 | 2.959 | .361 |
|  | Lower-bound | 20.221 | 1.000 | 20.221 | 2.959 | .107 | .174 | 2.959 | .361 |
| Error(groups*time) | Sphericity Assumed | 95.672 | 14 | 6.834 |  |  |  |  |  |
|  | Greenhouse-Geisser | 95.672 | 14.000 | 6.834 |  |  |  |  |  |
|  | Huynh-Feldt | 95.672 | 14.000 | 6.834 |  |  |  |  |  |
|  | Lower-bound | 95.672 | 14.000 | 6.834 |  |  |  |  |  |
| a. Computed using alpha = .05 | |  |  |  |  |  |  |  |  |

Results of Two Way ANOVA for A2

| **Descriptive Statistics** | | | |
| --- | --- | --- | --- |
|  | Mean | Std. Deviation | N |
| A4pr | 4.9540 | .85426 | 15 |
| A4po | 4.7278 | .79306 | 15 |
| A4prCG | 4.8746 | 1.02730 | 15 |
| A4poCG | 5.0090 | .83470 | 15 |

| **Multivariate Tests^c^** | | | | | | | | | |
| --- | --- | --- | --- | --- | --- | --- | --- | --- | --- |
| Effect | | Value | F | Hypothesis df | Error df | Sig. | Partial Eta Squared | Noncent. Parameter | Observed Power^b^ |
| groups | Pillai's Trace | .010 | .142^a^ | 1.000 | 14.000 | .712 | .010 | .142 | .064 |
|  | Wilks' Lambda | .990 | .142^a^ | 1.000 | 14.000 | .712 | .010 | .142 | .064 |
|  | Hotelling's Trace | .010 | .142^a^ | 1.000 | 14.000 | .712 | .010 | .142 | .064 |
|  | Roy's Largest Root | .010 | .142^a^ | 1.000 | 14.000 | .712 | .010 | .142 | .064 |
| time | Pillai's Trace | .003 | .038^a^ | 1.000 | 14.000 | .848 | .003 | .038 | .054 |
|  | Wilks' Lambda | .997 | .038^a^ | 1.000 | 14.000 | .848 | .003 | .038 | .054 |
|  | Hotelling's Trace | .003 | .038^a^ | 1.000 | 14.000 | .848 | .003 | .038 | .054 |
|  | Roy's Largest Root | .003 | .038^a^ | 1.000 | 14.000 | .848 | .003 | .038 | .054 |
| groups * time | Pillai's Trace | .066 | .991^a^ | 1.000 | 14.000 | .336 | .066 | .991 | .153 |
|  | Wilks' Lambda | .934 | .991^a^ | 1.000 | 14.000 | .336 | .066 | .991 | .153 |
|  | Hotelling's Trace | .071 | .991^a^ | 1.000 | 14.000 | .336 | .066 | .991 | .153 |
|  | Roy's Largest Root | .071 | .991^a^ | 1.000 | 14.000 | .336 | .066 | .991 | .153 |
| a. Exact statistic | |  |  |  |  |  |  |  |  |
| b. Computed using alpha = .05 | | |  |  |  |  |  |  |  |
| c. Design: Intercept  Within Subjects Design: groups + time + groups * time | | | |  |  |  |  |  |  |

| **Tests of Within-Subjects Effects** | | | | | | | | | |
| --- | --- | --- | --- | --- | --- | --- | --- | --- | --- |
| Measure:MEASURE_1 | |  |  |  |  |  |  |  |  |
| Source | | Type III Sum of Squares | df | Mean Square | F | Sig. | Partial Eta Squared | Noncent. Parameter | Observed Power^a^ |
| groups | Sphericity Assumed | .153 | 1 | .153 | .142 | .712 | .010 | .142 | .064 |
|  | Greenhouse-Geisser | .153 | 1.000 | .153 | .142 | .712 | .010 | .142 | .064 |
|  | Huynh-Feldt | .153 | 1.000 | .153 | .142 | .712 | .010 | .142 | .064 |
|  | Lower-bound | .153 | 1.000 | .153 | .142 | .712 | .010 | .142 | .064 |
| Error(groups) | Sphericity Assumed | 15.029 | 14 | 1.073 |  |  |  |  |  |
|  | Greenhouse-Geisser | 15.029 | 14.000 | 1.073 |  |  |  |  |  |
|  | Huynh-Feldt | 15.029 | 14.000 | 1.073 |  |  |  |  |  |
|  | Lower-bound | 15.029 | 14.000 | 1.073 |  |  |  |  |  |
| time | Sphericity Assumed | .032 | 1 | .032 | .038 | .848 | .003 | .038 | .054 |
|  | Greenhouse-Geisser | .032 | 1.000 | .032 | .038 | .848 | .003 | .038 | .054 |
|  | Huynh-Feldt | .032 | 1.000 | .032 | .038 | .848 | .003 | .038 | .054 |
|  | Lower-bound | .032 | 1.000 | .032 | .038 | .848 | .003 | .038 | .054 |
| Error(time) | Sphericity Assumed | 11.527 | 14 | .823 |  |  |  |  |  |
|  | Greenhouse-Geisser | 11.527 | 14.000 | .823 |  |  |  |  |  |
|  | Huynh-Feldt | 11.527 | 14.000 | .823 |  |  |  |  |  |
|  | Lower-bound | 11.527 | 14.000 | .823 |  |  |  |  |  |
| groups * time | Sphericity Assumed | .488 | 1 | .488 | .991 | .336 | .066 | .991 | .153 |
|  | Greenhouse-Geisser | .488 | 1.000 | .488 | .991 | .336 | .066 | .991 | .153 |
|  | Huynh-Feldt | .488 | 1.000 | .488 | .991 | .336 | .066 | .991 | .153 |
|  | Lower-bound | .488 | 1.000 | .488 | .991 | .336 | .066 | .991 | .153 |
| Error(groups*time) | Sphericity Assumed | 6.887 | 14 | .492 |  |  |  |  |  |
|  | Greenhouse-Geisser | 6.887 | 14.000 | .492 |  |  |  |  |  |
|  | Huynh-Feldt | 6.887 | 14.000 | .492 |  |  |  |  |  |
|  | Lower-bound | 6.887 | 14.000 | .492 |  |  |  |  |  |
| a. Computed using alpha = .05 | |  |  |  |  |  |  |  |  |

Results of Two Way ANOVA for A5

| **Descriptive Statistics** | | | |
| --- | --- | --- | --- |
|  | Mean | Std. Deviation | N |
| A5pr | -.3403 | 1.11864 | 15 |
| A5po | .4153 | 1.17282 | 15 |
| A5prCG | -.1214 | .82896 | 15 |
| A5poCG | -.1905 | .81540 | 15 |

| **Multivariate Tests^c^** | | | | | | | | | |
| --- | --- | --- | --- | --- | --- | --- | --- | --- | --- |
| Effect | | Value | F | Hypothesis df | Error df | Sig. | Partial Eta Squared | Noncent. Parameter | Observed Power^b^ |
| groups | Pillai's Trace | .034 | .492^a^ | 1.000 | 14.000 | .494 | .034 | .492 | .100 |
|  | Wilks' Lambda | .966 | .492^a^ | 1.000 | 14.000 | .494 | .034 | .492 | .100 |
|  | Hotelling's Trace | .035 | .492^a^ | 1.000 | 14.000 | .494 | .034 | .492 | .100 |
|  | Roy's Largest Root | .035 | .492^a^ | 1.000 | 14.000 | .494 | .034 | .492 | .100 |
| time | Pillai's Trace | .136 | 2.198^a^ | 1.000 | 14.000 | .160 | .136 | 2.198 | .282 |
|  | Wilks' Lambda | .864 | 2.198^a^ | 1.000 | 14.000 | .160 | .136 | 2.198 | .282 |
|  | Hotelling's Trace | .157 | 2.198^a^ | 1.000 | 14.000 | .160 | .136 | 2.198 | .282 |
|  | Roy's Largest Root | .157 | 2.198^a^ | 1.000 | 14.000 | .160 | .136 | 2.198 | .282 |
| groups * time | Pillai's Trace | .251 | 4.687^a^ | 1.000 | 14.000 | .048 | .251 | 4.687 | .522 |
|  | Wilks' Lambda | .749 | 4.687^a^ | 1.000 | 14.000 | .048 | .251 | 4.687 | .522 |
|  | Hotelling's Trace | .335 | 4.687^a^ | 1.000 | 14.000 | .048 | .251 | 4.687 | .522 |
|  | Roy's Largest Root | .335 | 4.687^a^ | 1.000 | 14.000 | .048 | .251 | 4.687 | .522 |
| a. Exact statistic | |  |  |  |  |  |  |  |  |
| b. Computed using alpha = .05 | | |  |  |  |  |  |  |  |
| c. Design: Intercept  Within Subjects Design: groups + time + groups * time | | | |  |  |  |  |  |  |

| **Tests of Within-Subjects Effects** | | | | | | | | | |
| --- | --- | --- | --- | --- | --- | --- | --- | --- | --- |
| Measure:MEASURE_1 | |  |  |  |  |  |  |  |  |
| Source | | Type III Sum of Squares | df | Mean Square | F | Sig. | Partial Eta Squared | Noncent. Parameter | Observed Power^a^ |
| groups | Sphericity Assumed | .562 | 1 | .562 | .492 | .494 | .034 | .492 | .100 |
|  | Greenhouse-Geisser | .562 | 1.000 | .562 | .492 | .494 | .034 | .492 | .100 |
|  | Huynh-Feldt | .562 | 1.000 | .562 | .492 | .494 | .034 | .492 | .100 |
|  | Lower-bound | .562 | 1.000 | .562 | .492 | .494 | .034 | .492 | .100 |
| Error(groups) | Sphericity Assumed | 15.975 | 14 | 1.141 |  |  |  |  |  |
|  | Greenhouse-Geisser | 15.975 | 14.000 | 1.141 |  |  |  |  |  |
|  | Huynh-Feldt | 15.975 | 14.000 | 1.141 |  |  |  |  |  |
|  | Lower-bound | 15.975 | 14.000 | 1.141 |  |  |  |  |  |
| time | Sphericity Assumed | 1.767 | 1 | 1.767 | 2.198 | .160 | .136 | 2.198 | .282 |
|  | Greenhouse-Geisser | 1.767 | 1.000 | 1.767 | 2.198 | .160 | .136 | 2.198 | .282 |
|  | Huynh-Feldt | 1.767 | 1.000 | 1.767 | 2.198 | .160 | .136 | 2.198 | .282 |
|  | Lower-bound | 1.767 | 1.000 | 1.767 | 2.198 | .160 | .136 | 2.198 | .282 |
| Error(time) | Sphericity Assumed | 11.253 | 14 | .804 |  |  |  |  |  |
|  | Greenhouse-Geisser | 11.253 | 14.000 | .804 |  |  |  |  |  |
|  | Huynh-Feldt | 11.253 | 14.000 | .804 |  |  |  |  |  |
|  | Lower-bound | 11.253 | 14.000 | .804 |  |  |  |  |  |
| groups * time | Sphericity Assumed | 2.551 | 1 | 2.551 | 4.687 | .048 | .251 | 4.687 | .522 |
|  | Greenhouse-Geisser | 2.551 | 1.000 | 2.551 | 4.687 | .048 | .251 | 4.687 | .522 |
|  | Huynh-Feldt | 2.551 | 1.000 | 2.551 | 4.687 | .048 | .251 | 4.687 | .522 |
|  | Lower-bound | 2.551 | 1.000 | 2.551 | 4.687 | .048 | .251 | 4.687 | .522 |
| Error(groups*time) | Sphericity Assumed | 7.618 | 14 | .544 |  |  |  |  |  |
|  | Greenhouse-Geisser | 7.618 | 14.000 | .544 |  |  |  |  |  |
|  | Huynh-Feldt | 7.618 | 14.000 | .544 |  |  |  |  |  |
|  | Lower-bound | 7.618 | 14.000 | .544 |  |  |  |  |  |
| a. Computed using alpha = .05 | |  |  |  |  |  |  |  |  |

Results of Two Way ANOVA for A6

| **Descriptive Statistics** | | | |
| --- | --- | --- | --- |
|  | Mean | Std. Deviation | N |
| A6pr | -9.7750 | 1.12707 | 15 |
| A6po | -10.0394 | .30583 | 15 |
| A6prCG | -9.8724 | .73103 | 15 |
| A6poCG | -9.6058 | 1.74923 | 15 |

| **Multivariate Tests^c^** | | | | | | | | | |
| --- | --- | --- | --- | --- | --- | --- | --- | --- | --- |
| Effect | | Value | F | Hypothesis df | Error df | Sig. | Partial Eta Squared | Noncent. Parameter | Observed Power^b^ |
| groups | Pillai's Trace | .019 | .270^a^ | 1.000 | 14.000 | .612 | .019 | .270 | .077 |
|  | Wilks' Lambda | .981 | .270^a^ | 1.000 | 14.000 | .612 | .019 | .270 | .077 |
|  | Hotelling's Trace | .019 | .270^a^ | 1.000 | 14.000 | .612 | .019 | .270 | .077 |
|  | Roy's Largest Root | .019 | .270^a^ | 1.000 | 14.000 | .612 | .019 | .270 | .077 |
| time | Pillai's Trace | .000 | .000^a^ | 1.000 | 14.000 | .997 | .000 | .000 | .050 |
|  | Wilks' Lambda | 1.000 | .000^a^ | 1.000 | 14.000 | .997 | .000 | .000 | .050 |
|  | Hotelling's Trace | .000 | .000^a^ | 1.000 | 14.000 | .997 | .000 | .000 | .050 |
|  | Roy's Largest Root | .000 | .000^a^ | 1.000 | 14.000 | .997 | .000 | .000 | .050 |
| groups * time | Pillai's Trace | .083 | 1.273^a^ | 1.000 | 14.000 | .278 | .083 | 1.273 | .183 |
|  | Wilks' Lambda | .917 | 1.273^a^ | 1.000 | 14.000 | .278 | .083 | 1.273 | .183 |
|  | Hotelling's Trace | .091 | 1.273^a^ | 1.000 | 14.000 | .278 | .083 | 1.273 | .183 |
|  | Roy's Largest Root | .091 | 1.273^a^ | 1.000 | 14.000 | .278 | .083 | 1.273 | .183 |
| a. Exact statistic | |  |  |  |  |  |  |  |  |
| b. Computed using alpha = .05 | | |  |  |  |  |  |  |  |
| c. Design: Intercept  Within Subjects Design: groups + time + groups * time | | | |  |  |  |  |  |  |

| **Tests of Within-Subjects Effects** | | | | | | | | | |
| --- | --- | --- | --- | --- | --- | --- | --- | --- | --- |
| Measure:MEASURE_1 | |  |  |  |  |  |  |  |  |
| Source | | Type III Sum of Squares | df | Mean Square | F | Sig. | Partial Eta Squared | Noncent. Parameter | Observed Power^a^ |
| groups | Sphericity Assumed | .424 | 1 | .424 | .270 | .612 | .019 | .270 | .077 |
|  | Greenhouse-Geisser | .424 | 1.000 | .424 | .270 | .612 | .019 | .270 | .077 |
|  | Huynh-Feldt | .424 | 1.000 | .424 | .270 | .612 | .019 | .270 | .077 |
|  | Lower-bound | .424 | 1.000 | .424 | .270 | .612 | .019 | .270 | .077 |
| Error(groups) | Sphericity Assumed | 22.023 | 14 | 1.573 |  |  |  |  |  |
|  | Greenhouse-Geisser | 22.023 | 14.000 | 1.573 |  |  |  |  |  |
|  | Huynh-Feldt | 22.023 | 14.000 | 1.573 |  |  |  |  |  |
|  | Lower-bound | 22.023 | 14.000 | 1.573 |  |  |  |  |  |
| time | Sphericity Assumed | 1.777E-5 | 1 | 1.777E-5 | .000 | .997 | .000 | .000 | .050 |
|  | Greenhouse-Geisser | 1.777E-5 | 1.000 | 1.777E-5 | .000 | .997 | .000 | .000 | .050 |
|  | Huynh-Feldt | 1.777E-5 | 1.000 | 1.777E-5 | .000 | .997 | .000 | .000 | .050 |
|  | Lower-bound | 1.777E-5 | 1.000 | 1.777E-5 | .000 | .997 | .000 | .000 | .050 |
| Error(time) | Sphericity Assumed | 23.928 | 14 | 1.709 |  |  |  |  |  |
|  | Greenhouse-Geisser | 23.928 | 14.000 | 1.709 |  |  |  |  |  |
|  | Huynh-Feldt | 23.928 | 14.000 | 1.709 |  |  |  |  |  |
|  | Lower-bound | 23.928 | 14.000 | 1.709 |  |  |  |  |  |
| groups * time | Sphericity Assumed | 1.057 | 1 | 1.057 | 1.273 | .278 | .083 | 1.273 | .183 |
|  | Greenhouse-Geisser | 1.057 | 1.000 | 1.057 | 1.273 | .278 | .083 | 1.273 | .183 |
|  | Huynh-Feldt | 1.057 | 1.000 | 1.057 | 1.273 | .278 | .083 | 1.273 | .183 |
|  | Lower-bound | 1.057 | 1.000 | 1.057 | 1.273 | .278 | .083 | 1.273 | .183 |
| Error(groups*time) | Sphericity Assumed | 11.623 | 14 | .830 |  |  |  |  |  |
|  | Greenhouse-Geisser | 11.623 | 14.000 | .830 |  |  |  |  |  |
|  | Huynh-Feldt | 11.623 | 14.000 | .830 |  |  |  |  |  |
|  | Lower-bound | 11.623 | 14.000 | .830 |  |  |  |  |  |
| a. Computed using alpha = .05 | |  |  |  |  |  |  |  |  |

Results of Two Way ANOVA for A7

| **Descriptive Statistics** | | | |
| --- | --- | --- | --- |
|  | Mean | Std. Deviation | N |
| A7pr | 5.2724 | .81059 | 15 |
| A7po | 3.3364 | 1.22939 | 15 |
| A7prCG | 5.0399 | .57844 | 15 |
| A7poCG | 5.3987 | 1.22672 | 15 |

| **Multivariate Tests^c^** | | | | | | | | | |
| --- | --- | --- | --- | --- | --- | --- | --- | --- | --- |
| Effect | | Value | F | Hypothesis df | Error df | Sig. | Partial Eta Squared | Noncent. Parameter | Observed Power^b^ |
| groups | Pillai's Trace | .504 | 14.234^a^ | 1.000 | 14.000 | .002 | .504 | 14.234 | .939 |
|  | Wilks' Lambda | .496 | 14.234^a^ | 1.000 | 14.000 | .002 | .504 | 14.234 | .939 |
|  | Hotelling's Trace | 1.017 | 14.234^a^ | 1.000 | 14.000 | .002 | .504 | 14.234 | .939 |
|  | Roy's Largest Root | 1.017 | 14.234^a^ | 1.000 | 14.000 | .002 | .504 | 14.234 | .939 |
| time | Pillai's Trace | .423 | 10.259^a^ | 1.000 | 14.000 | .006 | .423 | 10.259 | .845 |
|  | Wilks' Lambda | .577 | 10.259^a^ | 1.000 | 14.000 | .006 | .423 | 10.259 | .845 |
|  | Hotelling's Trace | .733 | 10.259^a^ | 1.000 | 14.000 | .006 | .423 | 10.259 | .845 |
|  | Roy's Largest Root | .733 | 10.259^a^ | 1.000 | 14.000 | .006 | .423 | 10.259 | .845 |
| groups * time | Pillai's Trace | .597 | 20.756^a^ | 1.000 | 14.000 | .000 | .597 | 20.756 | .988 |
|  | Wilks' Lambda | .403 | 20.756^a^ | 1.000 | 14.000 | .000 | .597 | 20.756 | .988 |
|  | Hotelling's Trace | 1.483 | 20.756^a^ | 1.000 | 14.000 | .000 | .597 | 20.756 | .988 |
|  | Roy's Largest Root | 1.483 | 20.756^a^ | 1.000 | 14.000 | .000 | .597 | 20.756 | .988 |
| a. Exact statistic | |  |  |  |  |  |  |  |  |
| b. Computed using alpha = .05 | | |  |  |  |  |  |  |  |
| c. Design: Intercept  Within Subjects Design: groups + time + groups * time | | | |  |  |  |  |  |  |

| **Tests of Within-Subjects Effects** | | | | | | | | | |
| --- | --- | --- | --- | --- | --- | --- | --- | --- | --- |
| Measure:MEASURE_1 | |  |  |  |  |  |  |  |  |
| Source | | Type III Sum of Squares | df | Mean Square | F | Sig. | Partial Eta Squared | Noncent. Parameter | Observed Power^a^ |
| groups | Sphericity Assumed | 12.555 | 1 | 12.555 | 14.234 | .002 | .504 | 14.234 | .939 |
|  | Greenhouse-Geisser | 12.555 | 1.000 | 12.555 | 14.234 | .002 | .504 | 14.234 | .939 |
|  | Huynh-Feldt | 12.555 | 1.000 | 12.555 | 14.234 | .002 | .504 | 14.234 | .939 |
|  | Lower-bound | 12.555 | 1.000 | 12.555 | 14.234 | .002 | .504 | 14.234 | .939 |
| Error(groups) | Sphericity Assumed | 12.349 | 14 | .882 |  |  |  |  |  |
|  | Greenhouse-Geisser | 12.349 | 14.000 | .882 |  |  |  |  |  |
|  | Huynh-Feldt | 12.349 | 14.000 | .882 |  |  |  |  |  |
|  | Lower-bound | 12.349 | 14.000 | .882 |  |  |  |  |  |
| time | Sphericity Assumed | 9.328 | 1 | 9.328 | 10.259 | .006 | .423 | 10.259 | .845 |
|  | Greenhouse-Geisser | 9.328 | 1.000 | 9.328 | 10.259 | .006 | .423 | 10.259 | .845 |
|  | Huynh-Feldt | 9.328 | 1.000 | 9.328 | 10.259 | .006 | .423 | 10.259 | .845 |
|  | Lower-bound | 9.328 | 1.000 | 9.328 | 10.259 | .006 | .423 | 10.259 | .845 |
| Error(time) | Sphericity Assumed | 12.730 | 14 | .909 |  |  |  |  |  |
|  | Greenhouse-Geisser | 12.730 | 14.000 | .909 |  |  |  |  |  |
|  | Huynh-Feldt | 12.730 | 14.000 | .909 |  |  |  |  |  |
|  | Lower-bound | 12.730 | 14.000 | .909 |  |  |  |  |  |
| groups * time | Sphericity Assumed | 19.749 | 1 | 19.749 | 20.756 | .000 | .597 | 20.756 | .988 |
|  | Greenhouse-Geisser | 19.749 | 1.000 | 19.749 | 20.756 | .000 | .597 | 20.756 | .988 |
|  | Huynh-Feldt | 19.749 | 1.000 | 19.749 | 20.756 | .000 | .597 | 20.756 | .988 |
|  | Lower-bound | 19.749 | 1.000 | 19.749 | 20.756 | .000 | .597 | 20.756 | .988 |
| Error(groups*time) | Sphericity Assumed | 13.321 | 14 | .951 |  |  |  |  |  |
|  | Greenhouse-Geisser | 13.321 | 14.000 | .951 |  |  |  |  |  |
|  | Huynh-Feldt | 13.321 | 14.000 | .951 |  |  |  |  |  |
|  | Lower-bound | 13.321 | 14.000 | .951 |  |  |  |  |  |
| a. Computed using alpha = .05 | |  |  |  |  |  |  |  |  |

Results of Two Way ANOVA for k1

| **Descriptive Statistics** | | | |
| --- | --- | --- | --- |
|  | Mean | Std. Deviation | N |
| K1pr | 14.2645 | 3.18389 | 15 |
| K1po | 16.2826 | 1.97116 | 15 |
| K1prCG | 13.6502 | 2.70905 | 15 |
| K2poCG | 1.3953 | 2.56566 | 15 |

| **Multivariate Tests^c^** | | | | | | | | | |
| --- | --- | --- | --- | --- | --- | --- | --- | --- | --- |
| Effect | | Value | F | Hypothesis df | Error df | Sig. | Partial Eta Squared | Noncent. Parameter | Observed Power^b^ |
| groups | Pillai's Trace | .943 | 2.299E2^a^ | 1.000 | 14.000 | .000 | .943 | 229.862 | 1.000 |
|  | Wilks' Lambda | .057 | 2.299E2^a^ | 1.000 | 14.000 | .000 | .943 | 229.862 | 1.000 |
|  | Hotelling's Trace | 16.419 | 2.299E2^a^ | 1.000 | 14.000 | .000 | .943 | 229.862 | 1.000 |
|  | Roy's Largest Root | 16.419 | 2.299E2^a^ | 1.000 | 14.000 | .000 | .943 | 229.862 | 1.000 |
| time | Pillai's Trace | .793 | 53.711^a^ | 1.000 | 14.000 | .000 | .793 | 53.711 | 1.000 |
|  | Wilks' Lambda | .207 | 53.711^a^ | 1.000 | 14.000 | .000 | .793 | 53.711 | 1.000 |
|  | Hotelling's Trace | 3.837 | 53.711^a^ | 1.000 | 14.000 | .000 | .793 | 53.711 | 1.000 |
|  | Roy's Largest Root | 3.837 | 53.711^a^ | 1.000 | 14.000 | .000 | .793 | 53.711 | 1.000 |
| groups * time | Pillai's Trace | .944 | 2.342E2^a^ | 1.000 | 14.000 | .000 | .944 | 234.240 | 1.000 |
|  | Wilks' Lambda | .056 | 2.342E2^a^ | 1.000 | 14.000 | .000 | .944 | 234.240 | 1.000 |
|  | Hotelling's Trace | 16.731 | 2.342E2^a^ | 1.000 | 14.000 | .000 | .944 | 234.240 | 1.000 |
|  | Roy's Largest Root | 16.731 | 2.342E2^a^ | 1.000 | 14.000 | .000 | .944 | 234.240 | 1.000 |
| a. Exact statistic | |  |  |  |  |  |  |  |  |
| b. Computed using alpha = .05 | | |  |  |  |  |  |  |  |
| c. Design: Intercept  Within Subjects Design: groups + time + groups * time | | | |  |  |  |  |  |  |

| **Tests of Within-Subjects Effects** | | | | | | | | | |
| --- | --- | --- | --- | --- | --- | --- | --- | --- | --- |
| Measure:MEASURE_1 | |  |  |  |  |  |  |  |  |
| Source | | Type III Sum of Squares | df | Mean Square | F | Sig. | Partial Eta Squared | Noncent. Parameter | Observed Power^a^ |
| groups | Sphericity Assumed | 901.121 | 1 | 901.121 | 229.862 | .000 | .943 | 229.862 | 1.000 |
|  | Greenhouse-Geisser | 901.121 | 1.000 | 901.121 | 229.862 | .000 | .943 | 229.862 | 1.000 |
|  | Huynh-Feldt | 901.121 | 1.000 | 901.121 | 229.862 | .000 | .943 | 229.862 | 1.000 |
|  | Lower-bound | 901.121 | 1.000 | 901.121 | 229.862 | .000 | .943 | 229.862 | 1.000 |
| Error(groups) | Sphericity Assumed | 54.884 | 14 | 3.920 |  |  |  |  |  |
|  | Greenhouse-Geisser | 54.884 | 14.000 | 3.920 |  |  |  |  |  |
|  | Huynh-Feldt | 54.884 | 14.000 | 3.920 |  |  |  |  |  |
|  | Lower-bound | 54.884 | 14.000 | 3.920 |  |  |  |  |  |
| time | Sphericity Assumed | 392.966 | 1 | 392.966 | 53.711 | .000 | .793 | 53.711 | 1.000 |
|  | Greenhouse-Geisser | 392.966 | 1.000 | 392.966 | 53.711 | .000 | .793 | 53.711 | 1.000 |
|  | Huynh-Feldt | 392.966 | 1.000 | 392.966 | 53.711 | .000 | .793 | 53.711 | 1.000 |
|  | Lower-bound | 392.966 | 1.000 | 392.966 | 53.711 | .000 | .793 | 53.711 | 1.000 |
| Error(time) | Sphericity Assumed | 102.428 | 14 | 7.316 |  |  |  |  |  |
|  | Greenhouse-Geisser | 102.428 | 14.000 | 7.316 |  |  |  |  |  |
|  | Huynh-Feldt | 102.428 | 14.000 | 7.316 |  |  |  |  |  |
|  | Lower-bound | 102.428 | 14.000 | 7.316 |  |  |  |  |  |
| groups * time | Sphericity Assumed | 763.938 | 1 | 763.938 | 234.240 | .000 | .944 | 234.240 | 1.000 |
|  | Greenhouse-Geisser | 763.938 | 1.000 | 763.938 | 234.240 | .000 | .944 | 234.240 | 1.000 |
|  | Huynh-Feldt | 763.938 | 1.000 | 763.938 | 234.240 | .000 | .944 | 234.240 | 1.000 |
|  | Lower-bound | 763.938 | 1.000 | 763.938 | 234.240 | .000 | .944 | 234.240 | 1.000 |
| Error(groups*time) | Sphericity Assumed | 45.659 | 14 | 3.261 |  |  |  |  |  |
|  | Greenhouse-Geisser | 45.659 | 14.000 | 3.261 |  |  |  |  |  |
|  | Huynh-Feldt | 45.659 | 14.000 | 3.261 |  |  |  |  |  |
|  | Lower-bound | 45.659 | 14.000 | 3.261 |  |  |  |  |  |
| a. Computed using alpha = .05 | |  |  |  |  |  |  |  |  |

Results of Two Way ANOVA for k2

| **Descriptive Statistics** | | | |
| --- | --- | --- | --- |
|  | Mean | Std. Deviation | N |
| K2pr | 1.9249 | 3.12652 | 15 |
| K2po | 1.5080 | .91878 | 15 |
| K2prCG | 2.0668 | 3.10702 | 15 |
| K2poCG | 1.3953 | 2.56566 | 15 |

| **Multivariate Tests^c^** | | | | | | | | | |
| --- | --- | --- | --- | --- | --- | --- | --- | --- | --- |
| Effect | | Value | F | Hypothesis df | Error df | Sig. | Partial Eta Squared | Noncent. Parameter | Observed Power^b^ |
| groups | Pillai's Trace | .000 | .001^a^ | 1.000 | 14.000 | .972 | .000 | .001 | .050 |
|  | Wilks' Lambda | 1.000 | .001^a^ | 1.000 | 14.000 | .972 | .000 | .001 | .050 |
|  | Hotelling's Trace | .000 | .001^a^ | 1.000 | 14.000 | .972 | .000 | .001 | .050 |
|  | Roy's Largest Root | .000 | .001^a^ | 1.000 | 14.000 | .972 | .000 | .001 | .050 |
| time | Pillai's Trace | .038 | .550^a^ | 1.000 | 14.000 | .471 | .038 | .550 | .106 |
|  | Wilks' Lambda | .962 | .550^a^ | 1.000 | 14.000 | .471 | .038 | .550 | .106 |
|  | Hotelling's Trace | .039 | .550^a^ | 1.000 | 14.000 | .471 | .038 | .550 | .106 |
|  | Roy's Largest Root | .039 | .550^a^ | 1.000 | 14.000 | .471 | .038 | .550 | .106 |
| groups * time | Pillai's Trace | .008 | .115^a^ | 1.000 | 14.000 | .740 | .008 | .115 | .062 |
|  | Wilks' Lambda | .992 | .115^a^ | 1.000 | 14.000 | .740 | .008 | .115 | .062 |
|  | Hotelling's Trace | .008 | .115^a^ | 1.000 | 14.000 | .740 | .008 | .115 | .062 |
|  | Roy's Largest Root | .008 | .115^a^ | 1.000 | 14.000 | .740 | .008 | .115 | .062 |
| a. Exact statistic | |  |  |  |  |  |  |  |  |
| b. Computed using alpha = .05 | | |  |  |  |  |  |  |  |
| c. Design: Intercept  Within Subjects Design: groups + time + groups * time | | | |  |  |  |  |  |  |

| **Tests of Within-Subjects Effects** | | | | | | | | | |
| --- | --- | --- | --- | --- | --- | --- | --- | --- | --- |
| Measure:MEASURE_1 | |  |  |  |  |  |  |  |  |
| Source | | Type III Sum of Squares | df | Mean Square | F | Sig. | Partial Eta Squared | Noncent. Parameter | Observed Power^a^ |
| groups | Sphericity Assumed | .003 | 1 | .003 | .001 | .972 | .000 | .001 | .050 |
|  | Greenhouse-Geisser | .003 | 1.000 | .003 | .001 | .972 | .000 | .001 | .050 |
|  | Huynh-Feldt | .003 | 1.000 | .003 | .001 | .972 | .000 | .001 | .050 |
|  | Lower-bound | .003 | 1.000 | .003 | .001 | .972 | .000 | .001 | .050 |
| Error(groups) | Sphericity Assumed | 34.217 | 14 | 2.444 |  |  |  |  |  |
|  | Greenhouse-Geisser | 34.217 | 14.000 | 2.444 |  |  |  |  |  |
|  | Huynh-Feldt | 34.217 | 14.000 | 2.444 |  |  |  |  |  |
|  | Lower-bound | 34.217 | 14.000 | 2.444 |  |  |  |  |  |
| time | Sphericity Assumed | 4.441 | 1 | 4.441 | .550 | .471 | .038 | .550 | .106 |
|  | Greenhouse-Geisser | 4.441 | 1.000 | 4.441 | .550 | .471 | .038 | .550 | .106 |
|  | Huynh-Feldt | 4.441 | 1.000 | 4.441 | .550 | .471 | .038 | .550 | .106 |
|  | Lower-bound | 4.441 | 1.000 | 4.441 | .550 | .471 | .038 | .550 | .106 |
| Error(time) | Sphericity Assumed | 113.122 | 14 | 8.080 |  |  |  |  |  |
|  | Greenhouse-Geisser | 113.122 | 14.000 | 8.080 |  |  |  |  |  |
|  | Huynh-Feldt | 113.122 | 14.000 | 8.080 |  |  |  |  |  |
|  | Lower-bound | 113.122 | 14.000 | 8.080 |  |  |  |  |  |
| groups * time | Sphericity Assumed | .243 | 1 | .243 | .115 | .740 | .008 | .115 | .062 |
|  | Greenhouse-Geisser | .243 | 1.000 | .243 | .115 | .740 | .008 | .115 | .062 |
|  | Huynh-Feldt | .243 | 1.000 | .243 | .115 | .740 | .008 | .115 | .062 |
|  | Lower-bound | .243 | 1.000 | .243 | .115 | .740 | .008 | .115 | .062 |
| Error(groups*time) | Sphericity Assumed | 29.735 | 14 | 2.124 |  |  |  |  |  |
|  | Greenhouse-Geisser | 29.735 | 14.000 | 2.124 |  |  |  |  |  |
|  | Huynh-Feldt | 29.735 | 14.000 | 2.124 |  |  |  |  |  |
|  | Lower-bound | 29.735 | 14.000 | 2.124 |  |  |  |  |  |
| a. Computed using alpha = .05 | |  |  |  |  |  |  |  |  |

Results of Two Way ANOVA for k3

| **Descriptive Statistics** | | | |
| --- | --- | --- | --- |
|  | Mean | Std. Deviation | N |
| K3pr | 60.2852 | 4.66108 | 15 |
| K3po | 60.3551 | 2.35632 | 15 |
| K3prCG | 61.3560 | 6.69674 | 15 |
| K3poCG | 60.6446 | 7.83381 | 15 |

| **Multivariate Tests^c^** | | | | | | | | | |
| --- | --- | --- | --- | --- | --- | --- | --- | --- | --- |
| Effect | | Value | F | Hypothesis df | Error df | Sig. | Partial Eta Squared | Noncent. Parameter | Observed Power^b^ |
| groups | Pillai's Trace | .044 | .639^a^ | 1.000 | 14.000 | .437 | .044 | .639 | .116 |
|  | Wilks' Lambda | .956 | .639^a^ | 1.000 | 14.000 | .437 | .044 | .639 | .116 |
|  | Hotelling's Trace | .046 | .639^a^ | 1.000 | 14.000 | .437 | .044 | .639 | .116 |
|  | Roy's Largest Root | .046 | .639^a^ | 1.000 | 14.000 | .437 | .044 | .639 | .116 |
| time | Pillai's Trace | .002 | .029^a^ | 1.000 | 14.000 | .867 | .002 | .029 | .053 |
|  | Wilks' Lambda | .998 | .029^a^ | 1.000 | 14.000 | .867 | .002 | .029 | .053 |
|  | Hotelling's Trace | .002 | .029^a^ | 1.000 | 14.000 | .867 | .002 | .029 | .053 |
|  | Roy's Largest Root | .002 | .029^a^ | 1.000 | 14.000 | .867 | .002 | .029 | .053 |
| groups * time | Pillai's Trace | .006 | .081^a^ | 1.000 | 14.000 | .780 | .006 | .081 | .058 |
|  | Wilks' Lambda | .994 | .081^a^ | 1.000 | 14.000 | .780 | .006 | .081 | .058 |
|  | Hotelling's Trace | .006 | .081^a^ | 1.000 | 14.000 | .780 | .006 | .081 | .058 |
|  | Roy's Largest Root | .006 | .081^a^ | 1.000 | 14.000 | .780 | .006 | .081 | .058 |
| a. Exact statistic | |  |  |  |  |  |  |  |  |
| b. Computed using alpha = .05 | | |  |  |  |  |  |  |  |
| c. Design: Intercept  Within Subjects Design: groups + time + groups * time | | | |  |  |  |  |  |  |

| **Tests of Within-Subjects Effects** | | | | | | | | | |
| --- | --- | --- | --- | --- | --- | --- | --- | --- | --- |
| Measure:MEASURE_1 | |  |  |  |  |  |  |  |  |
| Source | | Type III Sum of Squares | df | Mean Square | F | Sig. | Partial Eta Squared | Noncent. Parameter | Observed Power^a^ |
| groups | Sphericity Assumed | 6.940 | 1 | 6.940 | .639 | .437 | .044 | .639 | .116 |
|  | Greenhouse-Geisser | 6.940 | 1.000 | 6.940 | .639 | .437 | .044 | .639 | .116 |
|  | Huynh-Feldt | 6.940 | 1.000 | 6.940 | .639 | .437 | .044 | .639 | .116 |
|  | Lower-bound | 6.940 | 1.000 | 6.940 | .639 | .437 | .044 | .639 | .116 |
| Error(groups) | Sphericity Assumed | 152.057 | 14 | 10.861 |  |  |  |  |  |
|  | Greenhouse-Geisser | 152.057 | 14.000 | 10.861 |  |  |  |  |  |
|  | Huynh-Feldt | 152.057 | 14.000 | 10.861 |  |  |  |  |  |
|  | Lower-bound | 152.057 | 14.000 | 10.861 |  |  |  |  |  |
| time | Sphericity Assumed | 1.543 | 1 | 1.543 | .029 | .867 | .002 | .029 | .053 |
|  | Greenhouse-Geisser | 1.543 | 1.000 | 1.543 | .029 | .867 | .002 | .029 | .053 |
|  | Huynh-Feldt | 1.543 | 1.000 | 1.543 | .029 | .867 | .002 | .029 | .053 |
|  | Lower-bound | 1.543 | 1.000 | 1.543 | .029 | .867 | .002 | .029 | .053 |
| Error(time) | Sphericity Assumed | 740.779 | 14 | 52.913 |  |  |  |  |  |
|  | Greenhouse-Geisser | 740.779 | 14.000 | 52.913 |  |  |  |  |  |
|  | Huynh-Feldt | 740.779 | 14.000 | 52.913 |  |  |  |  |  |
|  | Lower-bound | 740.779 | 14.000 | 52.913 |  |  |  |  |  |
| groups * time | Sphericity Assumed | 2.289 | 1 | 2.289 | .081 | .780 | .006 | .081 | .058 |
|  | Greenhouse-Geisser | 2.289 | 1.000 | 2.289 | .081 | .780 | .006 | .081 | .058 |
|  | Huynh-Feldt | 2.289 | 1.000 | 2.289 | .081 | .780 | .006 | .081 | .058 |
|  | Lower-bound | 2.289 | 1.000 | 2.289 | .081 | .780 | .006 | .081 | .058 |
| Error(groups*time) | Sphericity Assumed | 393.568 | 14 | 28.112 |  |  |  |  |  |
|  | Greenhouse-Geisser | 393.568 | 14.000 | 28.112 |  |  |  |  |  |
|  | Huynh-Feldt | 393.568 | 14.000 | 28.112 |  |  |  |  |  |
|  | Lower-bound | 393.568 | 14.000 | 28.112 |  |  |  |  |  |
| a. Computed using alpha = .05 | |  |  |  |  |  |  |  |  |

Results of Two Way ANOVA for k4

| **Descriptive Statistics** | | | |
| --- | --- | --- | --- |
|  | Mean | Std. Deviation | N |
| K4pr | -7.4101 | .65128 | 15 |
| K4po | -6.5500 | .39461 | 15 |
| K4prCG | -7.2454 | 1.01948 | 15 |
| K4poCG | -7.5027 | 1.12577 | 15 |

| **Multivariate Tests^c^** | | | | | | | | | |
| --- | --- | --- | --- | --- | --- | --- | --- | --- | --- |
| Effect | | Value | F | Hypothesis df | Error df | Sig. | Partial Eta Squared | Noncent. Parameter | Observed Power^b^ |
| groups | Pillai's Trace | .290 | 5.710^a^ | 1.000 | 14.000 | .031 | .290 | 5.710 | .604 |
|  | Wilks' Lambda | .710 | 5.710^a^ | 1.000 | 14.000 | .031 | .290 | 5.710 | .604 |
|  | Hotelling's Trace | .408 | 5.710^a^ | 1.000 | 14.000 | .031 | .290 | 5.710 | .604 |
|  | Roy's Largest Root | .408 | 5.710^a^ | 1.000 | 14.000 | .031 | .290 | 5.710 | .604 |
| time | Pillai's Trace | .188 | 3.246^a^ | 1.000 | 14.000 | .093 | .188 | 3.246 | .389 |
|  | Wilks' Lambda | .812 | 3.246^a^ | 1.000 | 14.000 | .093 | .188 | 3.246 | .389 |
|  | Hotelling's Trace | .232 | 3.246^a^ | 1.000 | 14.000 | .093 | .188 | 3.246 | .389 |
|  | Roy's Largest Root | .232 | 3.246^a^ | 1.000 | 14.000 | .093 | .188 | 3.246 | .389 |
| groups * time | Pillai's Trace | .558 | 17.686^a^ | 1.000 | 14.000 | .001 | .558 | 17.686 | .974 |
|  | Wilks' Lambda | .442 | 17.686^a^ | 1.000 | 14.000 | .001 | .558 | 17.686 | .974 |
|  | Hotelling's Trace | 1.263 | 17.686^a^ | 1.000 | 14.000 | .001 | .558 | 17.686 | .974 |
|  | Roy's Largest Root | 1.263 | 17.686^a^ | 1.000 | 14.000 | .001 | .558 | 17.686 | .974 |
| a. Exact statistic | |  |  |  |  |  |  |  |  |
| b. Computed using alpha = .05 | | |  |  |  |  |  |  |  |
| c. Design: Intercept  Within Subjects Design: groups + time + groups * time | | | |  |  |  |  |  |  |

| **Tests of Within-Subjects Effects** | | | | | | | | | |
| --- | --- | --- | --- | --- | --- | --- | --- | --- | --- |
| Measure:MEASURE_1 | |  |  |  |  |  |  |  |  |
| Source | | Type III Sum of Squares | df | Mean Square | F | Sig. | Partial Eta Squared | Noncent. Parameter | Observed Power^a^ |
| groups | Sphericity Assumed | 2.329 | 1 | 2.329 | 5.710 | .031 | .290 | 5.710 | .604 |
|  | Greenhouse-Geisser | 2.329 | 1.000 | 2.329 | 5.710 | .031 | .290 | 5.710 | .604 |
|  | Huynh-Feldt | 2.329 | 1.000 | 2.329 | 5.710 | .031 | .290 | 5.710 | .604 |
|  | Lower-bound | 2.329 | 1.000 | 2.329 | 5.710 | .031 | .290 | 5.710 | .604 |
| Error(groups) | Sphericity Assumed | 5.709 | 14 | .408 |  |  |  |  |  |
|  | Greenhouse-Geisser | 5.709 | 14.000 | .408 |  |  |  |  |  |
|  | Huynh-Feldt | 5.709 | 14.000 | .408 |  |  |  |  |  |
|  | Lower-bound | 5.709 | 14.000 | .408 |  |  |  |  |  |
| time | Sphericity Assumed | 1.363 | 1 | 1.363 | 3.246 | .093 | .188 | 3.246 | .389 |
|  | Greenhouse-Geisser | 1.363 | 1.000 | 1.363 | 3.246 | .093 | .188 | 3.246 | .389 |
|  | Huynh-Feldt | 1.363 | 1.000 | 1.363 | 3.246 | .093 | .188 | 3.246 | .389 |
|  | Lower-bound | 1.363 | 1.000 | 1.363 | 3.246 | .093 | .188 | 3.246 | .389 |
| Error(time) | Sphericity Assumed | 5.879 | 14 | .420 |  |  |  |  |  |
|  | Greenhouse-Geisser | 5.879 | 14.000 | .420 |  |  |  |  |  |
|  | Huynh-Feldt | 5.879 | 14.000 | .420 |  |  |  |  |  |
|  | Lower-bound | 5.879 | 14.000 | .420 |  |  |  |  |  |
| groups * time | Sphericity Assumed | 4.682 | 1 | 4.682 | 17.686 | .001 | .558 | 17.686 | .974 |
|  | Greenhouse-Geisser | 4.682 | 1.000 | 4.682 | 17.686 | .001 | .558 | 17.686 | .974 |
|  | Huynh-Feldt | 4.682 | 1.000 | 4.682 | 17.686 | .001 | .558 | 17.686 | .974 |
|  | Lower-bound | 4.682 | 1.000 | 4.682 | 17.686 | .001 | .558 | 17.686 | .974 |
| Error(groups*time) | Sphericity Assumed | 3.707 | 14 | .265 |  |  |  |  |  |
|  | Greenhouse-Geisser | 3.707 | 14.000 | .265 |  |  |  |  |  |
|  | Huynh-Feldt | 3.707 | 14.000 | .265 |  |  |  |  |  |
|  | Lower-bound | 3.707 | 14.000 | .265 |  |  |  |  |  |
| a. Computed using alpha = .05 | |  |  |  |  |  |  |  |  |

Results of Two Way ANOVA for k5

| **Descriptive Statistics** | | | |
| --- | --- | --- | --- |
|  | Mean | Std. Deviation | N |
| K5pr | -15.1557 | .84199 | 15 |
| K5po | -13.7967 | .80900 | 15 |
| K5prCG | -14.9473 | 1.63658 | 15 |
| K5poCG | -15.3141 | 1.82989 | 15 |

| **Multivariate Tests^c^** | | | | | | | | | |
| --- | --- | --- | --- | --- | --- | --- | --- | --- | --- |
| Effect | | Value | F | Hypothesis df | Error df | Sig. | Partial Eta Squared | Noncent. Parameter | Observed Power^b^ |
| groups | Pillai's Trace | .226 | 4.099^a^ | 1.000 | 14.000 | .062 | .226 | 4.099 | .470 |
|  | Wilks' Lambda | .774 | 4.099^a^ | 1.000 | 14.000 | .062 | .226 | 4.099 | .470 |
|  | Hotelling's Trace | .293 | 4.099^a^ | 1.000 | 14.000 | .062 | .226 | 4.099 | .470 |
|  | Roy's Largest Root | .293 | 4.099^a^ | 1.000 | 14.000 | .062 | .226 | 4.099 | .470 |
| time | Pillai's Trace | .178 | 3.027^a^ | 1.000 | 14.000 | .104 | .178 | 3.027 | .367 |
|  | Wilks' Lambda | .822 | 3.027^a^ | 1.000 | 14.000 | .104 | .178 | 3.027 | .367 |
|  | Hotelling's Trace | .216 | 3.027^a^ | 1.000 | 14.000 | .104 | .178 | 3.027 | .367 |
|  | Roy's Largest Root | .216 | 3.027^a^ | 1.000 | 14.000 | .104 | .178 | 3.027 | .367 |
| groups * time | Pillai's Trace | .488 | 13.318^a^ | 1.000 | 14.000 | .003 | .488 | 13.318 | .924 |
|  | Wilks' Lambda | .512 | 13.318^a^ | 1.000 | 14.000 | .003 | .488 | 13.318 | .924 |
|  | Hotelling's Trace | .951 | 13.318^a^ | 1.000 | 14.000 | .003 | .488 | 13.318 | .924 |
|  | Roy's Largest Root | .951 | 13.318^a^ | 1.000 | 14.000 | .003 | .488 | 13.318 | .924 |
| a. Exact statistic | |  |  |  |  |  |  |  |  |
| b. Computed using alpha = .05 | | |  |  |  |  |  |  |  |
| c. Design: Intercept  Within Subjects Design: groups + time + groups * time | | | |  |  |  |  |  |  |

| **Tests of Within-Subjects Effects** | | | | | | | | | |
| --- | --- | --- | --- | --- | --- | --- | --- | --- | --- |
| Measure:MEASURE_1 | |  |  |  |  |  |  |  |  |
| Source | | Type III Sum of Squares | df | Mean Square | F | Sig. | Partial Eta Squared | Noncent. Parameter | Observed Power^a^ |
| groups | Sphericity Assumed | 6.426 | 1 | 6.426 | 4.099 | .062 | .226 | 4.099 | .470 |
|  | Greenhouse-Geisser | 6.426 | 1.000 | 6.426 | 4.099 | .062 | .226 | 4.099 | .470 |
|  | Huynh-Feldt | 6.426 | 1.000 | 6.426 | 4.099 | .062 | .226 | 4.099 | .470 |
|  | Lower-bound | 6.426 | 1.000 | 6.426 | 4.099 | .062 | .226 | 4.099 | .470 |
| Error(groups) | Sphericity Assumed | 21.947 | 14 | 1.568 |  |  |  |  |  |
|  | Greenhouse-Geisser | 21.947 | 14.000 | 1.568 |  |  |  |  |  |
|  | Huynh-Feldt | 21.947 | 14.000 | 1.568 |  |  |  |  |  |
|  | Lower-bound | 21.947 | 14.000 | 1.568 |  |  |  |  |  |
| time | Sphericity Assumed | 3.692 | 1 | 3.692 | 3.027 | .104 | .178 | 3.027 | .367 |
|  | Greenhouse-Geisser | 3.692 | 1.000 | 3.692 | 3.027 | .104 | .178 | 3.027 | .367 |
|  | Huynh-Feldt | 3.692 | 1.000 | 3.692 | 3.027 | .104 | .178 | 3.027 | .367 |
|  | Lower-bound | 3.692 | 1.000 | 3.692 | 3.027 | .104 | .178 | 3.027 | .367 |
| Error(time) | Sphericity Assumed | 17.076 | 14 | 1.220 |  |  |  |  |  |
|  | Greenhouse-Geisser | 17.076 | 14.000 | 1.220 |  |  |  |  |  |
|  | Huynh-Feldt | 17.076 | 14.000 | 1.220 |  |  |  |  |  |
|  | Lower-bound | 17.076 | 14.000 | 1.220 |  |  |  |  |  |
| groups * time | Sphericity Assumed | 11.168 | 1 | 11.168 | 13.318 | .003 | .488 | 13.318 | .924 |
|  | Greenhouse-Geisser | 11.168 | 1.000 | 11.168 | 13.318 | .003 | .488 | 13.318 | .924 |
|  | Huynh-Feldt | 11.168 | 1.000 | 11.168 | 13.318 | .003 | .488 | 13.318 | .924 |
|  | Lower-bound | 11.168 | 1.000 | 11.168 | 13.318 | .003 | .488 | 13.318 | .924 |
| Error(groups*time) | Sphericity Assumed | 11.739 | 14 | .839 |  |  |  |  |  |
|  | Greenhouse-Geisser | 11.739 | 14.000 | .839 |  |  |  |  |  |
|  | Huynh-Feldt | 11.739 | 14.000 | .839 |  |  |  |  |  |
|  | Lower-bound | 11.739 | 14.000 | .839 |  |  |  |  |  |
| a. Computed using alpha = .05 | |  |  |  |  |  |  |  |  |

Results of Two Way ANOVA for k6

| **Descriptive Statistics** | | | |
| --- | --- | --- | --- |
|  | Mean | Std. Deviation | N |
| K6pr | -12.6826 | .89612 | 15 |
| K6po | -10.6554 | .77657 | 15 |
| K6prCG | -12.8224 | 1.12196 | 15 |
| K6poCG | -12.1744 | 1.38707 | 15 |

| **Multivariate Tests^c^** | | | | | | | | | |
| --- | --- | --- | --- | --- | --- | --- | --- | --- | --- |
| Effect | | Value | F | Hypothesis df | Error df | Sig. | Partial Eta Squared | Noncent. Parameter | Observed Power^b^ |
| groups | Pillai's Trace | .331 | 6.939^a^ | 1.000 | 14.000 | .020 | .331 | 6.939 | .688 |
|  | Wilks' Lambda | .669 | 6.939^a^ | 1.000 | 14.000 | .020 | .331 | 6.939 | .688 |
|  | Hotelling's Trace | .496 | 6.939^a^ | 1.000 | 14.000 | .020 | .331 | 6.939 | .688 |
|  | Roy's Largest Root | .496 | 6.939^a^ | 1.000 | 14.000 | .020 | .331 | 6.939 | .688 |
| time | Pillai's Trace | .672 | 28.734^a^ | 1.000 | 14.000 | .000 | .672 | 28.734 | .999 |
|  | Wilks' Lambda | .328 | 28.734^a^ | 1.000 | 14.000 | .000 | .672 | 28.734 | .999 |
|  | Hotelling's Trace | 2.052 | 28.734^a^ | 1.000 | 14.000 | .000 | .672 | 28.734 | .999 |
|  | Roy's Largest Root | 2.052 | 28.734^a^ | 1.000 | 14.000 | .000 | .672 | 28.734 | .999 |
| groups * time | Pillai's Trace | .405 | 9.547^a^ | 1.000 | 14.000 | .008 | .405 | 9.547 | .819 |
|  | Wilks' Lambda | .595 | 9.547^a^ | 1.000 | 14.000 | .008 | .405 | 9.547 | .819 |
|  | Hotelling's Trace | .682 | 9.547^a^ | 1.000 | 14.000 | .008 | .405 | 9.547 | .819 |
|  | Roy's Largest Root | .682 | 9.547^a^ | 1.000 | 14.000 | .008 | .405 | 9.547 | .819 |
| a. Exact statistic | |  |  |  |  |  |  |  |  |
| b. Computed using alpha = .05 | | |  |  |  |  |  |  |  |
| c. Design: Intercept  Within Subjects Design: groups + time + groups * time | | | |  |  |  |  |  |  |

| **Tests of Within-Subjects Effects** | | | | | | | | | |
| --- | --- | --- | --- | --- | --- | --- | --- | --- | --- |
| Measure:MEASURE_1 | |  |  |  |  |  |  |  |  |
| Source | | Type III Sum of Squares | df | Mean Square | F | Sig. | Partial Eta Squared | Noncent. Parameter | Observed Power^a^ |
| groups | Sphericity Assumed | 10.318 | 1 | 10.318 | 6.939 | .020 | .331 | 6.939 | .688 |
|  | Greenhouse-Geisser | 10.318 | 1.000 | 10.318 | 6.939 | .020 | .331 | 6.939 | .688 |
|  | Huynh-Feldt | 10.318 | 1.000 | 10.318 | 6.939 | .020 | .331 | 6.939 | .688 |
|  | Lower-bound | 10.318 | 1.000 | 10.318 | 6.939 | .020 | .331 | 6.939 | .688 |
| Error(groups) | Sphericity Assumed | 20.818 | 14 | 1.487 |  |  |  |  |  |
|  | Greenhouse-Geisser | 20.818 | 14.000 | 1.487 |  |  |  |  |  |
|  | Huynh-Feldt | 20.818 | 14.000 | 1.487 |  |  |  |  |  |
|  | Lower-bound | 20.818 | 14.000 | 1.487 |  |  |  |  |  |
| time | Sphericity Assumed | 26.839 | 1 | 26.839 | 28.734 | .000 | .672 | 28.734 | .999 |
|  | Greenhouse-Geisser | 26.839 | 1.000 | 26.839 | 28.734 | .000 | .672 | 28.734 | .999 |
|  | Huynh-Feldt | 26.839 | 1.000 | 26.839 | 28.734 | .000 | .672 | 28.734 | .999 |
|  | Lower-bound | 26.839 | 1.000 | 26.839 | 28.734 | .000 | .672 | 28.734 | .999 |
| Error(time) | Sphericity Assumed | 13.077 | 14 | .934 |  |  |  |  |  |
|  | Greenhouse-Geisser | 13.077 | 14.000 | .934 |  |  |  |  |  |
|  | Huynh-Feldt | 13.077 | 14.000 | .934 |  |  |  |  |  |
|  | Lower-bound | 13.077 | 14.000 | .934 |  |  |  |  |  |
| groups * time | Sphericity Assumed | 7.134 | 1 | 7.134 | 9.547 | .008 | .405 | 9.547 | .819 |
|  | Greenhouse-Geisser | 7.134 | 1.000 | 7.134 | 9.547 | .008 | .405 | 9.547 | .819 |
|  | Huynh-Feldt | 7.134 | 1.000 | 7.134 | 9.547 | .008 | .405 | 9.547 | .819 |
|  | Lower-bound | 7.134 | 1.000 | 7.134 | 9.547 | .008 | .405 | 9.547 | .819 |
| Error(groups*time) | Sphericity Assumed | 10.462 | 14 | .747 |  |  |  |  |  |
|  | Greenhouse-Geisser | 10.462 | 14.000 | .747 |  |  |  |  |  |
|  | Huynh-Feldt | 10.462 | 14.000 | .747 |  |  |  |  |  |
|  | Lower-bound | 10.462 | 14.000 | .747 |  |  |  |  |  |
| a. Computed using alpha = .05 | |  |  |  |  |  |  |  |  |

Results of Two Way ANOVA for k7

| **Descriptive Statistics** | | | |
| --- | --- | --- | --- |
|  | Mean | Std. Deviation | N |
| K7pr | 8.1103 | 2.57001 | 15 |
| K7po | 4.7904 | .66519 | 15 |
| K7prCG | 8.1215 | 2.90340 | 15 |
| K7poCG | 7.7716 | 2.16753 | 15 |

| **Multivariate Tests^c^** | | | | | | | | | |
| --- | --- | --- | --- | --- | --- | --- | --- | --- | --- |
| Effect | | Value | F | Hypothesis df | Error df | Sig. | Partial Eta Squared | Noncent. Parameter | Observed Power^b^ |
| groups | Pillai's Trace | .345 | 7.377^a^ | 1.000 | 14.000 | .017 | .345 | 7.377 | .714 |
|  | Wilks' Lambda | .655 | 7.377^a^ | 1.000 | 14.000 | .017 | .345 | 7.377 | .714 |
|  | Hotelling's Trace | .527 | 7.377^a^ | 1.000 | 14.000 | .017 | .345 | 7.377 | .714 |
|  | Roy's Largest Root | .527 | 7.377^a^ | 1.000 | 14.000 | .017 | .345 | 7.377 | .714 |
| time | Pillai's Trace | .449 | 11.394^a^ | 1.000 | 14.000 | .005 | .449 | 11.394 | .880 |
|  | Wilks' Lambda | .551 | 11.394^a^ | 1.000 | 14.000 | .005 | .449 | 11.394 | .880 |
|  | Hotelling's Trace | .814 | 11.394^a^ | 1.000 | 14.000 | .005 | .449 | 11.394 | .880 |
|  | Roy's Largest Root | .814 | 11.394^a^ | 1.000 | 14.000 | .005 | .449 | 11.394 | .880 |
| groups * time | Pillai's Trace | .350 | 7.541^a^ | 1.000 | 14.000 | .016 | .350 | 7.541 | .724 |
|  | Wilks' Lambda | .650 | 7.541^a^ | 1.000 | 14.000 | .016 | .350 | 7.541 | .724 |
|  | Hotelling's Trace | .539 | 7.541^a^ | 1.000 | 14.000 | .016 | .350 | 7.541 | .724 |
|  | Roy's Largest Root | .539 | 7.541^a^ | 1.000 | 14.000 | .016 | .350 | 7.541 | .724 |
| a. Exact statistic | |  |  |  |  |  |  |  |  |
| b. Computed using alpha = .05 | | |  |  |  |  |  |  |  |
| c. Design: Intercept  Within Subjects Design: groups + time + groups * time | | | |  |  |  |  |  |  |

| **Tests of Within-Subjects Effects** | | | | | | | | | |
| --- | --- | --- | --- | --- | --- | --- | --- | --- | --- |
| Measure:MEASURE_1 | |  |  |  |  |  |  |  |  |
| Source | | Type III Sum of Squares | df | Mean Square | F | Sig. | Partial Eta Squared | Noncent. Parameter | Observed Power^a^ |
| groups | Sphericity Assumed | 33.582 | 1 | 33.582 | 7.377 | .017 | .345 | 7.377 | .714 |
|  | Greenhouse-Geisser | 33.582 | 1.000 | 33.582 | 7.377 | .017 | .345 | 7.377 | .714 |
|  | Huynh-Feldt | 33.582 | 1.000 | 33.582 | 7.377 | .017 | .345 | 7.377 | .714 |
|  | Lower-bound | 33.582 | 1.000 | 33.582 | 7.377 | .017 | .345 | 7.377 | .714 |
| Error(groups) | Sphericity Assumed | 63.735 | 14 | 4.552 |  |  |  |  |  |
|  | Greenhouse-Geisser | 63.735 | 14.000 | 4.552 |  |  |  |  |  |
|  | Huynh-Feldt | 63.735 | 14.000 | 4.552 |  |  |  |  |  |
|  | Lower-bound | 63.735 | 14.000 | 4.552 |  |  |  |  |  |
| time | Sphericity Assumed | 50.502 | 1 | 50.502 | 11.394 | .005 | .449 | 11.394 | .880 |
|  | Greenhouse-Geisser | 50.502 | 1.000 | 50.502 | 11.394 | .005 | .449 | 11.394 | .880 |
|  | Huynh-Feldt | 50.502 | 1.000 | 50.502 | 11.394 | .005 | .449 | 11.394 | .880 |
|  | Lower-bound | 50.502 | 1.000 | 50.502 | 11.394 | .005 | .449 | 11.394 | .880 |
| Error(time) | Sphericity Assumed | 62.050 | 14 | 4.432 |  |  |  |  |  |
|  | Greenhouse-Geisser | 62.050 | 14.000 | 4.432 |  |  |  |  |  |
|  | Huynh-Feldt | 62.050 | 14.000 | 4.432 |  |  |  |  |  |
|  | Lower-bound | 62.050 | 14.000 | 4.432 |  |  |  |  |  |
| groups * time | Sphericity Assumed | 33.078 | 1 | 33.078 | 7.541 | .016 | .350 | 7.541 | .724 |
|  | Greenhouse-Geisser | 33.078 | 1.000 | 33.078 | 7.541 | .016 | .350 | 7.541 | .724 |
|  | Huynh-Feldt | 33.078 | 1.000 | 33.078 | 7.541 | .016 | .350 | 7.541 | .724 |
|  | Lower-bound | 33.078 | 1.000 | 33.078 | 7.541 | .016 | .350 | 7.541 | .724 |
| Error(groups*time) | Sphericity Assumed | 61.413 | 14 | 4.387 |  |  |  |  |  |
|  | Greenhouse-Geisser | 61.413 | 14.000 | 4.387 |  |  |  |  |  |
|  | Huynh-Feldt | 61.413 | 14.000 | 4.387 |  |  |  |  |  |
|  | Lower-bound | 61.413 | 14.000 | 4.387 |  |  |  |  |  |
| a. Computed using alpha = .05 | |  |  |  |  |  |  |  |  |

Results of Two Way ANOVA for H1

| **Descriptive Statistics** | | | |
| --- | --- | --- | --- |
|  | Mean | Std. Deviation | N |
| H1pr | -10.0013 | 3.58882 | 15 |
| H1po | -13.2449 | 5.27954 | 15 |
| H1prCG | -9.7568 | 3.52969 | 15 |
| H1poCG | -9.5421 | 3.38355 | 15 |

| **Multivariate Tests^c^** | | | | | | | | | |
| --- | --- | --- | --- | --- | --- | --- | --- | --- | --- |
| Effect | | Value | F | Hypothesis df | Error df | Sig. | Partial Eta Squared | Noncent. Parameter | Observed Power^b^ |
| groups | Pillai's Trace | .176 | 2.984^a^ | 1.000 | 14.000 | .106 | .176 | 2.984 | .363 |
|  | Wilks' Lambda | .824 | 2.984^a^ | 1.000 | 14.000 | .106 | .176 | 2.984 | .363 |
|  | Hotelling's Trace | .213 | 2.984^a^ | 1.000 | 14.000 | .106 | .176 | 2.984 | .363 |
|  | Roy's Largest Root | .213 | 2.984^a^ | 1.000 | 14.000 | .106 | .176 | 2.984 | .363 |
| time | Pillai's Trace | .179 | 3.043^a^ | 1.000 | 14.000 | .103 | .179 | 3.043 | .369 |
|  | Wilks' Lambda | .821 | 3.043^a^ | 1.000 | 14.000 | .103 | .179 | 3.043 | .369 |
|  | Hotelling's Trace | .217 | 3.043^a^ | 1.000 | 14.000 | .103 | .179 | 3.043 | .369 |
|  | Roy's Largest Root | .217 | 3.043^a^ | 1.000 | 14.000 | .103 | .179 | 3.043 | .369 |
| groups * time | Pillai's Trace | .301 | 6.018^a^ | 1.000 | 14.000 | .028 | .301 | 6.018 | .627 |
|  | Wilks' Lambda | .699 | 6.018^a^ | 1.000 | 14.000 | .028 | .301 | 6.018 | .627 |
|  | Hotelling's Trace | .430 | 6.018^a^ | 1.000 | 14.000 | .028 | .301 | 6.018 | .627 |
|  | Roy's Largest Root | .430 | 6.018^a^ | 1.000 | 14.000 | .028 | .301 | 6.018 | .627 |
| a. Exact statistic | |  |  |  |  |  |  |  |  |
| b. Computed using alpha = .05 | | |  |  |  |  |  |  |  |
| c. Design: Intercept  Within Subjects Design: groups + time + groups * time | | | |  |  |  |  |  |  |

| **Tests of Within-Subjects Effects** | | | | | | | | | |
| --- | --- | --- | --- | --- | --- | --- | --- | --- | --- |
| Measure:MEASURE_1 | |  |  |  |  |  |  |  |  |
| Source | | Type III Sum of Squares | df | Mean Square | F | Sig. | Partial Eta Squared | Noncent. Parameter | Observed Power^a^ |
| groups | Sphericity Assumed | 58.428 | 1 | 58.428 | 2.984 | .106 | .176 | 2.984 | .363 |
|  | Greenhouse-Geisser | 58.428 | 1.000 | 58.428 | 2.984 | .106 | .176 | 2.984 | .363 |
|  | Huynh-Feldt | 58.428 | 1.000 | 58.428 | 2.984 | .106 | .176 | 2.984 | .363 |
|  | Lower-bound | 58.428 | 1.000 | 58.428 | 2.984 | .106 | .176 | 2.984 | .363 |
| Error(groups) | Sphericity Assumed | 274.138 | 14 | 19.581 |  |  |  |  |  |
|  | Greenhouse-Geisser | 274.138 | 14.000 | 19.581 |  |  |  |  |  |
|  | Huynh-Feldt | 274.138 | 14.000 | 19.581 |  |  |  |  |  |
|  | Lower-bound | 274.138 | 14.000 | 19.581 |  |  |  |  |  |
| time | Sphericity Assumed | 34.401 | 1 | 34.401 | 3.043 | .103 | .179 | 3.043 | .369 |
|  | Greenhouse-Geisser | 34.401 | 1.000 | 34.401 | 3.043 | .103 | .179 | 3.043 | .369 |
|  | Huynh-Feldt | 34.401 | 1.000 | 34.401 | 3.043 | .103 | .179 | 3.043 | .369 |
|  | Lower-bound | 34.401 | 1.000 | 34.401 | 3.043 | .103 | .179 | 3.043 | .369 |
| Error(time) | Sphericity Assumed | 158.290 | 14 | 11.306 |  |  |  |  |  |
|  | Greenhouse-Geisser | 158.290 | 14.000 | 11.306 |  |  |  |  |  |
|  | Huynh-Feldt | 158.290 | 14.000 | 11.306 |  |  |  |  |  |
|  | Lower-bound | 158.290 | 14.000 | 11.306 |  |  |  |  |  |
| groups * time | Sphericity Assumed | 44.850 | 1 | 44.850 | 6.018 | .028 | .301 | 6.018 | .627 |
|  | Greenhouse-Geisser | 44.850 | 1.000 | 44.850 | 6.018 | .028 | .301 | 6.018 | .627 |
|  | Huynh-Feldt | 44.850 | 1.000 | 44.850 | 6.018 | .028 | .301 | 6.018 | .627 |
|  | Lower-bound | 44.850 | 1.000 | 44.850 | 6.018 | .028 | .301 | 6.018 | .627 |
| Error(groups*time) | Sphericity Assumed | 104.332 | 14 | 7.452 |  |  |  |  |  |
|  | Greenhouse-Geisser | 104.332 | 14.000 | 7.452 |  |  |  |  |  |
|  | Huynh-Feldt | 104.332 | 14.000 | 7.452 |  |  |  |  |  |
|  | Lower-bound | 104.332 | 14.000 | 7.452 |  |  |  |  |  |
| a. Computed using alpha = .05 | |  |  |  |  |  |  |  |  |

Results of Two Way ANOVA for H2

| **Descriptive Statistics** | | | |
| --- | --- | --- | --- |
|  | Mean | Std. Deviation | N |
| H2pr | 3.5936 | 1.03553 | 15 |
| H2po | 3.1291 | 2.03887 | 15 |
| H2prCG | 3.5492 | 1.20396 | 15 |
| H2poCG | 3.9210 | 1.19315 | 15 |

| **Multivariate Tests^c^** | | | | | | | | | |
| --- | --- | --- | --- | --- | --- | --- | --- | --- | --- |
| Effect | | Value | F | Hypothesis df | Error df | Sig. | Partial Eta Squared | Noncent. Parameter | Observed Power^b^ |
| groups | Pillai's Trace | .123 | 1.964^a^ | 1.000 | 14.000 | .183 | .123 | 1.964 | .257 |
|  | Wilks' Lambda | .877 | 1.964^a^ | 1.000 | 14.000 | .183 | .123 | 1.964 | .257 |
|  | Hotelling's Trace | .140 | 1.964^a^ | 1.000 | 14.000 | .183 | .123 | 1.964 | .257 |
|  | Roy's Largest Root | .140 | 1.964^a^ | 1.000 | 14.000 | .183 | .123 | 1.964 | .257 |
| time | Pillai's Trace | .002 | .022^a^ | 1.000 | 14.000 | .884 | .002 | .022 | .052 |
|  | Wilks' Lambda | .998 | .022^a^ | 1.000 | 14.000 | .884 | .002 | .022 | .052 |
|  | Hotelling's Trace | .002 | .022^a^ | 1.000 | 14.000 | .884 | .002 | .022 | .052 |
|  | Roy's Largest Root | .002 | .022^a^ | 1.000 | 14.000 | .884 | .002 | .022 | .052 |
| groups * time | Pillai's Trace | .107 | 1.683^a^ | 1.000 | 14.000 | .215 | .107 | 1.683 | .227 |
|  | Wilks' Lambda | .893 | 1.683^a^ | 1.000 | 14.000 | .215 | .107 | 1.683 | .227 |
|  | Hotelling's Trace | .120 | 1.683^a^ | 1.000 | 14.000 | .215 | .107 | 1.683 | .227 |
|  | Roy's Largest Root | .120 | 1.683^a^ | 1.000 | 14.000 | .215 | .107 | 1.683 | .227 |
| a. Exact statistic | |  |  |  |  |  |  |  |  |
| b. Computed using alpha = .05 | | |  |  |  |  |  |  |  |
| c. Design: Intercept  Within Subjects Design: groups + time + groups * time | | | |  |  |  |  |  |  |

| **Tests of Within-Subjects Effects** | | | | | | | | | |
| --- | --- | --- | --- | --- | --- | --- | --- | --- | --- |
| Measure:MEASURE_1 | |  |  |  |  |  |  |  |  |
| Source | | Type III Sum of Squares | df | Mean Square | F | Sig. | Partial Eta Squared | Noncent. Parameter | Observed Power^a^ |
| groups | Sphericity Assumed | 2.095 | 1 | 2.095 | 1.964 | .183 | .123 | 1.964 | .257 |
|  | Greenhouse-Geisser | 2.095 | 1.000 | 2.095 | 1.964 | .183 | .123 | 1.964 | .257 |
|  | Huynh-Feldt | 2.095 | 1.000 | 2.095 | 1.964 | .183 | .123 | 1.964 | .257 |
|  | Lower-bound | 2.095 | 1.000 | 2.095 | 1.964 | .183 | .123 | 1.964 | .257 |
| Error(groups) | Sphericity Assumed | 14.935 | 14 | 1.067 |  |  |  |  |  |
|  | Greenhouse-Geisser | 14.935 | 14.000 | 1.067 |  |  |  |  |  |
|  | Huynh-Feldt | 14.935 | 14.000 | 1.067 |  |  |  |  |  |
|  | Lower-bound | 14.935 | 14.000 | 1.067 |  |  |  |  |  |
| time | Sphericity Assumed | .032 | 1 | .032 | .022 | .884 | .002 | .022 | .052 |
|  | Greenhouse-Geisser | .032 | 1.000 | .032 | .022 | .884 | .002 | .022 | .052 |
|  | Huynh-Feldt | .032 | 1.000 | .032 | .022 | .884 | .002 | .022 | .052 |
|  | Lower-bound | .032 | 1.000 | .032 | .022 | .884 | .002 | .022 | .052 |
| Error(time) | Sphericity Assumed | 20.358 | 14 | 1.454 |  |  |  |  |  |
|  | Greenhouse-Geisser | 20.358 | 14.000 | 1.454 |  |  |  |  |  |
|  | Huynh-Feldt | 20.358 | 14.000 | 1.454 |  |  |  |  |  |
|  | Lower-bound | 20.358 | 14.000 | 1.454 |  |  |  |  |  |
| groups * time | Sphericity Assumed | 2.623 | 1 | 2.623 | 1.683 | .215 | .107 | 1.683 | .227 |
|  | Greenhouse-Geisser | 2.623 | 1.000 | 2.623 | 1.683 | .215 | .107 | 1.683 | .227 |
|  | Huynh-Feldt | 2.623 | 1.000 | 2.623 | 1.683 | .215 | .107 | 1.683 | .227 |
|  | Lower-bound | 2.623 | 1.000 | 2.623 | 1.683 | .215 | .107 | 1.683 | .227 |
| Error(groups*time) | Sphericity Assumed | 21.823 | 14 | 1.559 |  |  |  |  |  |
|  | Greenhouse-Geisser | 21.823 | 14.000 | 1.559 |  |  |  |  |  |
|  | Huynh-Feldt | 21.823 | 14.000 | 1.559 |  |  |  |  |  |
|  | Lower-bound | 21.823 | 14.000 | 1.559 |  |  |  |  |  |
| a. Computed using alpha = .05 | |  |  |  |  |  |  |  |  |

Results of Two Way ANOVA for H3

| **Descriptive Statistics** | | | |
| --- | --- | --- | --- |
|  | Mean | Std. Deviation | N |
| H3pr | -18.6681 | .99064 | 15 |
| H3po | -16.7564 | .58757 | 15 |
| H3prCG | -18.7107 | 1.54908 | 15 |
| H3poCG | -19.0175 | 3.44842 | 15 |

| **Multivariate Tests^c^** | | | | | | | | | |
| --- | --- | --- | --- | --- | --- | --- | --- | --- | --- |
| Effect | | Value | F | Hypothesis df | Error df | Sig. | Partial Eta Squared | Noncent. Parameter | Observed Power^b^ |
| groups | Pillai's Trace | .240 | 4.418^a^ | 1.000 | 14.000 | .054 | .240 | 4.418 | .499 |
|  | Wilks' Lambda | .760 | 4.418^a^ | 1.000 | 14.000 | .054 | .240 | 4.418 | .499 |
|  | Hotelling's Trace | .316 | 4.418^a^ | 1.000 | 14.000 | .054 | .240 | 4.418 | .499 |
|  | Roy's Largest Root | .316 | 4.418^a^ | 1.000 | 14.000 | .054 | .240 | 4.418 | .499 |
| time | Pillai's Trace | .232 | 4.224^a^ | 1.000 | 14.000 | .059 | .232 | 4.224 | .482 |
|  | Wilks' Lambda | .768 | 4.224^a^ | 1.000 | 14.000 | .059 | .232 | 4.224 | .482 |
|  | Hotelling's Trace | .302 | 4.224^a^ | 1.000 | 14.000 | .059 | .232 | 4.224 | .482 |
|  | Roy's Largest Root | .302 | 4.224^a^ | 1.000 | 14.000 | .059 | .232 | 4.224 | .482 |
| groups * time | Pillai's Trace | .264 | 5.015^a^ | 1.000 | 14.000 | .042 | .264 | 5.015 | .550 |
|  | Wilks' Lambda | .736 | 5.015^a^ | 1.000 | 14.000 | .042 | .264 | 5.015 | .550 |
|  | Hotelling's Trace | .358 | 5.015^a^ | 1.000 | 14.000 | .042 | .264 | 5.015 | .550 |
|  | Roy's Largest Root | .358 | 5.015^a^ | 1.000 | 14.000 | .042 | .264 | 5.015 | .550 |
| a. Exact statistic | |  |  |  |  |  |  |  |  |
| b. Computed using alpha = .05 | | |  |  |  |  |  |  |  |
| c. Design: Intercept  Within Subjects Design: groups + time + groups * time | | | |  |  |  |  |  |  |

| **Tests of Within-Subjects Effects** | | | | | | | | | |
| --- | --- | --- | --- | --- | --- | --- | --- | --- | --- |
| Measure:MEASURE_1 | |  |  |  |  |  |  |  |  |
| Source | | Type III Sum of Squares | df | Mean Square | F | Sig. | Partial Eta Squared | Noncent. Parameter | Observed Power^a^ |
| groups | Sphericity Assumed | 19.902 | 1 | 19.902 | 4.418 | .054 | .240 | 4.418 | .499 |
|  | Greenhouse-Geisser | 19.902 | 1.000 | 19.902 | 4.418 | .054 | .240 | 4.418 | .499 |
|  | Huynh-Feldt | 19.902 | 1.000 | 19.902 | 4.418 | .054 | .240 | 4.418 | .499 |
|  | Lower-bound | 19.902 | 1.000 | 19.902 | 4.418 | .054 | .240 | 4.418 | .499 |
| Error(groups) | Sphericity Assumed | 63.068 | 14 | 4.505 |  |  |  |  |  |
|  | Greenhouse-Geisser | 63.068 | 14.000 | 4.505 |  |  |  |  |  |
|  | Huynh-Feldt | 63.068 | 14.000 | 4.505 |  |  |  |  |  |
|  | Lower-bound | 63.068 | 14.000 | 4.505 |  |  |  |  |  |
| time | Sphericity Assumed | 9.658 | 1 | 9.658 | 4.224 | .059 | .232 | 4.224 | .482 |
|  | Greenhouse-Geisser | 9.658 | 1.000 | 9.658 | 4.224 | .059 | .232 | 4.224 | .482 |
|  | Huynh-Feldt | 9.658 | 1.000 | 9.658 | 4.224 | .059 | .232 | 4.224 | .482 |
|  | Lower-bound | 9.658 | 1.000 | 9.658 | 4.224 | .059 | .232 | 4.224 | .482 |
| Error(time) | Sphericity Assumed | 32.012 | 14 | 2.287 |  |  |  |  |  |
|  | Greenhouse-Geisser | 32.012 | 14.000 | 2.287 |  |  |  |  |  |
|  | Huynh-Feldt | 32.012 | 14.000 | 2.287 |  |  |  |  |  |
|  | Lower-bound | 32.012 | 14.000 | 2.287 |  |  |  |  |  |
| groups * time | Sphericity Assumed | 18.458 | 1 | 18.458 | 5.015 | .042 | .264 | 5.015 | .550 |
|  | Greenhouse-Geisser | 18.458 | 1.000 | 18.458 | 5.015 | .042 | .264 | 5.015 | .550 |
|  | Huynh-Feldt | 18.458 | 1.000 | 18.458 | 5.015 | .042 | .264 | 5.015 | .550 |
|  | Lower-bound | 18.458 | 1.000 | 18.458 | 5.015 | .042 | .264 | 5.015 | .550 |
| Error(groups*time) | Sphericity Assumed | 51.532 | 14 | 3.681 |  |  |  |  |  |
|  | Greenhouse-Geisser | 51.532 | 14.000 | 3.681 |  |  |  |  |  |
|  | Huynh-Feldt | 51.532 | 14.000 | 3.681 |  |  |  |  |  |
|  | Lower-bound | 51.532 | 14.000 | 3.681 |  |  |  |  |  |
| a. Computed using alpha = .05 | |  |  |  |  |  |  |  |  |

Results of Two Way ANOVA for FZ_HC_

| **Descriptive Statistics** | | | |
| --- | --- | --- | --- |
|  | Mean | Std. Deviation | N |
| pre-test | 1.1538E2 | 10.33528 | 15 |
| post-test | 1.0626E2 | 6.64783 | 15 |
| FZhcPrCG | 1.1396E2 | 10.13834 | 15 |
| FZhcPoCG | 1.1374E2 | 11.44713 | 15 |

| **Multivariate Tests^c^** | | | | | | | | | |
| --- | --- | --- | --- | --- | --- | --- | --- | --- | --- |
| Effect | | Value | F | Hypothesis df | Error df | Sig. | Partial Eta Squared | Noncent. Parameter | Observed Power^b^ |
| groups | Pillai's Trace | .056 | .823^a^ | 1.000 | 14.000 | .380 | .056 | .823 | .135 |
|  | Wilks' Lambda | .944 | .823^a^ | 1.000 | 14.000 | .380 | .056 | .823 | .135 |
|  | Hotelling's Trace | .059 | .823^a^ | 1.000 | 14.000 | .380 | .056 | .823 | .135 |
|  | Roy's Largest Root | .059 | .823^a^ | 1.000 | 14.000 | .380 | .056 | .823 | .135 |
| time | Pillai's Trace | .278 | 5.397^a^ | 1.000 | 14.000 | .036 | .278 | 5.397 | .580 |
|  | Wilks' Lambda | .722 | 5.397^a^ | 1.000 | 14.000 | .036 | .278 | 5.397 | .580 |
|  | Hotelling's Trace | .385 | 5.397^a^ | 1.000 | 14.000 | .036 | .278 | 5.397 | .580 |
|  | Roy's Largest Root | .385 | 5.397^a^ | 1.000 | 14.000 | .036 | .278 | 5.397 | .580 |
| groups * time | Pillai's Trace | .343 | 7.309^a^ | 1.000 | 14.000 | .017 | .343 | 7.309 | .711 |
|  | Wilks' Lambda | .657 | 7.309^a^ | 1.000 | 14.000 | .017 | .343 | 7.309 | .711 |
|  | Hotelling's Trace | .522 | 7.309^a^ | 1.000 | 14.000 | .017 | .343 | 7.309 | .711 |
|  | Roy's Largest Root | .522 | 7.309^a^ | 1.000 | 14.000 | .017 | .343 | 7.309 | .711 |
| a. Exact statistic | |  |  |  |  |  |  |  |  |
| b. Computed using alpha = .05 | | |  |  |  |  |  |  |  |
| c. Design: Intercept  Within Subjects Design: groups + time + groups * time | | | |  |  |  |  |  |  |

| **Tests of Within-Subjects Effects** | | | | | | | | | |
| --- | --- | --- | --- | --- | --- | --- | --- | --- | --- |
| Measure:MEASURE_1 | |  |  |  |  |  |  |  |  |
| Source | | Type III Sum of Squares | df | Mean Square | F | Sig. | Partial Eta Squared | Noncent. Parameter | Observed Power^a^ |
| groups | Sphericity Assumed | 138.107 | 1 | 138.107 | .823 | .380 | .056 | .823 | .135 |
|  | Greenhouse-Geisser | 138.107 | 1.000 | 138.107 | .823 | .380 | .056 | .823 | .135 |
|  | Huynh-Feldt | 138.107 | 1.000 | 138.107 | .823 | .380 | .056 | .823 | .135 |
|  | Lower-bound | 138.107 | 1.000 | 138.107 | .823 | .380 | .056 | .823 | .135 |
| Error(groups) | Sphericity Assumed | 2348.979 | 14 | 167.784 |  |  |  |  |  |
|  | Greenhouse-Geisser | 2348.979 | 14.000 | 167.784 |  |  |  |  |  |
|  | Huynh-Feldt | 2348.979 | 14.000 | 167.784 |  |  |  |  |  |
|  | Lower-bound | 2348.979 | 14.000 | 167.784 |  |  |  |  |  |
| time | Sphericity Assumed | 326.690 | 1 | 326.690 | 5.397 | .036 | .278 | 5.397 | .580 |
|  | Greenhouse-Geisser | 326.690 | 1.000 | 326.690 | 5.397 | .036 | .278 | 5.397 | .580 |
|  | Huynh-Feldt | 326.690 | 1.000 | 326.690 | 5.397 | .036 | .278 | 5.397 | .580 |
|  | Lower-bound | 326.690 | 1.000 | 326.690 | 5.397 | .036 | .278 | 5.397 | .580 |
| Error(time) | Sphericity Assumed | 847.518 | 14 | 60.537 |  |  |  |  |  |
|  | Greenhouse-Geisser | 847.518 | 14.000 | 60.537 |  |  |  |  |  |
|  | Huynh-Feldt | 847.518 | 14.000 | 60.537 |  |  |  |  |  |
|  | Lower-bound | 847.518 | 14.000 | 60.537 |  |  |  |  |  |
| groups * time | Sphericity Assumed | 297.371 | 1 | 297.371 | 7.309 | .017 | .343 | 7.309 | .711 |
|  | Greenhouse-Geisser | 297.371 | 1.000 | 297.371 | 7.309 | .017 | .343 | 7.309 | .711 |
|  | Huynh-Feldt | 297.371 | 1.000 | 297.371 | 7.309 | .017 | .343 | 7.309 | .711 |
|  | Lower-bound | 297.371 | 1.000 | 297.371 | 7.309 | .017 | .343 | 7.309 | .711 |
| Error(groups*time) | Sphericity Assumed | 569.565 | 14 | 40.683 |  |  |  |  |  |
|  | Greenhouse-Geisser | 569.565 | 14.000 | 40.683 |  |  |  |  |  |
|  | Huynh-Feldt | 569.565 | 14.000 | 40.683 |  |  |  |  |  |
|  | Lower-bound | 569.565 | 14.000 | 40.683 |  |  |  |  |  |
| a. Computed using alpha = .05 | |  |  |  |  |  |  |  |  |

Results of Two Way ANOVA for FZ_PO_

| **Descriptive Statistics** | | | |
| --- | --- | --- | --- |
|  | Mean | Std. Deviation | N |
| FZpoPr | 1.0855E2 | 9.28218 | 15 |
| FZpoPo | 1.0965E2 | 6.25822 | 15 |
| FZpoPrCG | 1.1310E2 | 6.14350 | 15 |
| FZpoPoCG | 1.1234E2 | 7.27189 | 15 |

| **Multivariate Tests^c^** | | | | | | | | | |
| --- | --- | --- | --- | --- | --- | --- | --- | --- | --- |
| Effect | | Value | F | Hypothesis df | Error df | Sig. | Partial Eta Squared | Noncent. Parameter | Observed Power^b^ |
| groups | Pillai's Trace | .257 | 4.852^a^ | 1.000 | 14.000 | .045 | .257 | 4.852 | .536 |
|  | Wilks' Lambda | .743 | 4.852^a^ | 1.000 | 14.000 | .045 | .257 | 4.852 | .536 |
|  | Hotelling's Trace | .347 | 4.852^a^ | 1.000 | 14.000 | .045 | .257 | 4.852 | .536 |
|  | Roy's Largest Root | .347 | 4.852^a^ | 1.000 | 14.000 | .045 | .257 | 4.852 | .536 |
| time | Pillai's Trace | .001 | .014^a^ | 1.000 | 14.000 | .909 | .001 | .014 | .051 |
|  | Wilks' Lambda | .999 | .014^a^ | 1.000 | 14.000 | .909 | .001 | .014 | .051 |
|  | Hotelling's Trace | .001 | .014^a^ | 1.000 | 14.000 | .909 | .001 | .014 | .051 |
|  | Roy's Largest Root | .001 | .014^a^ | 1.000 | 14.000 | .909 | .001 | .014 | .051 |
| groups * time | Pillai's Trace | .018 | .253^a^ | 1.000 | 14.000 | .623 | .018 | .253 | .076 |
|  | Wilks' Lambda | .982 | .253^a^ | 1.000 | 14.000 | .623 | .018 | .253 | .076 |
|  | Hotelling's Trace | .018 | .253^a^ | 1.000 | 14.000 | .623 | .018 | .253 | .076 |
|  | Roy's Largest Root | .018 | .253^a^ | 1.000 | 14.000 | .623 | .018 | .253 | .076 |
| a. Exact statistic | |  |  |  |  |  |  |  |  |
| b. Computed using alpha = .05 | | |  |  |  |  |  |  |  |
| c. Design: Intercept  Within Subjects Design: groups + time + groups * time | | | |  |  |  |  |  |  |

| **Tests of Within-Subjects Effects** | | | | | | | | | |
| --- | --- | --- | --- | --- | --- | --- | --- | --- | --- |
| Measure:MEASURE_1 | |  |  |  |  |  |  |  |  |
| Source | | Type III Sum of Squares | df | Mean Square | F | Sig. | Partial Eta Squared | Noncent. Parameter | Observed Power^a^ |
| groups | Sphericity Assumed | 196.757 | 1 | 196.757 | 4.852 | .045 | .257 | 4.852 | .536 |
|  | Greenhouse-Geisser | 196.757 | 1.000 | 196.757 | 4.852 | .045 | .257 | 4.852 | .536 |
|  | Huynh-Feldt | 196.757 | 1.000 | 196.757 | 4.852 | .045 | .257 | 4.852 | .536 |
|  | Lower-bound | 196.757 | 1.000 | 196.757 | 4.852 | .045 | .257 | 4.852 | .536 |
| Error(groups) | Sphericity Assumed | 567.725 | 14 | 40.552 |  |  |  |  |  |
|  | Greenhouse-Geisser | 567.725 | 14.000 | 40.552 |  |  |  |  |  |
|  | Huynh-Feldt | 567.725 | 14.000 | 40.552 |  |  |  |  |  |
|  | Lower-bound | 567.725 | 14.000 | 40.552 |  |  |  |  |  |
| time | Sphericity Assumed | .426 | 1 | .426 | .014 | .909 | .001 | .014 | .051 |
|  | Greenhouse-Geisser | .426 | 1.000 | .426 | .014 | .909 | .001 | .014 | .051 |
|  | Huynh-Feldt | .426 | 1.000 | .426 | .014 | .909 | .001 | .014 | .051 |
|  | Lower-bound | .426 | 1.000 | .426 | .014 | .909 | .001 | .014 | .051 |
| Error(time) | Sphericity Assumed | 438.783 | 14 | 31.342 |  |  |  |  |  |
|  | Greenhouse-Geisser | 438.783 | 14.000 | 31.342 |  |  |  |  |  |
|  | Huynh-Feldt | 438.783 | 14.000 | 31.342 |  |  |  |  |  |
|  | Lower-bound | 438.783 | 14.000 | 31.342 |  |  |  |  |  |
| groups * time | Sphericity Assumed | 12.975 | 1 | 12.975 | .253 | .623 | .018 | .253 | .076 |
|  | Greenhouse-Geisser | 12.975 | 1.000 | 12.975 | .253 | .623 | .018 | .253 | .076 |
|  | Huynh-Feldt | 12.975 | 1.000 | 12.975 | .253 | .623 | .018 | .253 | .076 |
|  | Lower-bound | 12.975 | 1.000 | 12.975 | .253 | .623 | .018 | .253 | .076 |
| Error(groups*time) | Sphericity Assumed | 718.628 | 14 | 51.331 |  |  |  |  |  |
|  | Greenhouse-Geisser | 718.628 | 14.000 | 51.331 |  |  |  |  |  |
|  | Huynh-Feldt | 718.628 | 14.000 | 51.331 |  |  |  |  |  |
|  | Lower-bound | 718.628 | 14.000 | 51.331 |  |  |  |  |  |
| a. Computed using alpha = .05 | |  |  |  |  |  |  |  |  |

Results of Two Way ANOVA for Fy_HC_

| **Descriptive Statistics** | | | |
| --- | --- | --- | --- |
|  | Mean | Std. Deviation | N |
| FYhcPr | -30.9418 | 9.94399 | 15 |
| FYhcPo | -22.8943 | 5.20837 | 15 |
| FYhcPrCG | -28.5480 | 6.83340 | 15 |
| FYhcPoCG | -29.7828 | 7.73682 | 15 |

| **Multivariate Tests^c^** | | | | | | | | | |
| --- | --- | --- | --- | --- | --- | --- | --- | --- | --- |
| Effect | | Value | F | Hypothesis df | Error df | Sig. | Partial Eta Squared | Noncent. Parameter | Observed Power^b^ |
| groups | Pillai's Trace | .087 | 1.337^a^ | 1.000 | 14.000 | .267 | .087 | 1.337 | .190 |
|  | Wilks' Lambda | .913 | 1.337^a^ | 1.000 | 14.000 | .267 | .087 | 1.337 | .190 |
|  | Hotelling's Trace | .095 | 1.337^a^ | 1.000 | 14.000 | .267 | .087 | 1.337 | .190 |
|  | Roy's Largest Root | .095 | 1.337^a^ | 1.000 | 14.000 | .267 | .087 | 1.337 | .190 |
| time | Pillai's Trace | .197 | 3.428^a^ | 1.000 | 14.000 | .085 | .197 | 3.428 | .407 |
|  | Wilks' Lambda | .803 | 3.428^a^ | 1.000 | 14.000 | .085 | .197 | 3.428 | .407 |
|  | Hotelling's Trace | .245 | 3.428^a^ | 1.000 | 14.000 | .085 | .197 | 3.428 | .407 |
|  | Roy's Largest Root | .245 | 3.428^a^ | 1.000 | 14.000 | .085 | .197 | 3.428 | .407 |
| groups * time | Pillai's Trace | .372 | 8.277^a^ | 1.000 | 14.000 | .012 | .372 | 8.277 | .763 |
|  | Wilks' Lambda | .628 | 8.277^a^ | 1.000 | 14.000 | .012 | .372 | 8.277 | .763 |
|  | Hotelling's Trace | .591 | 8.277^a^ | 1.000 | 14.000 | .012 | .372 | 8.277 | .763 |
|  | Roy's Largest Root | .591 | 8.277^a^ | 1.000 | 14.000 | .012 | .372 | 8.277 | .763 |
| a. Exact statistic | |  |  |  |  |  |  |  |  |
| b. Computed using alpha = .05 | | |  |  |  |  |  |  |  |
| c. Design: Intercept  Within Subjects Design: groups + time + groups * time | | | |  |  |  |  |  |  |

| **Tests of Within-Subjects Effects** | | | | | | | | | |
| --- | --- | --- | --- | --- | --- | --- | --- | --- | --- |
| Measure:MEASURE_1 | |  |  |  |  |  |  |  |  |
| Source | | Type III Sum of Squares | df | Mean Square | F | Sig. | Partial Eta Squared | Noncent. Parameter | Observed Power^a^ |
| groups | Sphericity Assumed | 75.755 | 1 | 75.755 | 1.337 | .267 | .087 | 1.337 | .190 |
|  | Greenhouse-Geisser | 75.755 | 1.000 | 75.755 | 1.337 | .267 | .087 | 1.337 | .190 |
|  | Huynh-Feldt | 75.755 | 1.000 | 75.755 | 1.337 | .267 | .087 | 1.337 | .190 |
|  | Lower-bound | 75.755 | 1.000 | 75.755 | 1.337 | .267 | .087 | 1.337 | .190 |
| Error(groups) | Sphericity Assumed | 793.509 | 14 | 56.679 |  |  |  |  |  |
|  | Greenhouse-Geisser | 793.509 | 14.000 | 56.679 |  |  |  |  |  |
|  | Huynh-Feldt | 793.509 | 14.000 | 56.679 |  |  |  |  |  |
|  | Lower-bound | 793.509 | 14.000 | 56.679 |  |  |  |  |  |
| time | Sphericity Assumed | 174.048 | 1 | 174.048 | 3.428 | .085 | .197 | 3.428 | .407 |
|  | Greenhouse-Geisser | 174.048 | 1.000 | 174.048 | 3.428 | .085 | .197 | 3.428 | .407 |
|  | Huynh-Feldt | 174.048 | 1.000 | 174.048 | 3.428 | .085 | .197 | 3.428 | .407 |
|  | Lower-bound | 174.048 | 1.000 | 174.048 | 3.428 | .085 | .197 | 3.428 | .407 |
| Error(time) | Sphericity Assumed | 710.786 | 14 | 50.770 |  |  |  |  |  |
|  | Greenhouse-Geisser | 710.786 | 14.000 | 50.770 |  |  |  |  |  |
|  | Huynh-Feldt | 710.786 | 14.000 | 50.770 |  |  |  |  |  |
|  | Lower-bound | 710.786 | 14.000 | 50.770 |  |  |  |  |  |
| groups * time | Sphericity Assumed | 323.107 | 1 | 323.107 | 8.277 | .012 | .372 | 8.277 | .763 |
|  | Greenhouse-Geisser | 323.107 | 1.000 | 323.107 | 8.277 | .012 | .372 | 8.277 | .763 |
|  | Huynh-Feldt | 323.107 | 1.000 | 323.107 | 8.277 | .012 | .372 | 8.277 | .763 |
|  | Lower-bound | 323.107 | 1.000 | 323.107 | 8.277 | .012 | .372 | 8.277 | .763 |
| Error(groups*time) | Sphericity Assumed | 546.487 | 14 | 39.035 |  |  |  |  |  |
|  | Greenhouse-Geisser | 546.487 | 14.000 | 39.035 |  |  |  |  |  |
|  | Huynh-Feldt | 546.487 | 14.000 | 39.035 |  |  |  |  |  |
|  | Lower-bound | 546.487 | 14.000 | 39.035 |  |  |  |  |  |
| a. Computed using alpha = .05 | |  |  |  |  |  |  |  |  |

Results of Two Way ANOVA for Fy_PO_

| **Descriptive Statistics** | | | |
| --- | --- | --- | --- |
|  | Mean | Std. Deviation | N |
| FYpoPr | 33.6148 | 8.83350 | 15 |
| FYpoPo | 34.3761 | 9.65212 | 15 |
| FYpoPrCG | 35.5731 | 5.60292 | 15 |
| FYpoPoCG | 36.0082 | 8.67515 | 15 |

| **Multivariate Tests^c^** | | | | | | | | | |
| --- | --- | --- | --- | --- | --- | --- | --- | --- | --- |
| Effect | | Value | F | Hypothesis df | Error df | Sig. | Partial Eta Squared | Noncent. Parameter | Observed Power^b^ |
| groups | Pillai's Trace | .082 | 1.243^a^ | 1.000 | 14.000 | .284 | .082 | 1.243 | .180 |
|  | Wilks' Lambda | .918 | 1.243^a^ | 1.000 | 14.000 | .284 | .082 | 1.243 | .180 |
|  | Hotelling's Trace | .089 | 1.243^a^ | 1.000 | 14.000 | .284 | .082 | 1.243 | .180 |
|  | Roy's Largest Root | .089 | 1.243^a^ | 1.000 | 14.000 | .284 | .082 | 1.243 | .180 |
| time | Pillai's Trace | .005 | .076^a^ | 1.000 | 14.000 | .786 | .005 | .076 | .058 |
|  | Wilks' Lambda | .995 | .076^a^ | 1.000 | 14.000 | .786 | .005 | .076 | .058 |
|  | Hotelling's Trace | .005 | .076^a^ | 1.000 | 14.000 | .786 | .005 | .076 | .058 |
|  | Roy's Largest Root | .005 | .076^a^ | 1.000 | 14.000 | .786 | .005 | .076 | .058 |
| groups * time | Pillai's Trace | .000 | .006^a^ | 1.000 | 14.000 | .940 | .000 | .006 | .051 |
|  | Wilks' Lambda | 1.000 | .006^a^ | 1.000 | 14.000 | .940 | .000 | .006 | .051 |
|  | Hotelling's Trace | .000 | .006^a^ | 1.000 | 14.000 | .940 | .000 | .006 | .051 |
|  | Roy's Largest Root | .000 | .006^a^ | 1.000 | 14.000 | .940 | .000 | .006 | .051 |
| a. Exact statistic | |  |  |  |  |  |  |  |  |
| b. Computed using alpha = .05 | | |  |  |  |  |  |  |  |
| c. Design: Intercept  Within Subjects Design: groups + time + groups * time | | | |  |  |  |  |  |  |

| **Tests of Within-Subjects Effects** | | | | | | | | | |
| --- | --- | --- | --- | --- | --- | --- | --- | --- | --- |
| Measure:MEASURE_1 | |  |  |  |  |  |  |  |  |
| Source | | Type III Sum of Squares | df | Mean Square | F | Sig. | Partial Eta Squared | Noncent. Parameter | Observed Power^a^ |
| groups | Sphericity Assumed | 48.343 | 1 | 48.343 | 1.243 | .284 | .082 | 1.243 | .180 |
|  | Greenhouse-Geisser | 48.343 | 1.000 | 48.343 | 1.243 | .284 | .082 | 1.243 | .180 |
|  | Huynh-Feldt | 48.343 | 1.000 | 48.343 | 1.243 | .284 | .082 | 1.243 | .180 |
|  | Lower-bound | 48.343 | 1.000 | 48.343 | 1.243 | .284 | .082 | 1.243 | .180 |
| Error(groups) | Sphericity Assumed | 544.387 | 14 | 38.885 |  |  |  |  |  |
|  | Greenhouse-Geisser | 544.387 | 14.000 | 38.885 |  |  |  |  |  |
|  | Huynh-Feldt | 544.387 | 14.000 | 38.885 |  |  |  |  |  |
|  | Lower-bound | 544.387 | 14.000 | 38.885 |  |  |  |  |  |
| time | Sphericity Assumed | 5.367 | 1 | 5.367 | .076 | .786 | .005 | .076 | .058 |
|  | Greenhouse-Geisser | 5.367 | 1.000 | 5.367 | .076 | .786 | .005 | .076 | .058 |
|  | Huynh-Feldt | 5.367 | 1.000 | 5.367 | .076 | .786 | .005 | .076 | .058 |
|  | Lower-bound | 5.367 | 1.000 | 5.367 | .076 | .786 | .005 | .076 | .058 |
| Error(time) | Sphericity Assumed | 984.753 | 14 | 70.339 |  |  |  |  |  |
|  | Greenhouse-Geisser | 984.753 | 14.000 | 70.339 |  |  |  |  |  |
|  | Huynh-Feldt | 984.753 | 14.000 | 70.339 |  |  |  |  |  |
|  | Lower-bound | 984.753 | 14.000 | 70.339 |  |  |  |  |  |
| groups * time | Sphericity Assumed | .399 | 1 | .399 | .006 | .940 | .000 | .006 | .051 |
|  | Greenhouse-Geisser | .399 | 1.000 | .399 | .006 | .940 | .000 | .006 | .051 |
|  | Huynh-Feldt | .399 | 1.000 | .399 | .006 | .940 | .000 | .006 | .051 |
|  | Lower-bound | .399 | 1.000 | .399 | .006 | .940 | .000 | .006 | .051 |
| Error(groups*time) | Sphericity Assumed | 959.930 | 14 | 68.566 |  |  |  |  |  |
|  | Greenhouse-Geisser | 959.930 | 14.000 | 68.566 |  |  |  |  |  |
|  | Huynh-Feldt | 959.930 | 14.000 | 68.566 |  |  |  |  |  |
|  | Lower-bound | 959.930 | 14.000 | 68.566 |  |  |  |  |  |
| a. Computed using alpha = .05 | |  |  |  |  |  |  |  |  |

Results of Two Way ANOVA for Fx_HC_

| **Descriptive Statistics** | | | |
| --- | --- | --- | --- |
|  | Mean | Std. Deviation | N |
| FXhcPr | 7.6420 | 4.26927 | 15 |
| FXhcPo | 5.6925 | 4.24883 | 15 |
| FXhcPrCG | 10.2115 | 6.06331 | 15 |
| FXhcPoCG | 9.6768 | 5.85311 | 15 |

| **Multivariate Tests^c^** | | | | | | | | | |
| --- | --- | --- | --- | --- | --- | --- | --- | --- | --- |
| Effect | | Value | F | Hypothesis df | Error df | Sig. | Partial Eta Squared | Noncent. Parameter | Observed Power^b^ |
| groups | Pillai's Trace | .270 | 5.191^a^ | 1.000 | 14.000 | .039 | .270 | 5.191 | .564 |
|  | Wilks' Lambda | .730 | 5.191^a^ | 1.000 | 14.000 | .039 | .270 | 5.191 | .564 |
|  | Hotelling's Trace | .371 | 5.191^a^ | 1.000 | 14.000 | .039 | .270 | 5.191 | .564 |
|  | Roy's Largest Root | .371 | 5.191^a^ | 1.000 | 14.000 | .039 | .270 | 5.191 | .564 |
| time | Pillai's Trace | .099 | 1.542^a^ | 1.000 | 14.000 | .235 | .099 | 1.542 | .212 |
|  | Wilks' Lambda | .901 | 1.542^a^ | 1.000 | 14.000 | .235 | .099 | 1.542 | .212 |
|  | Hotelling's Trace | .110 | 1.542^a^ | 1.000 | 14.000 | .235 | .099 | 1.542 | .212 |
|  | Roy's Largest Root | .110 | 1.542^a^ | 1.000 | 14.000 | .235 | .099 | 1.542 | .212 |
| groups * time | Pillai's Trace | .017 | .241^a^ | 1.000 | 14.000 | .631 | .017 | .241 | .074 |
|  | Wilks' Lambda | .983 | .241^a^ | 1.000 | 14.000 | .631 | .017 | .241 | .074 |
|  | Hotelling's Trace | .017 | .241^a^ | 1.000 | 14.000 | .631 | .017 | .241 | .074 |
|  | Roy's Largest Root | .017 | .241^a^ | 1.000 | 14.000 | .631 | .017 | .241 | .074 |
| a. Exact statistic | |  |  |  |  |  |  |  |  |
| b. Computed using alpha = .05 | | |  |  |  |  |  |  |  |
| c. Design: Intercept  Within Subjects Design: groups + time + groups * time | | | |  |  |  |  |  |  |

| **Tests of Within-Subjects Effects** | | | | | | | | | |
| --- | --- | --- | --- | --- | --- | --- | --- | --- | --- |
| Measure:MEASURE_1 | |  |  |  |  |  |  |  |  |
| Source | | Type III Sum of Squares | df | Mean Square | F | Sig. | Partial Eta Squared | Noncent. Parameter | Observed Power^a^ |
| groups | Sphericity Assumed | 161.072 | 1 | 161.072 | 5.191 | .039 | .270 | 5.191 | .564 |
|  | Greenhouse-Geisser | 161.072 | 1.000 | 161.072 | 5.191 | .039 | .270 | 5.191 | .564 |
|  | Huynh-Feldt | 161.072 | 1.000 | 161.072 | 5.191 | .039 | .270 | 5.191 | .564 |
|  | Lower-bound | 161.072 | 1.000 | 161.072 | 5.191 | .039 | .270 | 5.191 | .564 |
| Error(groups) | Sphericity Assumed | 434.407 | 14 | 31.029 |  |  |  |  |  |
|  | Greenhouse-Geisser | 434.407 | 14.000 | 31.029 |  |  |  |  |  |
|  | Huynh-Feldt | 434.407 | 14.000 | 31.029 |  |  |  |  |  |
|  | Lower-bound | 434.407 | 14.000 | 31.029 |  |  |  |  |  |
| time | Sphericity Assumed | 23.142 | 1 | 23.142 | 1.542 | .235 | .099 | 1.542 | .212 |
|  | Greenhouse-Geisser | 23.142 | 1.000 | 23.142 | 1.542 | .235 | .099 | 1.542 | .212 |
|  | Huynh-Feldt | 23.142 | 1.000 | 23.142 | 1.542 | .235 | .099 | 1.542 | .212 |
|  | Lower-bound | 23.142 | 1.000 | 23.142 | 1.542 | .235 | .099 | 1.542 | .212 |
| Error(time) | Sphericity Assumed | 210.106 | 14 | 15.008 |  |  |  |  |  |
|  | Greenhouse-Geisser | 210.106 | 14.000 | 15.008 |  |  |  |  |  |
|  | Huynh-Feldt | 210.106 | 14.000 | 15.008 |  |  |  |  |  |
|  | Lower-bound | 210.106 | 14.000 | 15.008 |  |  |  |  |  |
| groups * time | Sphericity Assumed | 7.507 | 1 | 7.507 | .241 | .631 | .017 | .241 | .074 |
|  | Greenhouse-Geisser | 7.507 | 1.000 | 7.507 | .241 | .631 | .017 | .241 | .074 |
|  | Huynh-Feldt | 7.507 | 1.000 | 7.507 | .241 | .631 | .017 | .241 | .074 |
|  | Lower-bound | 7.507 | 1.000 | 7.507 | .241 | .631 | .017 | .241 | .074 |
| Error(groups*time) | Sphericity Assumed | 436.230 | 14 | 31.159 |  |  |  |  |  |
|  | Greenhouse-Geisser | 436.230 | 14.000 | 31.159 |  |  |  |  |  |
|  | Huynh-Feldt | 436.230 | 14.000 | 31.159 |  |  |  |  |  |
|  | Lower-bound | 436.230 | 14.000 | 31.159 |  |  |  |  |  |
| a. Computed using alpha = .05 | |  |  |  |  |  |  |  |  |

Results of Two Way ANOVA for Fx_MS_

| **Descriptive Statistics** | | | |
| --- | --- | --- | --- |
|  | Mean | Std. Deviation | N |
| FXmsPr | -11.0149 | 2.70499 | 15 |
| FXmsPo | -11.7512 | 2.67139 | 15 |
| FXmsPrCG | -11.1702 | 2.52110 | 15 |
| FXmsPoCG | -10.7113 | 3.04515 | 15 |

| **Multivariate Tests^c^** | | | | | | | | | |
| --- | --- | --- | --- | --- | --- | --- | --- | --- | --- |
| Effect | | Value | F | Hypothesis df | Error df | Sig. | Partial Eta Squared | Noncent. Parameter | Observed Power^b^ |
| groups | Pillai's Trace | .022 | .316^a^ | 1.000 | 14.000 | .583 | .022 | .316 | .082 |
|  | Wilks' Lambda | .978 | .316^a^ | 1.000 | 14.000 | .583 | .022 | .316 | .082 |
|  | Hotelling's Trace | .023 | .316^a^ | 1.000 | 14.000 | .583 | .022 | .316 | .082 |
|  | Roy's Largest Root | .023 | .316^a^ | 1.000 | 14.000 | .583 | .022 | .316 | .082 |
| time | Pillai's Trace | .004 | .052^a^ | 1.000 | 14.000 | .822 | .004 | .052 | .055 |
|  | Wilks' Lambda | .996 | .052^a^ | 1.000 | 14.000 | .822 | .004 | .052 | .055 |
|  | Hotelling's Trace | .004 | .052^a^ | 1.000 | 14.000 | .822 | .004 | .052 | .055 |
|  | Roy's Largest Root | .004 | .052^a^ | 1.000 | 14.000 | .822 | .004 | .052 | .055 |
| groups * time | Pillai's Trace | .044 | .643^a^ | 1.000 | 14.000 | .436 | .044 | .643 | .116 |
|  | Wilks' Lambda | .956 | .643^a^ | 1.000 | 14.000 | .436 | .044 | .643 | .116 |
|  | Hotelling's Trace | .046 | .643^a^ | 1.000 | 14.000 | .436 | .044 | .643 | .116 |
|  | Roy's Largest Root | .046 | .643^a^ | 1.000 | 14.000 | .436 | .044 | .643 | .116 |
| a. Exact statistic | |  |  |  |  |  |  |  |  |
| b. Computed using alpha = .05 | | |  |  |  |  |  |  |  |
| c. Design: Intercept  Within Subjects Design: groups + time + groups * time | | | |  |  |  |  |  |  |

| **Tests of Within-Subjects Effects** | | | | | | | | | |
| --- | --- | --- | --- | --- | --- | --- | --- | --- | --- |
| Measure:MEASURE_1 | |  |  |  |  |  |  |  |  |
| Source | | Type III Sum of Squares | df | Mean Square | F | Sig. | Partial Eta Squared | Noncent. Parameter | Observed Power^a^ |
| groups | Sphericity Assumed | 2.935 | 1 | 2.935 | .316 | .583 | .022 | .316 | .082 |
|  | Greenhouse-Geisser | 2.935 | 1.000 | 2.935 | .316 | .583 | .022 | .316 | .082 |
|  | Huynh-Feldt | 2.935 | 1.000 | 2.935 | .316 | .583 | .022 | .316 | .082 |
|  | Lower-bound | 2.935 | 1.000 | 2.935 | .316 | .583 | .022 | .316 | .082 |
| Error(groups) | Sphericity Assumed | 130.053 | 14 | 9.289 |  |  |  |  |  |
|  | Greenhouse-Geisser | 130.053 | 14.000 | 9.289 |  |  |  |  |  |
|  | Huynh-Feldt | 130.053 | 14.000 | 9.289 |  |  |  |  |  |
|  | Lower-bound | 130.053 | 14.000 | 9.289 |  |  |  |  |  |
| time | Sphericity Assumed | .289 | 1 | .289 | .052 | .822 | .004 | .052 | .055 |
|  | Greenhouse-Geisser | .289 | 1.000 | .289 | .052 | .822 | .004 | .052 | .055 |
|  | Huynh-Feldt | .289 | 1.000 | .289 | .052 | .822 | .004 | .052 | .055 |
|  | Lower-bound | .289 | 1.000 | .289 | .052 | .822 | .004 | .052 | .055 |
| Error(time) | Sphericity Assumed | 77.014 | 14 | 5.501 |  |  |  |  |  |
|  | Greenhouse-Geisser | 77.014 | 14.000 | 5.501 |  |  |  |  |  |
|  | Huynh-Feldt | 77.014 | 14.000 | 5.501 |  |  |  |  |  |
|  | Lower-bound | 77.014 | 14.000 | 5.501 |  |  |  |  |  |
| groups * time | Sphericity Assumed | 5.357 | 1 | 5.357 | .643 | .436 | .044 | .643 | .116 |
|  | Greenhouse-Geisser | 5.357 | 1.000 | 5.357 | .643 | .436 | .044 | .643 | .116 |
|  | Huynh-Feldt | 5.357 | 1.000 | 5.357 | .643 | .436 | .044 | .643 | .116 |
|  | Lower-bound | 5.357 | 1.000 | 5.357 | .643 | .436 | .044 | .643 | .116 |
| Error(groups*time) | Sphericity Assumed | 116.686 | 14 | 8.335 |  |  |  |  |  |
|  | Greenhouse-Geisser | 116.686 | 14.000 | 8.335 |  |  |  |  |  |
|  | Huynh-Feldt | 116.686 | 14.000 | 8.335 |  |  |  |  |  |
|  | Lower-bound | 116.686 | 14.000 | 8.335 |  |  |  |  |  |
| a. Computed using alpha = .05 | |  |  |  |  |  |  |  |  |

Results of Two Way ANOVA for Fx_PO_

| **Descriptive Statistics** | | | |
| --- | --- | --- | --- |
|  | Mean | Std. Deviation | N |
| FXpoPr | -11.2217 | 3.07993 | 15 |
| FXpoPo | -11.0379 | 1.89418 | 15 |
| FXpoPrCG | -11.6556 | 2.77900 | 15 |
| FXpoPoCG | -11.1775 | 3.06467 | 15 |

| **Multivariate Tests^c^** | | | | | | | | | |
| --- | --- | --- | --- | --- | --- | --- | --- | --- | --- |
| Effect | | Value | F | Hypothesis df | Error df | Sig. | Partial Eta Squared | Noncent. Parameter | Observed Power^b^ |
| groups | Pillai's Trace | .015 | .212^a^ | 1.000 | 14.000 | .652 | .015 | .212 | .071 |
|  | Wilks' Lambda | .985 | .212^a^ | 1.000 | 14.000 | .652 | .015 | .212 | .071 |
|  | Hotelling's Trace | .015 | .212^a^ | 1.000 | 14.000 | .652 | .015 | .212 | .071 |
|  | Roy's Largest Root | .015 | .212^a^ | 1.000 | 14.000 | .652 | .015 | .212 | .071 |
| time | Pillai's Trace | .017 | .249^a^ | 1.000 | 14.000 | .626 | .017 | .249 | .075 |
|  | Wilks' Lambda | .983 | .249^a^ | 1.000 | 14.000 | .626 | .017 | .249 | .075 |
|  | Hotelling's Trace | .018 | .249^a^ | 1.000 | 14.000 | .626 | .017 | .249 | .075 |
|  | Roy's Largest Root | .018 | .249^a^ | 1.000 | 14.000 | .626 | .017 | .249 | .075 |
| groups * time | Pillai's Trace | .002 | .032^a^ | 1.000 | 14.000 | .861 | .002 | .032 | .053 |
|  | Wilks' Lambda | .998 | .032^a^ | 1.000 | 14.000 | .861 | .002 | .032 | .053 |
|  | Hotelling's Trace | .002 | .032^a^ | 1.000 | 14.000 | .861 | .002 | .032 | .053 |
|  | Roy's Largest Root | .002 | .032^a^ | 1.000 | 14.000 | .861 | .002 | .032 | .053 |
| a. Exact statistic | |  |  |  |  |  |  |  |  |
| b. Computed using alpha = .05 | | |  |  |  |  |  |  |  |
| c. Design: Intercept  Within Subjects Design: groups + time + groups * time | | | |  |  |  |  |  |  |

| **Tests of Within-Subjects Effects** | | | | | | | | | |
| --- | --- | --- | --- | --- | --- | --- | --- | --- | --- |
| Measure:MEASURE_1 | |  |  |  |  |  |  |  |  |
| Source | | Type III Sum of Squares | df | Mean Square | F | Sig. | Partial Eta Squared | Noncent. Parameter | Observed Power^a^ |
| groups | Sphericity Assumed | 1.234 | 1 | 1.234 | .212 | .652 | .015 | .212 | .071 |
|  | Greenhouse-Geisser | 1.234 | 1.000 | 1.234 | .212 | .652 | .015 | .212 | .071 |
|  | Huynh-Feldt | 1.234 | 1.000 | 1.234 | .212 | .652 | .015 | .212 | .071 |
|  | Lower-bound | 1.234 | 1.000 | 1.234 | .212 | .652 | .015 | .212 | .071 |
| Error(groups) | Sphericity Assumed | 81.455 | 14 | 5.818 |  |  |  |  |  |
|  | Greenhouse-Geisser | 81.455 | 14.000 | 5.818 |  |  |  |  |  |
|  | Huynh-Feldt | 81.455 | 14.000 | 5.818 |  |  |  |  |  |
|  | Lower-bound | 81.455 | 14.000 | 5.818 |  |  |  |  |  |
| time | Sphericity Assumed | 1.643 | 1 | 1.643 | .249 | .626 | .017 | .249 | .075 |
|  | Greenhouse-Geisser | 1.643 | 1.000 | 1.643 | .249 | .626 | .017 | .249 | .075 |
|  | Huynh-Feldt | 1.643 | 1.000 | 1.643 | .249 | .626 | .017 | .249 | .075 |
|  | Lower-bound | 1.643 | 1.000 | 1.643 | .249 | .626 | .017 | .249 | .075 |
| Error(time) | Sphericity Assumed | 92.443 | 14 | 6.603 |  |  |  |  |  |
|  | Greenhouse-Geisser | 92.443 | 14.000 | 6.603 |  |  |  |  |  |
|  | Huynh-Feldt | 92.443 | 14.000 | 6.603 |  |  |  |  |  |
|  | Lower-bound | 92.443 | 14.000 | 6.603 |  |  |  |  |  |
| groups * time | Sphericity Assumed | .325 | 1 | .325 | .032 | .861 | .002 | .032 | .053 |
|  | Greenhouse-Geisser | .325 | 1.000 | .325 | .032 | .861 | .002 | .032 | .053 |
|  | Huynh-Feldt | .325 | 1.000 | .325 | .032 | .861 | .002 | .032 | .053 |
|  | Lower-bound | .325 | 1.000 | .325 | .032 | .861 | .002 | .032 | .053 |
| Error(groups*time) | Sphericity Assumed | 143.820 | 14 | 10.273 |  |  |  |  |  |
|  | Greenhouse-Geisser | 143.820 | 14.000 | 10.273 |  |  |  |  |  |
|  | Huynh-Feldt | 143.820 | 14.000 | 10.273 |  |  |  |  |  |
|  | Lower-bound | 143.820 | 14.000 | 10.273 |  |  |  |  |  |
| a. Computed using alpha = .05 | |  |  |  |  |  |  |  |  |
