# Supplementary material for: The long-term use of foot orthoses affects walking kinematics and kinetics of children with flexible flat feet: A randomized controlled trial
Source: PLoS One. 2018 Oct 9;13(10):e0205187. doi: 10.1371/journal.pone.0205187 (PMC6177172; doi:10.1371/journal.pone.0205187)
Supplement: S3 File — (DOCX) [file pone.0205187.s003.docx]

Letter of registration code in IRCT


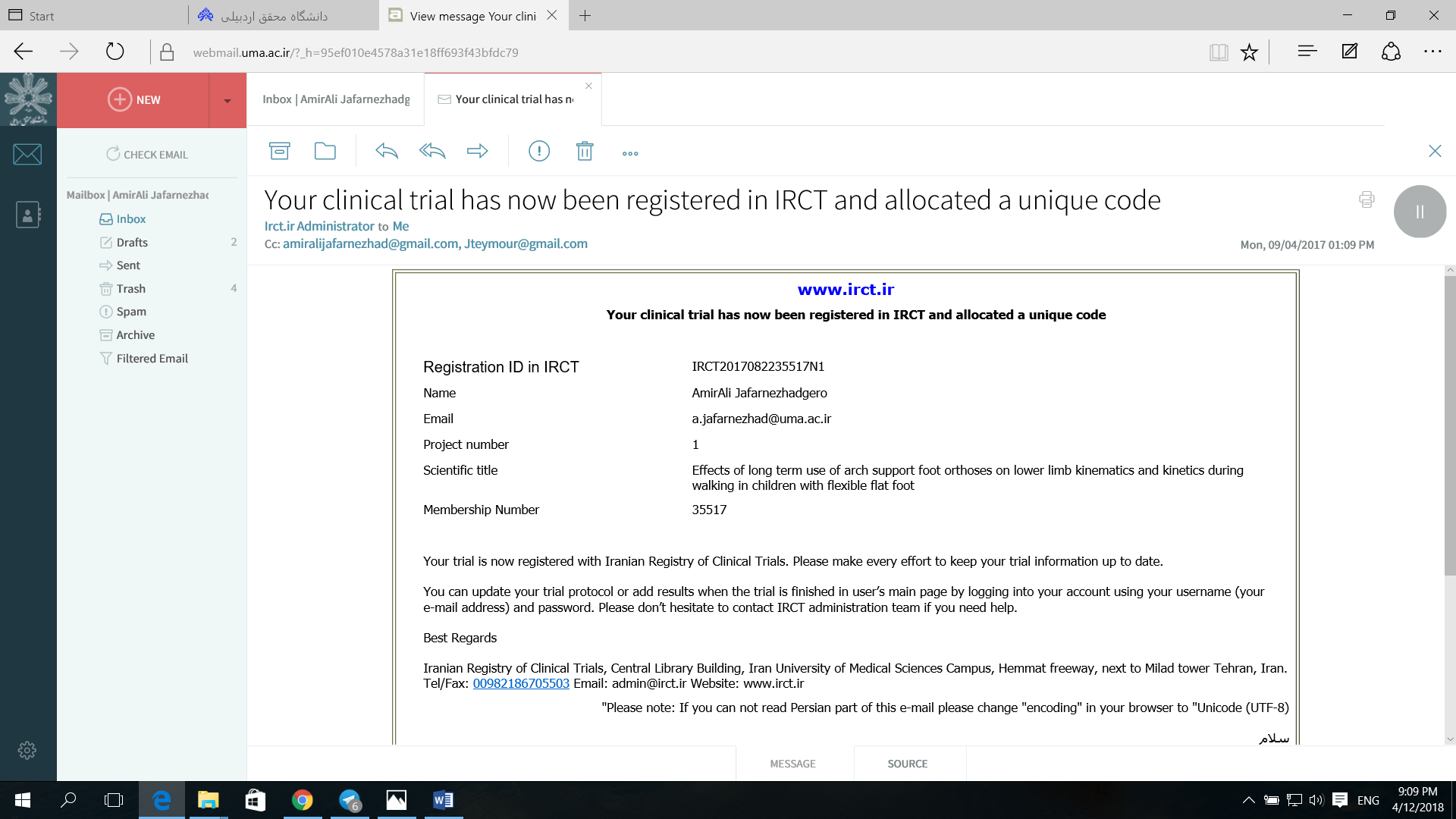


Ethics committee letter:

Date: 20/08/2017


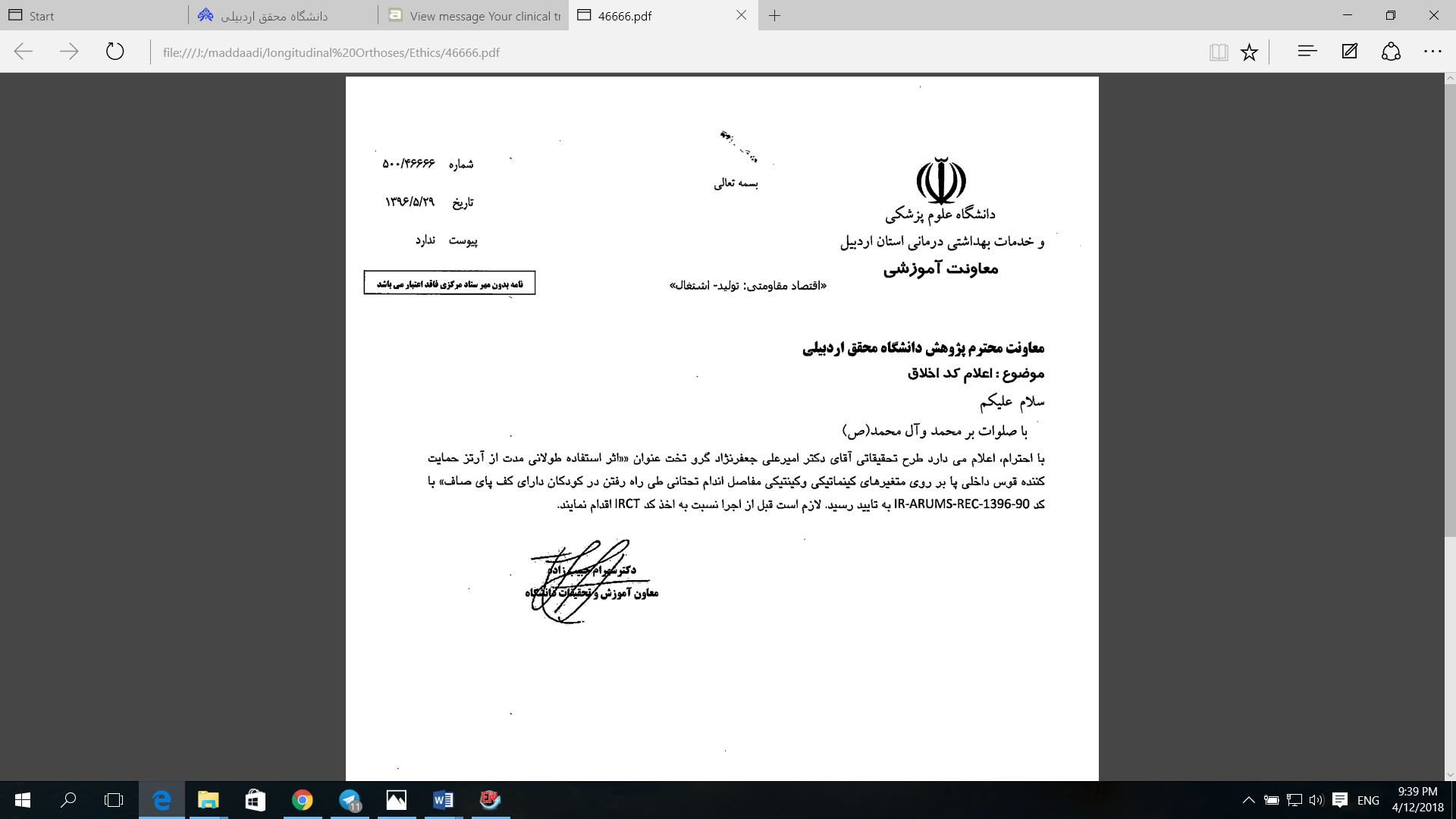
Ethics approval for the project "***Effects of long-term use of arch support foot orthoses on walking kinematics and kinetics in children with flexible flat feet: A randomized controlled trial***" was obtained with the following code (IR-ARUMS-REC-1396-90) from the Research Ethics Board of the Medical Sciences, University of Ardabil, Islamic Republic of Iran.

**Our study protocol in English**

**Effects of long-term use of arch support foot orthoses on lower limb kinematics and kinetics during walking in children with flexible flat feet: A randomized controlled trial**

1. **Background**

Based on previous studies, about 4 percent of children aged 10 suffered from flat feet (FF) and 10% of these individuals indicated flexible FF that needs treatment to prevent secondary deformity in their adulthood ([Ozonoff, 1992](#_ENREF_31); [Riskowski et al., 2013](#_ENREF_36)). It has been demonstrated that children with flexible FF are more likely to feel pain or discomfort at the knee, hip, and back ([Farahpour, Jafarnezhad, Damavandi, Bakhtiari, & Allard, 2016](#_ENREF_14); [Kothari, Dixon, Stebbins, Zavatsky, & Theologis, 2016](#_ENREF_24)). A variety of planned interventions are used to treat such problems, with foot orthoses (FOs) commonly utilized ([Christian J Barton, Menz, Levinger, Webster, & Crossley, 2011](#_ENREF_4); [Bowring & Chockalingam, 2010](#_ENREF_7); [AmirAli Jafarnezhadgero, Shad, & Ferber, 2018](#_ENREF_19); [A. A. Jafarnezhadgero, Shad, & Majlesi, 2017b](#_ENREF_22); [Williams, Hill, & Nester, 2013](#_ENREF_39)).

In a recent website audit, it was found that 47% of Canadian Chiropractor’s websites advertised selling orthotics ([Page & Grod, 2009a](#_ENREF_32)), and a mail survey of Alberta Canadian Chiropractors in 2011 indicated that 77% of respondents declared they sold orthotics ([Page, Grod, & McMorland, 2011a](#_ENREF_34)). Despite the fact that the effectiveness of using FOs was successfully demonstrated in previous studies ([Andreasen et al., 2013](#_ENREF_1); [N. J. Collins, Hinman, Menz, & Crossley, 2017](#_ENREF_10)), the biomechanical mechanisms underlying the therapeutic effect of FOs in children with FF are still unclear. Furthermore, many of the previous researches have examined the immediate or acute effects of using FOs on walking biomechanics. For example, Jafarnezhadgero et al. (2017) reported acute effects of FO use and reported altered three dimensional moments of lower limb joints as well as decreases limb asymmetries during walking ([AmirAli Jafarnezhadgero et al., 2018](#_ENREF_19); [A. A. Jafarnezhadgero, M. M. Shad, et al., 2017b](#_ENREF_22)). Moreover, previous research examined the acute effects of medially posted FOs on walking and running mechanics. Small reductions in the peak hind foot eversion (up to 1-3 degrees) were noted ([Eslami et al., 2009](#_ENREF_13); [Liu et al., 2012](#_ENREF_26); [Tang et al., 2015](#_ENREF_37)). Some studies have also shown alterations in frontal plane moments, particularly at the ankle and knee joints, when walking or running with medially posted FOs ([Nester, Van Der Linden, & Bowker, 2003](#_ENREF_30); [Telfer, Abbott, Steultjens, Rafferty, & Woodburn, 2013](#_ENREF_38)). Numerous studies have quantified the acute effects of FOs on lower limb kinematics and GRF characteristics during walking with controversial results. For example, while prior studies observed decreased peak forefoot pronation ([Hsu et al., 2014](#_ENREF_16)), others reported alterations primarily at the rear-foot rather than fore-foot ([Bishop, Arnold, & May, 2016](#_ENREF_5); [Ferber & Benson, 2011](#_ENREF_15)). While considerable knowledge has accumulated on the acute effects of foot orthoses on lower limb mechanics, little is known in regards of the chronic impact of FO application which has the potential to induce long-term adaptations. Our literature analysis revealed one study only that addressed the long-term use of FOs with lateral bar on lower limb muscle activation before and after a one-month period of therapy ([Moisan & Cantin, 2016](#_ENREF_29)). These authors reported that FOs with lateral bar decreased peak amplitude and mean activity of the peroneus longus muscle during the combined midstance/terminal stance phase ([Moisan & Cantin, 2016](#_ENREF_29)).

However, no study has quantified the effects of long-term use of arch support FOs on walking kinematics and kinetics in children with flexible FF. Therefore, the principal objective of this study was to evaluate the effects of four months arch support FO therapy on lower extremity kinematics and kinetics during walking in boys.

1. Hypothesis

- The long-term use of arch support foot orthoses can alter kinematics of lower limb segments in children with FF during walking.
- The long-term use of arch support foot orthoses can alter the kinetics of lower limb joints in children with FF during walking.
- The long-term use of arch support foot orthoses can alter GRF variables of walking in children with FF.

1. Purpose of study

The principal objective of this study was to evaluate the effects of four months arch support FO therapy on lower extremity kinematics and kinetics during walking in boys.

1. Necessity to do the examinations

- The high prevalence of FF in children.
- The incidence of secondary complications following FF.
- The paucity of information regarding different types of foot orthoses.
- Controversial results in the literature.

1. **Materials and methods**

Study design

The study was designed as a double-blind (i.e., participants, examiners) randomized controlled trial.

Participants

This study is semi-experimental. The date of recruitment will be scheduled from 20/08/2017 (after the date of receiving ethical committee code) up to 04/09/2017. Based on an a priori power analysis, at least 30 boys with flexible FF will be selected to participate in this study from September 2017 (after the date of receiving clinical trial registration code) to January 2018. The participants will be recruited from orthopaedic specialists in the local community, Hamadan city, Hamadan, Iran. Written informed consent will be obtained from the parents or legal representatives of all participants. Ethics approval will be obtained from the Research Ethics Board of the Medical Sciences, University of Ardabil, and will be registered with the Iranian Registry of Clinical Trials. To determine whether participants suffered from flexible FF, navicular drop, arch height index (AHI), and resting calcaneal stance position will be tested ([Bok, Kim, Lim, & Ahn, 2014](#_ENREF_6); [Cote, Brunet II, Gansneder, & Shultz, 2005](#_ENREF_11); [Jung et al., 2009](#_ENREF_23)). The measure of AHI is unitless and will be defined as the ratio of dorsal height at 50% of total foot length, divided by the foot length from the back of the heel to the head of the first metatarsal, defined as the truncated foot length ([AmirAli Jafarnezhadgero, Shad, & Ferber, 2017](#_ENREF_18))^2^. Standing AHI will be obtained with the participant standing with equally distributed weight on both feet. The flexible FF samples will contain participants whose feet will have a >10 mm navicular drop ([Cote, Brunet II, et al., 2005](#_ENREF_11)), >4° eversion in calcaneal stance position ([Bok et al., 2014](#_ENREF_6)), and arch height index less than 0.31 ([Butler, Hillstrom, Song, Richards, & Davis, 2008](#_ENREF_8)). A subject will be excluded from the study if he does not have any history of bone fractures, surgery, symptomatic FF, orthopedic diseases, neuromuscular problems, limb length asymmetries greater than 5 mm, or if he feels fatigued, or experienced strenuous exercising during the past two days. All participants will be right foot dominant which will be determined by means of a kicking ball test ([AA Jafarnezhadgero, Shad, Majlesi, & Granacher, 2017](#_ENREF_17)). Participants' age range will be between 9-12 years. Thereafter, participants (at least 30 subjects) will be randomly allocated to an experimental (EG) or a control group (CG). The EG will receive an insole with corrective elements (i.e., arch support FOs) and will use it for four months during everyday activities. The CG will be equipped with an insole without corrective elements (i.e., sham condition) and applied it for the same period of time.

During the randomization process, a set of sealed, opaque envelopes will be used to ensure the concealment of allocation. Each envelop will be contained a card indicating to which group the subject was allocated. Neither the participating child nor the parents will be aware of the respective group they will be allocated to. A naïve person that will not have a role in this study will be responsible for the allocation process.

The application of foot orthoses (FOs) represents an established therapeutic means that has also been recommended as part of the ‘Best Practice Guide’ for management of lower limb injuries ([Christian John Barton, Lack, Hemmings, Tufail, & Morrissey, 2015](#_ENREF_2); [Witvrouw et al., 2014](#_ENREF_40)). Clinical trials demonstrated that FOs are safe to use and superior to wait-and-see approaches ([Mills, Blanch, Dev, Martin, & Vicenzino, 2011](#_ENREF_28)) and flat insoles ([N. Collins et al., 2008](#_ENREF_9)). Due to the established efficacy of foot orthoses, we will use FOs treatment in this study.

Apparatus

Kinematics data will be collected at a sampling rate of 100 Hz using a six-camera VICON motion capture system (Vicon system, Oxford Metrics, Oxford, UK) and 16 spherical reflective markers with a diameter of 15 mm. The size of the cubic calibration volume will be 4.0 m (length) × 2.0 m (width) × 2.0 m (height) that will be located at the middle of a 15 m walkway. The Plug in Gait marker set will be used to identify the bilateral pelvis, thighs, legs, and feet provided in Nexus software. Two force platforms (Kistler, type 9281, Kistler Instrument AG, Winterthur, Switzerland) will be used to record GRF data from each leg at a sampling rate of 1000 Hz. Kinematic and kinetic data will synchronize using the Vicon system.

Application of orthoses

It has previously been demonstrated that shoe type affects the walking pattern in children ([Lythgo, Wilson, & Galea, 2009](#_ENREF_27); [Wolf et al., 2008](#_ENREF_41)). Therefore, all study participants will be equipped with the same neutral shoe model ([New Balance 759](http://www.newbalance.com/pd/new-balance-759/MW759.html?dwvar_MW759_color=Brown_with_Black_and_Orange), USA) with no intrinsic correction. Custom-made medial arch support FOs made out of ethylene vinyl acetate and microcellular rubber will be used to produce the negative impression of the foot held in subtalar joint neutral position in EG. In the EG, FOs peak longitudinal height of the mid-foot arch will be 25 mm. For the CG, flat 2-mm-thick insoles will be used as sham condition which will be made of polyester resin.

Test protocol

During baseline tests, data will be collected while walking with standard shoes and without FOs. After the pre-tests, each study participant will wear FOs (EG) and insoles (CG) for 4 months during everyday activities. During the intervention period, participants from both groups will be instructed to progressively increase the application time of their FOs (EG) and insoles (CG). On the first day, application time will be restricted to one hour to allow familiarization. On every following day, application time will be increased by one hour until participants finally wore the FOs (EG) / insoles (CG) for the full day ([Moisan & Cantin, 2016](#_ENREF_29)). On every day during the 4 months intervention period, participants will complete a log in which they documented the application time of FOs / insoles. Of note, post-tests will be conducted at the same time of day and in the similar test sequence as the baseline tests.

**References**

Andreasen, J., Mølgaard, C. M., Christensen, M., Kaalund, S., Lundbye-Christensen, S., Simonsen, O., & Voigt, M. (2013). Exercise therapy and custom-made insoles are effective in patients with excessive pronation and chronic foot pain—A randomized controlled trial. *The Foot, 23*(1), 22-28.

Barton, C. J., Lack, S., Hemmings, S., Tufail, S., & Morrissey, D. (2015). The ‘Best Practice Guide to Conservative Management of Patellofemoral Pain’: incorporating level 1 evidence with expert clinical reasoning. *Br J Sports Med, 49*(14), 923-934.

Barton, C. J., Menz, H. B., Levinger, P., Webster, K. E., & Crossley, K. M. (2010). Greater peak rearfoot eversion predicts foot orthoses efficacy in individuals with patellofemoral pain syndrome. *British journal of sports medicine*, bjsports77644.

Barton, C. J., Menz, H. B., Levinger, P., Webster, K. E., & Crossley, K. M. (2011). Greater peak rearfoot eversion predicts foot orthoses efficacy in individuals with patellofemoral pain syndrome. *British journal of sports medicine, 45*(9), 697-701.

Bishop, C., Arnold, J. B., & May, T. (2016). Effects of Taping and Orthoses on Foot Biomechanics in Adults with Flat-Arched Feet. *Medicine and Science in Sports and Exercise, 48*(4), 689-696.

Bok, S.-K., Kim, B.-O., Lim, J.-H., & Ahn, S.-Y. (2014). Effects of custom-made rigid foot orthosis on pes planus in children over 6 years old. *Annals of rehabilitation medicine, 38*(3), 369-375.

Bowring, B., & Chockalingam, N. (2010). Conservative treatment of tibialis posterior tendon dysfunction—A review. *The Foot, 20*(1), 18-26.

Butler, R. J., Hillstrom, H., Song, J., Richards, C. J., & Davis, I. S. (2008). Arch height index measurement system: establishment of reliability and normative values. *Journal of the American Podiatric Medical Association, 98*(2), 102-106.

Collins, N., Crossley, K., Beller, E., Darnell, R., McPoil, T., & Vicenzino, B. (2008). Foot orthoses and physiotherapy in the treatment of patellofemoral pain syndrome: randomised clinical trial. *Bmj, 337*, a1735.

Collins, N. J., Hinman, R. S., Menz, H. B., & Crossley, K. M. (2017). Immediate effects of foot orthoses on pain during functional tasks in people with patellofemoral osteoarthritis: a cross-over, proof-of-concept study. *The Knee, 24*(1), 76-81.

Cote, K. P., Brunet II, M. E., Gansneder, B. M., & Shultz, S. J. (2005). Effects of pronated and supinated foot postures on static and dynamic postural stability. *Journal of Athletic Training, 40*(1), 41.

Cote, K. P., Brunet, M. E., II, B. M. G., & Shultz, S. J. (2005). Effects of pronated and supinated foot postures on static and dynamic postural stability. *Journal of athletic training, 40*(1), 41.

Eslami, M., Begon, M., Hinse, S., Sadeghi, H., Popov, P., & Allard, P. (2009). Effect of foot orthoses on magnitude and timing of rearfoot and tibial motions, ground reaction force and knee moment during running. *Journal of Science and Medicine in Sport, 12*(6), 679-684.

Farahpour, N., Jafarnezhad, A., Damavandi, M., Bakhtiari, A., & Allard, P. (2016). Gait ground reaction force characteristics of low back pain patients with pronated foot and able-bodied individuals with and without foot pronation. *Journal of biomechanics, 49*(9), 1705-1710.

Ferber, R., & Benson, B. (2011). Changes in multi-segment foot biomechanics with a heat-mouldable semi-custom foot orthotic device. *Journal of Foot and Ankle research, 4*(1), 18.

Hsu, W.-H., Lewis, C. L., Monaghan, G. M., Saltzman, E., Hamill, J., & Holt, K. G. (2014). Orthoses posted in both the forefoot and rearfoot reduce moments and angular impulses on lower extremity joints during walking. *Journal of biomechanics, 47*(11), 2618-2625.

Jafarnezhadgero, A., Shad, M., Majlesi, M., & Granacher, U. (2017). A comparison of running kinetics in children with and without genu varus: A cross sectional study. *PloS one, 12*(9), e0185057.

Jafarnezhadgero, A., Shad, M. M., & Ferber, R. (2017). The effect of foot orthoses on joint moment asymmetry in male children with flexible flat feet. *Journal of Bodywork and Movement Therapies*.

Jafarnezhadgero, A., Shad, M. M., & Ferber, R. (2018). The effect of foot orthoses on joint moment asymmetry in male children with flexible flat feet. *Journal of bodywork and movement therapies, 22*(1), 83-89.

Jafarnezhadgero, A. A., Majlesi, M., & Azadian, E. (2017). Gait ground reaction force characteristics in deaf and hearing children. *Gait & posture, 53*, 236-240.

Jafarnezhadgero, A. A., Shad, M. M., & Majlesi, M. (2017a). Effect of foot orthoses on the medial longitudinal arch in children with flexible flatfoot deformity: A three-dimensional moment analysis. *Gait & posture*.

Jafarnezhadgero, A. A., Shad, M. M., & Majlesi, M. (2017b). Effect of foot orthoses on the medial longitudinal arch in children with flexible flatfoot deformity: A three-dimensional moment analysis. *Gait & posture, 55*, 75-80.

Jung, D.-Y., Koh, E.-K., Kwon, O.-Y., Yi, C.-H., Oh, J.-S., & Weon, J.-H. (2009). Effect of medial arch support on displacement of the myotendinous junction of the gastrocnemius during standing wall stretching. *journal of orthopaedic & sports physical therapy, 39*(12), 867-874.

Kothari, A., Dixon, P., Stebbins, J., Zavatsky, A., & Theologis, T. (2016). Are flexible flat feet associated with proximal joint problems in children? *Gait & posture, 45*, 204-210.

Lange, B., Chipchase, L., & Evans, A. (2004). The effect of low-Dye taping on plantar pressures, during gait, in subjects with navicular drop exceeding 10 mm. *Journal of Orthopaedic & Sports Physical Therapy, 34*(4), 201-209.

Liu, A., Nester, C. J., Jones, R. K., Lundgren, P., Lundberg, A., Arndt, A., & Wolf, P. (2012). Effect of an antipronation foot orthosis on ankle and subtalar kinematics. *Medicine and Science in Sports and Exercise, 44*(12), 2384-2391.

Lythgo, N., Wilson, C., & Galea, M. (2009). Basic gait and symmetry measures for primary school-aged children and young adults whilst walking barefoot and with shoes. *Gait & posture, 30*(4), 502-506.

Mills, K., Blanch, P., Dev, P., Martin, M., & Vicenzino, B. (2011). A randomised control trial of short term efficacy of in-shoe foot orthoses compared with a wait and see policy for anterior knee pain and the role of foot mobility. *Br J Sports Med*, bjsports-2011-090204.

Moisan, G., & Cantin, V. (2016). Effects of two types of foot orthoses on lower limb muscle activity before and after a one-month period of wear. *Gait & posture, 46*, 75-80.

Nester, C., Van Der Linden, M., & Bowker, P. (2003). Effect of foot orthoses on the kinematics and kinetics of normal walking gait. *Gait & posture, 17*(2), 180-187.

Ozonoff, M. B. (1992). Pediatric Orthopedic Radiology. *Journal of pediatric orthopaedics, 12*(4), 548.

Page, S. A., & Grod, J. P. (2009a). An audit of health products advertised for sale on chiropractic Web sites in Canada and consideration of these practices in the context of Canadian chiropractic codes of ethics and conduct. *Journal of Manipulative & Physiological Therapeutics, 32*(6), 485-492.

Page, S. A., & Grod, J. P. (2009b). An audit of health products advertised for sale on chiropractic Web sites in Canada and consideration of these practices in the context of Canadian chiropractic codes of ethics and conduct. *Journal of manipulative and physiological therapeutics, 32*(6), 485-492.

Page, S. A., Grod, J. P., & McMorland, D. G. (2011a). The perspectives and practices of Alberta chiropractors regarding the sale of health care products in chiropractic offices. *Journal of Manipulative & Physiological Therapeutics, 34*(7), 476-482.

Page, S. A., Grod, J. P., & McMorland, D. G. (2011b). The perspectives and practices of alberta chiropractors regarding the sale of health care products in chiropractic offices. *Journal of manipulative and physiological therapeutics, 34*(7), 476-482.

Riskowski, J. L., Dufour, A. B., Hagedorn, T. J., Hillstrom, H. J., Casey, V. A., & Hannan, M. T. (2013). Associations of Foot Posture and Function to Lower Extremity Pain: Results From a Population‐Based Foot Study. *Arthritis Care & Research, 65*(11), 1804-1812.

Tang, S. F.-T., Chen, C.-H., Wu, C.-K., Hong, W.-H., Chen, K.-J., & Chen, C.-K. (2015). The effects of total contact insole with forefoot medial posting on rearfoot movement and foot pressure distributions in patients with flexible flatfoot. *Clinical neurology and neurosurgery, 129*, S8-S11.

Telfer, S., Abbott, M., Steultjens, M., Rafferty, D., & Woodburn, J. (2013). Dose–response effects of customised foot orthoses on lower limb muscle activity and plantar pressures in pronated foot type. *Gait & posture, 38*(3), 443-449.

Williams, A. E., Hill, L. A., & Nester, C. J. (2013). Foot orthoses for the management of low back pain: a qualitative approach capturing the patient’s perspective. *Journal of Foot and Ankle research, 6*(1), 17.

Witvrouw, E., Callaghan, M. J., Stefanik, J. J., Noehren, B., Bazett-Jones, D. M., Willson, J. D., . . . McConnell, J. (2014). Patellofemoral pain: consensus statement from the 3rd International Patellofemoral Pain Research Retreat held in Vancouver, September 2013. *Br J Sports Med, 48*(6), 411-414.

Wolf, S., Simon, J., Patikas, D., Schuster, W., Armbrust, P., & Döderlein, L. (2008). Foot motion in children shoes—a comparison of barefoot walking with shod walking in conventional and flexible shoes. *Gait & posture, 27*(1), 51-59.

**Our study protocol in Persian**

**اثر استفاده طولانی­مدت از ارتز حمایت­کننده قوس طولی­داخلی پا بر روی متغیرهای کینماتیکی و کینتیکی مفاصل اندام تحتانی طی راه رفتن در کودکان دارای کف پای صاف**

الف – مقدمه

بر اساس مطالعات گذشته 4 درصد از کودکان در سن 10 سالگی از کف پای صاف رنج می­برند، که 10 درصد از این افراد دارای کف پای صاف منعطف هستند که می­تواند منجر به تغییرشکل و آسیب­های ثانویه در بزرگسالی گردد ([Ozonoff, 1992](#_ENREF_31); [Riskowski et al., 2013](#_ENREF_36)). گزارش شده است که کودکان مبتلا به کف پای صاف احتمال ابتلا به دردهای زانو، ران و کمر بیشتری را در مقایسه با کودکان سالم دارا هستند ([Farahpour et al., 2016](#_ENREF_14); [Kothari et al., 2016](#_ENREF_24)). مداخلات درمانی متفاوتی جهت پیشگیری از این آسیب­ها مورد استفاده قرار می­گیرد، رایجترین این مداخلات استفاده از ارتزهای پا می­باشد ([Christian J Barton, Menz, Levinger, Webster, & Crossley, 2010](#_ENREF_3); [Bowring & Chockalingam, 2010](#_ENREF_7); [AmirAli Jafarnezhadgero et al., 2017](#_ENREF_18); [A. A. Jafarnezhadgero, Shad, & Majlesi, 2017a](#_ENREF_21); [Williams et al., 2013](#_ENREF_39)).

در بررسی تبلیغات وب­سایت­ها مشخص شده است که 47 درصد از وبسایت­های تجهیزات پزشکی، فروش ارتزها را تبلیغ می­نمایند ([Page & Grod, 2009b](#_ENREF_33)). طی یک پژوهش گزارش شده است که 77 درصد از مشاهده­کنندگان این تبلیغات اقدام به خرید این ابزارهای ارتودیک می­نمایند ([Page, Grod, & McMorland, 2011b](#_ENREF_35)). علارغم وجود برخی شواهد بر کاهش درد ناشی از استفاده از ارتزهای پا ([Andreasen et al., 2013](#_ENREF_1); [N. J. Collins et al., 2017](#_ENREF_10))، مکانیزم­های بیومکانیکی این کاهش درد به لحاظ علمی و به ویژه در جامعه کودکان کمتر مورد بررسی قرار گرفته است ([AmirAli Jafarnezhadgero et al., 2017](#_ENREF_18); [A. A. Jafarnezhadgero, M. M. Shad, et al., 2017a](#_ENREF_21)). از سوی دیگر، اغلب مطالعات انجام شده با هدف بررسی اثرات ارتزها پا بر روی متغیرهای بیومکانیکی در کودکان با کف پای صاف به بررسی اثر آنی این تداخلات پرداخته­اند. به عنوان مثال جعفرنژادگرو و همکاران (2017) گزارش نمودند که استفاده آنی از کفی سبب کاهش شاخص عدم تقارن گشتاور آبداکتوری مفصل ران شده و همچنین گشتاور سه­بعدی مفاصل اندام تحتانی را تغییر می­دهد ([AmirAli Jafarnezhadgero et al., 2017](#_ENREF_18); [A. A. Jafarnezhadgero, M. M. Shad, et al., 2017a](#_ENREF_21)). همچنین مطالعات گذشته بر روی افراد بالغ نشان داده­اند که استفاده آنی از ارتزهای پا به طور معمول سبب کاهش اندک اورژن ریرفوت (بین 1 تا 3 درجه) می­گردد ([Eslami et al., 2009](#_ENREF_13); [Liu et al., 2012](#_ENREF_26); [Tang et al., 2015](#_ENREF_37)). برخی از مطالعات گزارش نموده­اند که استفاده آنی از ارتز پا سبب تغییر در گشتاور مربوط به صفحه فرونتال به ویژه در مفاصل مچ پا و زانو می­گردد ([Nester et al., 2003](#_ENREF_30); [Telfer et al., 2013](#_ENREF_38)). بسیاری از مطالعات گزارش نموده­اند که اثر آنی استفاده از ارتزهای پا نتایج متنقاضی را بر کینماتیک راه رفتن دارا می­باشند. به عنوان مثال؛ یک مطالعه کاهش پرونیش فورفوت را گزارش نموده­اند ([Hsu et al., 2014](#_ENREF_16))، در حالیکه مطالعات دیگر گزارش نموده­اند که استفاده آنی از ارتز پا عمدتا منجر به تغییر کینماتیک ریرفوت می­گردد ([Bishop et al., 2016](#_ENREF_5); [Ferber & Benson, 2011](#_ENREF_15)). یکی از نقایص مطالعات گذشته این است که اغلب به بررسی اثرات آنی استفاده از ارتزهای پا پرداخته­اند، حال آنکه استفاده طولانی­مدت از ارتزهای پا می­تواند منجر به سازگاری­های باثبات­تری گرد. پژوهشگر تنها یک مطالعه را مشاهده نمود که بررسی اثرات استفاده طولانی­مدت از ارتز پا پرداخته بود. در این مطالعه ([Moisan & Cantin, 2016](#_ENREF_29)) گزارش شد که دو ماه استفاده از کفی با گوه­ای در جانب خارجی در افراد سالم منجر به کاهش اوج و میانگین دامنه فعالیت عضله نازک­نئی طویل در طی میانه و انتهای فازاستقرار می­گردد. بعلاوه گزارش شد که استفاده طولانی مدت از ارتز به گوه خارجی منجر به کاهش فعالیت عضله درشت­نئی قدامی در طی فاز تماس پاشنه می­گردد ([Moisan & Cantin, 2016](#_ENREF_29)).

با بررسی مطالعات گذشته مشخص می­شود که اثرات طولانی­مدت استفاده از کفی حمایت­کننده قوس طولی داخلی پا بر متغیرهای کینماتیکی و کینتیکی راه رفتن در کوکان دارای کف پای صاف به لحاظ علمی مورد بررسی قرار نگرفته است. با توجه به اهمیت این موضوع، هدف پژوهش حاضر بررسی اثر استفاده طولانی­مدت (4 ماه) از ارتز حمایت­کننده قوس طولی­داخلی پا بر روی متغیرهای کینماتیکی و کینتیکی مفاصل اندام تحتانی طی راه رفتن در کودکان دارای کف پای صاف می­باشد.

ب ـ فرضيات و سوالات تحقيق

استفاده طولانی­مدت از کفی سبب تغییر در متغیرهای کینماتیکی مفاصل اندام تحتانی طی راه رفتن در کودکان دارای کف پای صاف می­گردد.

استفاده طولانی­مدت از کفی سبب تغییر در متغیرهای کینتیکی مفاصل اندام تحتانی طی راه رفتن در کودکان دارای کف پای صاف می­گردد.

استفاده طولانی­مدت از کفی سبب تغییر در مولفه­های نیروی عکس­العمل زمین طی راه رفتن در کودکان دارای کف پای صاف می­گردد**.**

ج ـ هدف از اجرا

هدف پژوهش حاضر بررسی اثر استفاده طولانی­مدت (4 ماه) از ارتز حمایت­کننده قوس طولی­داخلی پا بر روی متغیرهای کینماتیکی و کینتیکی مفاصل اندام تحتانی طی راه رفتن در کودکان دارای کف پای صاف می­باشد.

**د ـ دلايل توجيهي و ضرورت انجام طرح**

شیوع نسبتا بالای عارضه کف پای صاف در کودکان

عوارض شدیدی که این عارضه بر سایر مفاصل بدن دارا می­باشد

وجود ارتزهای مختلف در بازار و عدم وجود شواهد علمی بر تایید و یا عدم تایید استفاده از آن­ها

وجود تناقض در نتایج مطالعات گذشته

پژوهش حاضر از نوع نیمه­تجربی و آزمایشگاهی می­باشد. تاریخ فراخوانی آزمودنی­ها از 29/05/1396 (بعد از دریافت کد کمیته اخلاق) تا 13/06/1396 خواهد بود. از بین افراد واجد شرایط حداقل 30 نفر جهت شرکت در پژوهش از تاریخ سپتامبر 2017 (بعد از دریافت کد ثبت کارآزمایی بالینی) تا ژانویه 2018 خواهد بود. آزمودنی­ها از کلینیک تخصصی در شهر همدان، کشور ایران انتخاب خواهند شد. رضایت­نامه کتبی از والدین یا مسئول نگهداری کودکان دریافت خواهد شد. کد کمیته اخلاق از کمیته اخلاق در علوم پزشکی دانشگاه علوم پزشکی اردبیل دریافت خواهد شد. جهت تعیین این موضوع که آیا کودکان از کف پای صاف رنج می­برند، آزمون­های افت استخوانی، شاخص قوس پا (AHI)، و موقعیت استخوان پاشنه در وضعیت ایستاده استراحتی مورد آزمون قرار خواهد گرفت ([Bok et al., 2014](#_ENREF_6); [Cote, Brunet, II, & Shultz, 2005](#_ENREF_12); [Jung et al., 2009](#_ENREF_23)). شاخص AHI بدون واحد بوده و به عنوان نسبت ارتفاع قوس داخلی در 50 درصدی طول پا تقسیم بر طول پا از پاشنه تا سر اولین استخوان کف پایی تعریف می­شود ([AmirAli Jafarnezhadgero et al., 2018](#_ENREF_19)). ارتفاع AHI در وضعیت ایستاده در حالی که وزن بر روی هر دو پا قرار دارد، مورد سنجش قرار گرفت. افرادی که بیشتراز10میلی­متر افتادگی استخوان ناوی­کولار (اختلاف ارتفاع در دو شرایط با و بدون تحمل وزن) ([Cote, Brunet, et al., 2005](#_ENREF_12)) و اورژن پاشنه بیش از 4 درجه ([Bok et al., 2014](#_ENREF_6)) و شاخص قوس پا کمتر از 31/0 را دارا باشند ([Butler et al., 2008](#_ENREF_8))، به عنوان افراد دارای کف پای صاف معرفی خواهند شد ([Lange, Chipchase, & Evans, 2004](#_ENREF_25)). معیارهای خروج از پژوهش شامل سابقه­ی شکستگی، جراحی، کف پای صاف دارای نشانه درد، بیماری­های ارتوپدی، مشکلات عصبی­عضلانی، اختلاف طول اندام بیشتر از 5 میلی­متر و یا دارابودن فعالیت فیزیکی سنگین طی دو روز گذشته خواهد بود. پای برتر آزمودنی­ها توسط آزمون شوت نمودن توپ مشخص خواهد گردید ([A. A. Jafarnezhadgero, Majlesi, & Azadian, 2017](#_ENREF_20)).

پای غالب همه آزمودنی­ها سمت راست خواهد بود که با آزمون ضربه پا به توپ تعیین خواهد شد ([A. A. Jafarnezhadgero, M. M. Shad, et al., 2017b](#_ENREF_22)). دامنه سنی آزمودنی­ها بین 9 تا 12 سال خواهد بود. این افراد به طور تصادفی به دو گروه تجربی و کنترل تقسیم خواهند شد. گروه تجربی به مدت چهار ماه از کفی حمایت­کننده قوس طولی داخلی (بیشتر ارتفاع بخش داخلی آن 25 میلی­متر) در طی فعالیت­های روزانه استفاده خواهند نمود. در حالیکه گروه دارونما در این مدت از یک کفی تخت و بدون هیچگونه تغییر در سطح کفش استفاده خواهند.

در طی فرآیند انتخاب تصادفی نمونه­ها، یک پاکت استفاده خواهد شد تا شرایط هر گونه انتخاب هدفمند از بین برود. هیچ یک از والدین آزمودنی­ها یا آزمودنی­ها از گروه که بچه­ها در آن قرار خواهند گرفت آگاه نخواهند بود. یک فرد کاملا ناآگاه از شرایط و عنوان پژوهش مسئول انتخاب تصادفی نمونه­ها برای هر گروه خواهد بود.

استفاده از ارتز پا براساس پروتکل­های علمی خواهد بود که نشان داده­اند چگونه استفاده مفید از ارتز پا سبب پیشگیری از آسیب­های اندام تحتانی می­شود ([Christian John Barton et al., 2015](#_ENREF_2); [Witvrouw et al., 2014](#_ENREF_40)). مطالعات گذشته نشان داده­اند که استفاده از ارتز پا بهتر از این است که منتظر باشیم و ببینیم چه اتفاقی برای بیمار خواهد افتاد ([N. Collins et al., 2008](#_ENREF_9); [Mills et al., 2011](#_ENREF_28)). با توجه به اثرات اثبات شده ارتز پا در این پژوهش ما از این تداخل درمانی استفاده خواهیم نمود.

ابزارها

داده­های کینماتیکی با نرخ نمونه­برداری 100 هرتز با استفاده از 6 دوربین سیستم تحلیل حرکتی وایکان (Vicon system, Oxford Metrics, Oxford, UK) و 16 ماکر منعکس­کننده نور با قطر 15 میلی­متر ثبت خواهد شد. حجم فضای کالیبره 4*2*2 متر خواهد بود که در بخش میانی مسیر راه رفتن 15 متری قرار خواهد گرفت. مارکرست plugin gait برای معرفی لگن، ران، ساق و پا در دو سمت بدن استفاده خواهد شد. دو دستگاه صفحه نیرو کیستلر (Kistler, type 9281, Kistler Instruent AG, Winterthur, Switzerland) ثبت خواهد شد. دو صفحه­نیرو در مرکز فضای کالیبره واقع خواهند شد.

پروتکل استفاده از ارتز

گزارش شده است که نوع کفش بر روی الگوی راه رفتن در بچه­ها اثرگذار است ([Lythgo et al., 2009](#_ENREF_27); [Wolf et al., 2008](#_ENREF_41)). بنابراین، تمام آزمودنی­ها از نوع کفش مشابهی ([New Balance 759](http://www.newbalance.com/pd/new-balance-759/MW759.html?dwvar_MW759_color=Brown_with_Black_and_Orange)USA, ) بدون هیچونه اصلاحی در سطح داخلی آن در این پژوهش استفاده خواهند نمود. در گروه تجربی از ارتز پا حمایت­کننده قوس طولی­داخلی پا که از جنس اتیلن ونیل استات و لاستیک­های کوچگ سلولی خواهد بود جهت حفظ موقعیت مفصل سابتالار در وضیت طبیعی در گروه تجربی استفاده خواهد شد. در گروه تجربی، اوج ارتفاع قوس طولی داخلی ارتز پا در بخش میانی قوس پا 25 میلی­متر خواهد بود. در گروه کنترل، کفی با ضخامت 2 میلی­متر به عنوان شرایط دارونما استفاده خواهد شد که از جنس پلیستر خواهد بود.

پروتکل آزمایش

در طی پیش­آزمون، آزمودنی­ها با کفش و بدون استفاده از کفی در داخل آن راه خواهند رفت. بعد از پیش­آزمون، هر آزمودنی گروه تجربی ارتز پا را در طی 4 ماه در طی فعالیت­های روزانه خواهد پوشید. در طی دوره تداخل، از آزمودنی­های هر دو گروه خواسته می­شد تا به طور پیشرونده­ای زمان استفاده از ارتز پا را افزایش دهند. در روز اول، استفاده از ارتز پا به یک ساعت محدود خواهد شد تا فرآیند آَشنایی رخ دهد. در روز بعد، آزمودنی­ها یک ساعت زمان استفاده از کفی را افزایش خواهند داد تا در نهایت بتوانند کفی را در طی کل فعالیت­های روزانه استفاده نمایند ([Moisan & Cantin, 2016](#_ENREF_29)). مدت زمان استفاده از کفی روزانه ثبت خواهد شد. بعد از دوره تداخل، پس­آزمون دقیقا در همان دوره از روز که پیش­آزمون ثبت شد، ثبت خواهد شد.

جهت فیلتر نمودن داده­های کینماتیکی از فیلتر باترورث مرتبه چهارم با برش فرکانسی برابر 6 هرتز استفاده خواهد شد ([Farahpour et al., 2016](#_ENREF_14)). جهت فیلتر نمودن داده­های نیروی عکس العمل زمین از فیلتر باترورث با برش فرکانسی 20 هرتز استفاده خواهد شد ([Farahpour et al., 2016](#_ENREF_14)). ابتدا و انتهای فاز استقرار راه رفتن توسط دستگاه صفحه­نیرو مشخص خواهد شد، به این ترتیب که مقادیر بیشتر از 10 نیوتن نشان­دهنده تماس پاشنه با زمین و مقادیر کمتر از 10 نیوتن نشان­دهنده بلند شدن پنجه پا از زمین می­باشد. جهت محاسبه­ی مقادیر گشتاور مفاصل از روش دینامیک معکوس و توسط نرم­افزار پلیگان استفاده خواهد شد. جهت بررسی نرمال بودن توزیع داده­ها از آزمون شپیروویلک استفاده خواهد شد. جهت مقایسه درون­گروهی و بین­گروهی نتایج به ترتیب از آزمون آنالیز واریانس با اندازه­های تکراری در سطح معنی­داری 05/0 استفاده خواهد شد. تمام تحلیل­های آماری توسط نرم­افزار SPSS نسخه 21 انجام خواهد شد.
